# Glucose starvation mimetic aldometanib removes immune barriers permitting mice with hepatocellular carcinoma to live to normal ages

**DOI:** 10.1038/s41422-025-01195-4

**Published:** 2025-11-25

**Authors:** Hui-Hui Hu, Xuefeng Wang, Bin Lan, Haili Cheng, Hong Wen, Fangfang Chen, Jianfeng Wu, Mengqi Li, Jiazhou Chen, Jinhui Zhang, Dongxu Chen, Shiyu Lin, Jieyu Lin, Mingyang Yang, Zhenhua Wu, Zhong-Zheng Zheng, Fuqing Chen, Jianyin Zhou, Gang Chen, Yu Chen, Xianming Deng, Chen-Song Zhang, Jingfeng Liu, Sheng-Cai Lin

**Affiliations:** 1 State Key Laboratory of Cellular Stress Biology, State-Province Joint Engineering Research Center of Targeted Drugs from Natural Products, School of Life Sciences, Xiamen University, Xiamen, Fujian China; 2 Clinical Oncology School of Fujian Medical University, Fujian Cancer Hospital, Fuzhou, Fujian China; 3 Laboratory Animal Research Centre, Xiamen University, Xiamen, Fujian China; 4 The Zhongzhou Laboratory for Integrative Biology, School of Basic Medical Sciences, Henan University, Zhengzhou, Henan China; 5 Department of Hepatobiliary Surgery, Xiamen Key Laboratory of Translational Medicine for Digestive System Tumor, Fujian Provincial Key Laboratory of Chronic Liver Disease and Hepatocellular Carcinoma, Zhongshan Hospital of Xiamen University, Xiamen, Fujian China; 6 Department of Gastrointestinal Surgery, Institute of Gastrointestinal Oncology, Zhongshan Hospital of Xiamen University, Xiamen, Fujian China

**Keywords:** Cancer microenvironment, Nutrient signalling, Tumour immunology

## Abstract

Dysregulated metabolism in tumor tissues and para-tumor tissues alike can lead to immunosuppression, which may underlie cancer development. However, metabolic intervention as a therapeutic strategy has been of no avail. In this study, we explored the anti-cancer therapeutic effect of aldometanib, which specifically targets lysosome-associated aldolase to mimic glucose starvation and thereby activates lysosomal AMP-activated protein kinase (AMPK), a master regulator of metabolic homeostasis. We show that aldometanib inhibits the growth of hepatocellular carcinoma (HCC) in an AMPK-dependent manner, allowing hepatoma-bearing mice to survive to mature ages, although aldometanib does not possess cytotoxicity toward HCC or normal cells. Intriguingly, aldometanib exerts anti-cancer effects only in immune-competent host mice, but not in immune-defective mice. We also found that HCC tissues in aldometanib-treated mice were massively infiltrated with CD8^+^ T cells, which was not seen in mice with liver-specific knockout of *AMPKα*. Our findings thus suggest that the metabolic regulator AMPK rebalances the tumor microenvironment to allow cytotoxic immune cells inside the body to eliminate cancer cells and effectively contain the tumor tissues. The finding that metabolic intervention can make cancer a lifelong manageable disease may usher in a new era of cancer therapy.

## Introduction

Emerging evidence suggests that metabolic imbalance contributes to the development of cancers.^1,2^ Dysregulated metabolism leads to altered metabolite compositions and protein properties inside cellular compartments, as well as in the extracellular tumor microenvironment (TME) between the tumor and para-tumor tissues.^3^ One of the cancers that have been extensively studied for association with metabolic alterations is hepatocellular carcinoma (HCC).^4,5^ Metabolic alterations in glycolysis, the TCA cycle, nucleotide synthesis, amino acid metabolism, de novo lipogenesis, and cholesterol synthesis in HCC cells have all been implicated in the formation and development of HCC.^4^ Tremendous efforts have been made to identify chemical compounds that target various metabolic routes in search of effective therapies to treat cancers, including HCC, and some of the identified compounds have been undergoing clinical trials.^4,5^ For the same reason, AMPK, a master regulator of metabolic homeostasis that controls the overall metabolic network,^6–8^ has also been targeted for screening of clinically useful drugs for HCC treatment.^9,10^

AMPK is activated in response to physiological declines in glucose,^11^ reduced energy levels reflected by increased ratios of AMP:ATP or ADP:ATP,^12^ or global changes in cellular Ca^2+^ levels.^13^ Upon activation, AMPK phosphorylates a series of substrates to slow down anabolic activities such as the synthesis of fatty acids, cholesterol, and proteins, which are critical constituents of the building blocks for proliferation of both normal and cancer cells.^9,14^ Statins, either through inhibition of HMG-CoA reductase, a classic substrate of AMPK, to lower the synthesis of cholesterol^15–17^ or through direct activation of AMPK,^18–20^ have been shown to attenuate the development of HCC. Similarly, the compound ND-654 inhibits the development of HCC by inhibiting acetyl-CoA carboxylase (ACC, for de novo synthesis of fatty acids), another classic substrate of AMPK.^21^ Not surprisingly, efforts have been made to develop pharmacological agonists that directly target AMPK for potential efficacy in treating HCC. However, owing to the indiscriminate activation of all subcellular AMPK pools by such identified chemicals, most of these compounds may have adverse effects.^22^ For example, the pan-AMPK activator MK-8722 causes cardiac hypertrophy, although it robustly activates AMPK and lowers blood glucose.^23^ Similarly, although mitochondrial inhibitors activate AMPK by elevating AMP:ATP or ADP:ATP ratios, the inhibition of mitochondria per se may be harmful, particularly under prolonged treatments.^24,25^ Nevertheless, it is encouraging to see that AMPKβ1-specific agonists such as PXL770 and A769662 have shown effects in alleviating liver diseases such as fatty liver and nonalcoholic steatohepatitis (NASH).^26–28^

We previously delineated a pathway that senses declines in glucose, seen during physiological situations such as fasting, and transmits the low-glucose signal to activate AMPK on the surface of the lysosome^29–32^ and, importantly, to concomitantly inhibit the pro-anabolic mTORC1.^30,33^ This glucose sensing-AMPK pathway involves direct sensing of the availability of fructose-1,6-bisphosphate (FBP), a glycolytic intermediary metabolite that is a substrate of the glycolytic aldolase.^31^ Aldolase, when not occupied by FBP, acts to inhibit the endoplasmic reticulum-localized TRPV cation channels, which themselves, after inhibition, physically interact with and inhibit the lysosomal proton pump v-ATPase.^31,32^ Inactivated v-ATPase, along with conformational changes of its associated proteins, including the Ragulator complex, solicits translocation of the AXIN–LKB1 complex to the surface of the lysosome; the lysosomally localized AMPK is then phosphorylated by the co-translocated LKB1 and becomes activated.^30^ This route for activating the lysosomal pool of AMPK also enables the spatiotemporal regulation of metabolism,^34^ offering targets for identifying compounds that specifically activate lysosomal AMPK to mimic fasting states and elicit beneficial effects. The benefits of AMPK activation via this lysosomal pathway can be reflected by the fact that metformin at clinical doses takes a ride on this pathway to elicit various effects, including the alleviation of fatty liver.^35–37^

Using aldolase as a target, we identified an inhibitor termed aldometanib, which specifically prevents the v-ATPase-associated aldolase, but not the cytosolic aldolase involved in glycolysis, from binding to FBP even under high glucose, thereby mimicking a state of low glucose to activate AMPK via the lysosomal pathway.^38^ Depletion of factors involved in the lysosomal pathway in the liver, such as LAMTOR1 and AXIN, inhibits aldometanib-mediated activation of AMPK.^38^ In an AMPK-dependent manner, aldometanib alleviates hyperglycemia, fatty liver, and NASH in obese rodents.^38^

In this study, we asked whether aldometanib could possibly treat HCC by correcting metabolic dysregulation that may have been aggravated by pathological situations such as hepatitis or fatty liver, the most prevalent pre-conditions for the development of liver cancers. Although aldometanib does not exert cytotoxicity toward cells, we show that aldometanib drastically inhibits the growth and development of HCC in diethylnitrosamine (DEN)-treated and high-fat diet (HFD)-fed mice (DEN-HFD mice), mice with hepatic knockout of *Trp53* coupled with overexpression of Myc (*MYC*;*Trp53*^−/−^ HCC^39^), and orthotopic allografts established by HCC cell lines. Remarkably, treatment with aldometanib enabled the HCC-bearing DEN-HFD mice to live to mature ages. We also found that aldometanib treatment induces CD8^+^ cytotoxic T cells to massively surround and infiltrate the tumor tissues, but not the liver, with knockout of AMPK. These observations suggest that aldometanib activates AMPK to revert the tumor environment to a state that enables migration of immune cells, thereby acting as leverage for the immune system to defend against cancer development, enabling cancer-bearing mice to live to maturity.

## Results

### Aldometanib treatment enables HCC-bearing mice to live to normal ages

Mice were treated with DEN at 4 weeks of age and fed with an HFD starting at 7 weeks of age (DEN-HFD HCC;^40^ depicted in Supplementary information, Fig. S1b). From 40 weeks of age (35 weeks after DEN injection), the DEN-HFD mice were treated with aldometanib dissolved in drinking water. We first titrated to determine effective concentrations of aldometanib for AMPK activation in liver tissues (both HCC and para-HCC tissues) of these mice. Concentrations from 50 mg/L to 150 mg/L, which correspond to ~6–10 mg/kg/day calculated from the volume of daily intake of aldometanib-containing water (Supplementary information, Fig. S1a), effectively activated AMPK (as determined by the phosphorylation levels of AMPKα at Thr172 and ACC1 at Ser79; Supplementary information, Fig. S1b; note that higher p-AMPK and p-ACC signals were observed in tumor tissues, possibly due to lower glucose concentrations within the tumors, as reported previously^41^) and concomitantly inhibited mTORC1 (as determined by phosphorylation of the mTORC1 substrate S6K at its Thr389 site; Supplementary information, Fig. S1b). We also found that aldometanib at such doses did not affect energy levels, as evidenced by the unchanged ratios of AMP:ATP or ADP:ATP in both HCC and adjacent noncancerous tissues (Supplementary information, Fig. S1c), consistent with a previous report that aldometanib acts to activate AMPK via the lysosomal pathway, independently of the increase in AMP.^38^

**Fig. 1:**
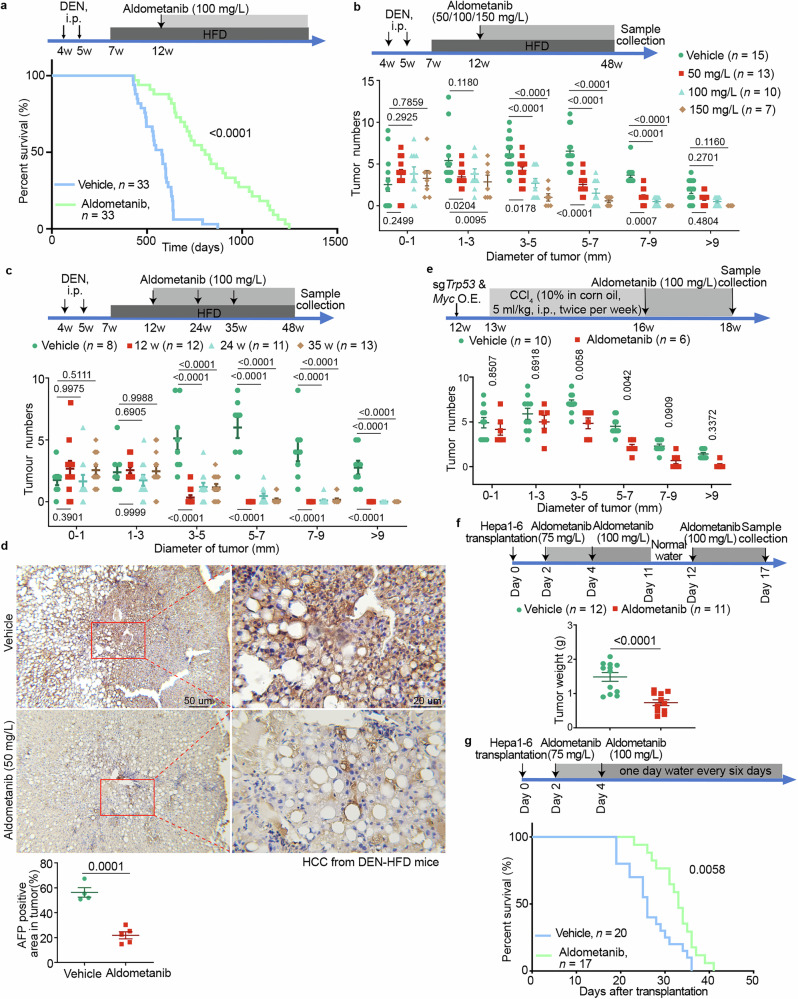
Aldometanib inhibits HCC. **a** Aldometanib treatment enables HCC-bearing mice to live to a similar age to normal mice. Wild-type C57BL/6 mice (4 weeks old) were intraperitoneally injected with DEN once a week for 2 weeks, followed by feeding with HFD 2 weeks later. The mice were then administered aldometanib at 100 mg/L in drinking water starting at 12 weeks of age. The lifespans of HCC-bearing mice are shown as Kaplan–Meier curves (see also statistical analyses in Supplementary information, Table S1). The average survival time of the aldometanib-treated DEN-HFD mice was 805 days, similar to the 796-day survival time of normal healthy mice as determined by us^38^ and others.^42^ **b** Aldometanib inhibits the growth of HCC in DEN-HFD mice. Mice were induced to develop HCC using DEN and HFD as in **a**. Aldometanib at 50 mg/L, 100 mg/L, or 150 mg/L was administered in drinking water starting at week 12 of age, and tissue samples were collected at week 48 (depicted in the upper panel). The numbers of tumors in each size/diameter category for each dosage of aldometanib are shown: 0–1 mm, 1–3 mm, 3–5 mm, 5–7 mm, 7–9 mm, and > 9 mm tumors (lower panel; shown as means ± SEM, *n* values indicate the number of mice used for each condition and are labeled in each panel; *P* values were calculated by two-way ANOVA followed by Tukey’s test). **c** Aldometanib effectively inhibits late-stage HCC. Mice were induced to develop HCC using DEN and HFD as in **a**. Aldometanib at 100 mg/L was administered in drinking water starting at 12 weeks, 24 weeks, or 35 weeks of age (depicted in the upper panel). Numbers of tumors in each size/diameter category for each entry time of aldometanib are shown (lower panel; shown as means ± SEM, *n* values represent the number of mice for each starting age and are labeled in each panel, with *P* values calculated by two-way ANOVA followed by Tukey’s test). **d** Aldometanib suppresses AFP levels in DEN-HFD mice. HCC tissues from aldometanib-treated and untreated DEN-HFD mice, prepared as in **b**, were subjected to immunohistochemistry (IHC) staining for AFP. Representative images at different magnifications are shown in the upper panel, and the percentages of AFP-positive area in the HCC tissues were calculated and are shown in the lower panel (means ± SEM, *n*  =  4 (vehicle) or 5 (aldometanib) mice for each treatment, with *P* values calculated by two-sided Student’s *t*-test). **e** Aldometanib inhibits HCC in *MYC*;*Trp53*^−/−^ mice. Wild-type BALB/c mice (12 weeks old) were hydrodynamically injected via the tail vein with plasmids carrying sgRNAs targeting *Trp53* for knockout, along with a plasmid for *Myc* overexpression. One week later, mice were intraperitoneally injected with CCl_4_ twice a week for 3 weeks (depicted in the upper panel). Aldometanib was administered at 100 mg/L in drinking water starting at 16 weeks of age. At week 18, mice were euthanized, and the numbers of tumors in each size/diameter category are shown (lower panel; shown as means ± SEM, *n* numbers are labeled in each panel, with *P* values calculated by two-way ANOVA followed by Sidak’s test). **f** Aldometanib reduces the size of orthotopic allografts derived from Hepa1-6 hepatoma cells. Hepa1-6 cells were transplanted into the left liver lobe of C57BL/6 mice to form orthotopic allografts. At day 2 post-transplantation, the mice were treated with aldometanib in drinking water for 15 days (depicted in the upper panel). On day 17, mice were euthanized, and the weights of the orthotopic allografts were determined (lower panel; shown as means ± SEM, *n* values represent the number of mice and are labeled in each panel, with *P* values calculated by two-sided Student’s *t*-test). **g** Aldometanib treatment increases the median lifespan of mice bearing Hepa1-6 allografts. Wild-type C57BL/6 mice were transplanted with Hepa1-6 cells as in **f**, and then treated with aldometanib in drinking water on day 2 post-transplantation (depicted in the upper panel). The lifespans of the mice were determined and are shown as Kaplan–Meier curves (see also statistical analyses in Supplementary information, Table S2). Experiments in this figure were performed three times.

We next determined the effects of aldometanib on the lifespan of the DEN-HFD mice. Strikingly, aldometanib at 100 mg/L, which we previously found to extend the lifespan of aged mice without causing noticeable adverse effects such as cardiac hypertrophy,^38^ extended the survival time of the HCC-bearing mice to normal ages compared to the control group (Fig. 1a; Supplementary information, Table S1). The treated HCC mice had a median lifespan of ~805 days, which was even slightly longer than the median survival time (796 days) of the healthy, untreated mice (Fig. 1a; refs. ^38,42^). Aldometanib also reduced the sizes and weights of the HCC tumors (Fig. 1b; Supplementary information, Fig. S1d–g; collected at 48 weeks of age as a representative time point). The suppression of HCC by aldometanib displayed a dose dependence, with mild inhibition at 50 mg/L and strong inhibition at 150 mg/L (Fig. 1b; Supplementary information, Fig. S1d–g). Importantly, aldometanib administered at late stages, as late as 30 weeks after DEN injection, when tumors had already formed (Supplementary information, Fig. S2a; see also ref. ^40^), still resulted in strong inhibition of HCC (Fig. 1c; Supplementary information, Fig. S2b–f). Alanine aminotransferase (ALT) and aspartate aminotransferase (AST) in the serum of DEN-HFD mice treated with aldometanib (Supplementary information, Fig. S1h and S2g) were significantly reduced, reflecting an improvement of liver damage. In addition, staining of alpha-fetoprotein (AFP), an indicator of metastasis severity and of the immunosuppressive tumor microenvironment of liver cancer,^43,44^ was also reduced in the HCC tumor sections (Fig. 1d). We also found a significant reduction in lipid droplets and triglyceride in the liver of aldometanib-treated DEN-HFD mice, along with improved glucose tolerance (Supplementary information, Fig. S3a–c), consistent with activation of AMPK by the drug. Aldometanib did not change the overall body fat mass, as the adipose tissue weights in the tumor-bearing mice before treatment were already low (Supplementary information, Fig. S3d), or liver fibrosis, which was barely detectable in these mice (Supplementary information, Fig. S3e), in contrast to our previous observations made on HFD-induced obese mice and AMLN-diet-induced NASH mice.^38^ These results indicate that aldometanib not only effectively contains HCC but also permits the HCC mice to live to mature ages.

We also tested the efficacy of aldometanib on another HCC model in which *Trp53* is knocked out and *Myc* is overexpressed, two of the most frequently altered genes in HCC patients, in the mouse liver (*MYC*;*Trp53*^−/−^ HCC^39^). Unlike the DEN-HFD HCC, the *MYC*;*Trp53*^−/−^ HCC did not involve the process of fatty liver formation.^39^ We titrated the dosage of aldometanib and selected 100 mg/L, which did not lead to a significant decrease in body weight in these lean mice before tumor formation, nor did it cause any AMPK-independent effects, as described in our previous study.^38^ Aldometanib at this dosage also suppressed the development of HCC in the *MYC*;*Trp53*^−/−^ mice (Fig. 1e; Supplementary information, Fig. S4a–f). We next generated orthotopic allografts derived from the hepatoma cell line Hepa1-6 (ref. ^45^) transplanted in the left lobe of the mouse liver. We found that aldometanib also significantly reduced the sizes of the orthotopic allografts (Fig. 1f; Supplementary information, Fig. S5a–f; doses titrated to ensure no significant impact on body weight; see also daily intake of aldometanib and glucose concentrations in allografts in Supplementary information, Fig. S5g–h). Moreover, aldometanib increased the median lifespan of these mice from 26 days to 33 days (a 26.9% improvement), even though the drug was applied after the formation of the allograft tumors (Fig. 1g; Supplementary information, Fig. S5i and Table S2). Consistent with previous studies showing that aldolase, particularly ALDOB, plays a role in inhibiting HCC growth^46–48^ and is found at lower levels in HCC patient tissues than in normal liver tissues,^49,50^ we observed a decrease in ALDOB in HCC cells, HCC patient tissues, and allografts (Supplementary information, Fig. S5j, k, n). However, aldometanib was still able to activate AMPK and inhibit mTORC1 in these cells and tissues (Supplementary information, Fig. S5l–n), as this is mediated by all three isozymes of aldolase, not only ALDOB.^38^ These data indicate that aldometanib showed strong effects on different HCC models, including reductions in tumor size and increased wellness of the mice, with the survival of HCC mice to mature ages as the most important efficacy endpoint.

### AMPK in non-cancerous host liver plays a dominant role in suppression of HCC by aldometanib

**Fig. 2:**
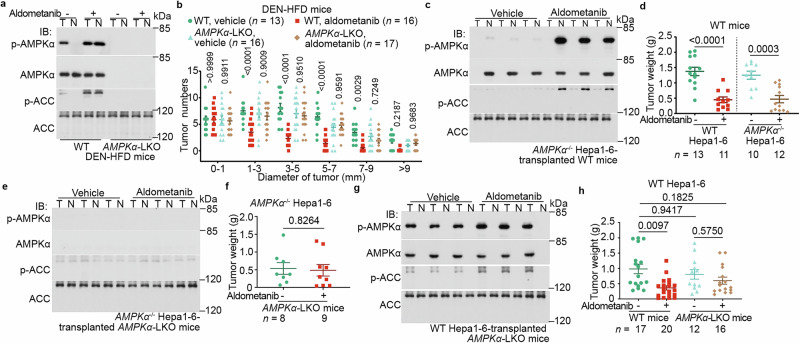
AMPK in para-HCC plays a dominant role in aldometanib-induced suppression of HCC. **a,** **b** Ablation of AMPK in the liver blocks the HCC-suppressing effects of aldometanib. C57BL/6 mice with liver-specific AMPKα knockout (*AMPKα*-LKO) and wild-type mice were induced to develop HCC using DEN and HFD, and then treated with aldometanib as in Fig. 1a. AMPK activity in the liver (**a**) and numbers of tumors in each size/diameter category were determined (**b**; shown as means ± SEM, *n* values represent the number of mice and are labeled in each panel; *P* values were calculated by two-way ANOVA followed by Tukey’s test). **c**–**h** Host liver AMPK plays a dominant role in the suppression of HCC orthotopic allografts by aldometanib. Wild-type (**c,** **d**) or *AMPKα*-LKO C57BL/6 mice (**e,** **f**) were transplanted with wild-type (**g,** **h**) or *AMPKα*^−/−^ (**c**–**f**) Hepa1-6 cells, and then treated with aldometanib as in Fig. 1f. AMPK activation (**c,** **e,** **g**) and tumor weights (**d,** **f,** **h**) were then determined (means ± SEM, *n* values represent the number of mice and are labeled in each panel; *P* values were calculated by two-sided Student’s *t*-test (**d,** **f**) or by two-way ANOVA followed by Tukey’s test (**h**)). Experiments in this figure were performed three times.

We next investigated the role of AMPK in the aldometanib-mediated suppression of HCC in mice. First, we generated DEN-HFD mice with liver-specific double knockout of *AMPKα1* and *AMPKα2* (*AMPKα*-LKO, generated as in ref. ^36^). The DEN-HFD treatment induced formation of HCC in the *AMPKα*-LKO mice at a similar onset to that in AMPK wild-type mice (Fig. 2b; Supplementary information, Fig. S6a). Interestingly, aldometanib did not suppress the development of HCC in liver lacking AMPK (Fig. 2a, b; Supplementary information, Fig. S6a–f), indicating that aldometanib indeed suppresses HCC progression by activating AMPK. To differentiate the role of AMPK in the HCC tissue from that in the noncancerous para-tumor liver tissue, we examined the effect of aldometanib on orthotopic allografts derived from *AMPKα*^−/−^ Hepa1-6 cells. We found that aldometanib could effectively inhibit the allograft tumors of *AMPKα*^−/−^ Hepa1-6 cells (validated in Supplementary information, Fig. S6g) transplanted in wild-type liver (Fig. 2c, d; Supplementary information, Fig. S6h–l) but not the orthotopic allografts grown in host liver lacking AMPKα (*AMPKα*-LKO; Fig. 2e, f; Supplementary information, Fig. S6m–q). By contrast, aldometanib only marginally impeded the growth of orthotopic allografts of wild-type Hepa1-6 cells when transplanted in the *AMPKα*-LKO host liver (Fig. 2g, h; Supplementary information, Fig. S6r–v). These results indicate that AMPK in the noncancerous para-tumor tissues plays a necessary and dominant role in the tumor suppression mediated by aldometanib.

### Aldometanib does not possess cytotoxicity

**Fig. 3:**
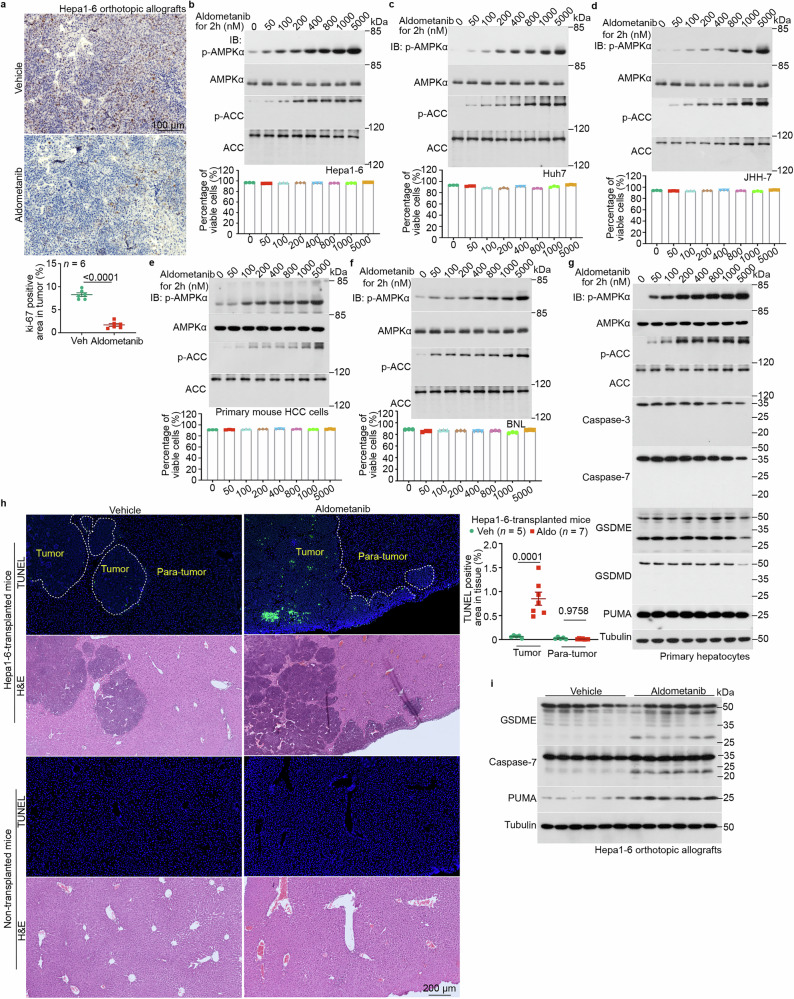
Aldometanib induces cytotoxicity specifically towards HCC tissues in vivo. **a** Aldometanib reduces the size of HCC orthotopic allografts derived from Hepa1-6 cells. HCC sections of orthotopic allografts derived from Hepa1-6 cells, generated as in Fig. 1f, were subjected to IHC staining for Ki-67. Representative images are shown in the upper panel, and the percentages of Ki-67-positive areas within the HCC tissues were calculated and are shown in the lower panel (means ± SEM, *n*  =  6 mice for each treatment, with *P* values calculated by two-sided Student’s *t*-test). Scale bar, 100 μm. **b**–**g** Aldometanib does not exhibit cytotoxicity towards liver cells in culture. Various liver cell types, including the mouse HCC cell line Hepa1-6 (**b**), human HCC cell lines Huh7 (**c**) and JHH-7 (**d**), primary mouse HCC cells (**e**), the normal mouse liver cell line BNL (**f**), and primary mouse hepatocytes (**g**), were treated with aldometanib at the indicated concentrations for 2 h. The percentages of viable cells (calculated by subtracting early apoptotic, late apoptotic, and necroptotic cells from the total cell count) were determined using flow cytometry (**b**–**f**; see gating strategy in Supplementary information, Fig. S7d, and representative density plots in Supplementary information, Fig. S7e–i; data are shown at the bottom of each panel as means ± SEM, *n* = 3 biological replicates for each condition). See also immunoblots of AMPK activation in the upper panels of **b**–**f** and the apoptotic and pyroptotic markers in **g**. **h,** **i** Aldometanib induces cell death inside HCC tissues. HCC tissues from allografts derived from Hepa1-6 cells, generated as in Fig. 1f, were subjected to TUNEL assays (**h**; representative images are shown in the left panel, and the percentages of TUNEL-positive areas in the tumor or para-tumor tissues were calculated and are shown in the right panel as means ± SEM; *n* values represent the number of mice and are labeled in each panel, with *P* values calculated by two-way ANOVA followed by Tukey’s test) or immunoblotting for apoptotic and pyroptotic markers (**i**). Scale bar, 200 μm. Experiments in this figure were performed three times.

We next explored how aldometanib inhibits the growth of HCC to enable the enhanced survival of HCC mice. We first investigated whether aldometanib inhibits cell growth in vitro. We used BNL cells, which are derived from normal liver tissue, share many properties with primary hepatocytes, and are able to proliferate in culture, unlike mouse primary hepatocytes.^51–54^ We found that aldometanib showed inhibitory effects on the proliferation of BNL cells at a concentration of 50 nM (Supplementary information, Fig. S7c), which was similar to the concentrations detected in the livers of DEN-HFD HCC mice or in orthotopic allografts grown in normal mice after administration of aldometanib at 100 mg/L (Supplementary information, Fig. S7a, b). Aldometanib inhibited the growth of various other HCC cells, including Hepa1-6, JHH-7, and Huh7 (Supplementary information, Fig. S7c), but the growth inhibition of primary mouse HCC cells was not significant (Supplementary information, Fig. S7c). In line with the inhibited proliferation of these cells, the growth of HCC orthotopic allografts derived from Hepa1-6 cells was also inhibited (Fig. 3a). We also determined the cytotoxic activity of aldometanib toward these cells in vitro, and we were surprised to find that aldometanib did not cause the death of HCC cells, based on the following observations. After testing for early apoptosis, we observed that concentrations of aldometanib from 50 nM to 100 nM — similar to the effective doses found in the liver of aldometanib-treated mice — did not trigger apoptosis in JHH-7 cells or primary mouse HCC cells after 2 h of treatment (Fig. 3d, e; Supplementary information, Fig. S7f, h). In fact, higher concentrations of aldometanib inhibited apoptosis in these cells (Fig. 3b–e; Supplementary information, Fig. S7e–h). Although 50 nM aldometanib caused a slight increase in early apoptosis in two other HCC cell lines (< 2% in Hepa1-6 cells and < 4% in Huh7 cells), higher concentrations reversed this effect (Fig. 3b, c; Supplementary Information, Fig. S7e, g). Moreover, none of the tested concentrations of aldometanib induced late apoptosis in any of the four HCC cell lines (Fig. 3b–e; Supplementary information, Fig. S7e–h). We also examined necroptosis and found that aldometanib did not induce necroptosis in these cells and even inhibited necroptosis in Huh7 cells at all tested doses (Fig. 3b–e; Supplementary information, Fig. S7e–h). In parallel, the total cell death and the percentage of viable cells showed little change at any of the aldometanib doses used (Fig. 3b–e). Aldometanib also had no cytotoxicity toward normal hepatocytes (Fig. 3f, g; Supplementary information, Fig. S7i), and extending the duration of aldometanib treatment to 12 h still did not result in cell death of these cell types (Supplementary information, Fig. S7j–m). In vivo, we assessed cell death by TUNEL assays and found that, after 2 weeks of aldometanib treatment, there was no increase in the TUNEL signal in para-tumor tissues of mouse livers carrying orthotopic allografts of Hepa1-6 cells (Fig. 3h). These results are consistent with previous studies showing that lysosomal AMPK activation actually inhibits apoptosis^55,56^ unless all AMPK pools are indiscriminately activated (e.g., by increased AMP).^57,58^ However, a robust increase in the TUNEL signal was observed in the HCC tissues (Fig. 3h), consistent with the suppressive effects of aldometanib on HCC in vivo. Levels of apoptotic markers, including cleavage of caspase-7 and protein levels of PUMA, were increased in the aldometanib-treated HCC tissues (Fig. 3i), and pyroptosis was also increased, as evidenced by elevated levels of the cleaved form of GSDME (Fig. 3i). The lack of cytotoxicity in cultured cells notwithstanding, these data demonstrate that aldometanib is able to cause cell death inside the HCC grown inside the mouse, implying that the drug can induce cytotoxicity in tumor tissues by an innate mechanism.

### Aldometanib induces infiltration of CD8^+^ T cells into tumors

**Fig. 4:**
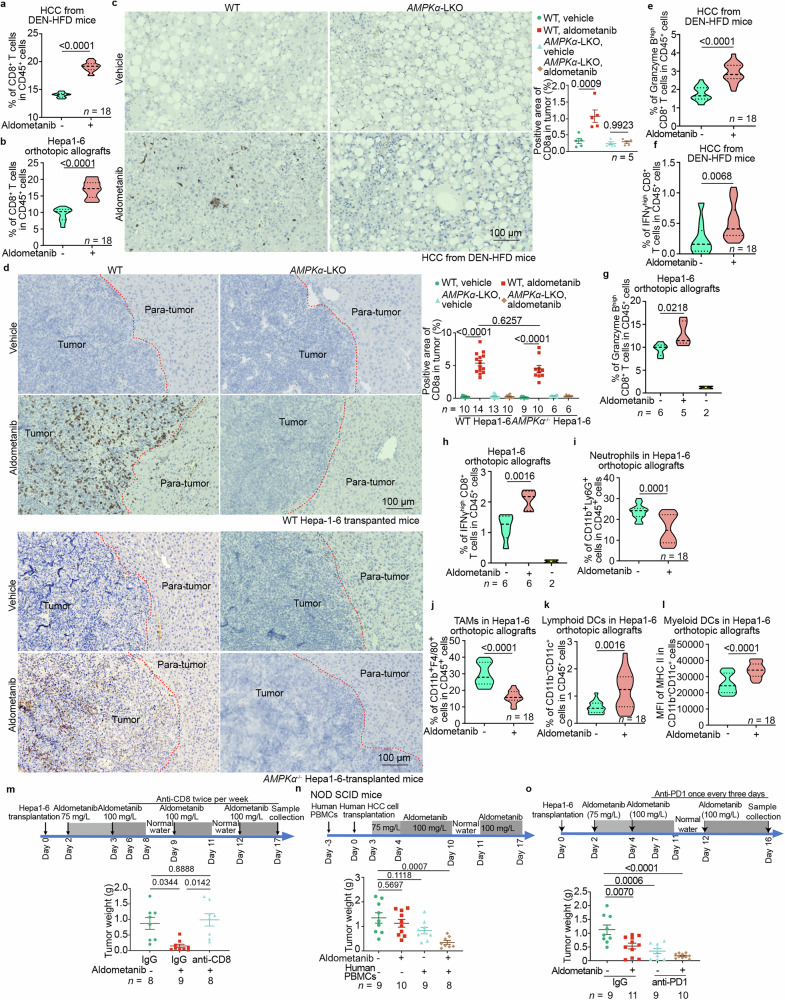
Aldometanib induces infiltration of CD8^+^ T cells into tumors. **a,** **b** Aldometanib promotes infiltration of CD8^+^ T cells into HCC tissues. The HCC tissues from DEN-HFD mice (**a**; prepared as in Supplementary information, Fig. S8a and collected at 40 weeks of age) or Hepa1-6-derived orthotopic allografts (**b**; generated as in Supplementary information, Fig. S8b and collected at day 10 post-transplantation) were digested with type I collagenase, and then stained with CD8a-Alexa Fluor 488, CD4-APC, and CD45-PerCP-Cy5.5 (**a**) or CD8a-APC and CD45-Alexa Fluor 488 (**b**) antibodies to label the CD8^+^ T cells. The stained cells were then subjected to flow cytometry analysis for quantification of CD8^+^ T cells. Data are shown as violin plots in which the lower and upper dashed lines represent the first and third quartile scores, respectively, the center dashed line represents the median, and the lower and upper limits denote the minimum and maximum scores, respectively. All subsequent violin plots follow the same format. *n*  =  18 samples from 6 mice in each condition, with *P* values calculated by two-sided Student’s *t*-test (**b**) or by two-sided Student’s *t*-test with Welch’s correction (**a**). See the gating strategy for the flow cytometry analysis in Supplementary information, Fig. S8a (**a**) and b (**b**), and the representative density plots in Supplementary information, Fig. S8c (**a**) and d (**b**). **c,** **d** AMPK is required for aldometanib-induced infiltration of CD8^+^ T cells into HCC tissues. HCC tissues from wild-type or *AMPKα*-LKO DEN-HFD mice (**c**) or from orthotopic allografts derived from wild-type or *AMPKα*^−/−^ Hepa1-6 cells transplanted into wild-type or *AMPKα*-LKO mice (**d**) were subjected to IHC staining for CD8a. Representative images are shown in the left panels, and the percentages of CD8a-positive areas within the tumor regions were calculated and are shown in the right panels (means ± SEM, *n* values represent the number of mice and are labeled in each panel; *P* values were calculated by two-way ANOVA followed by Tukey’s test). Scale bars, 100 μm. **e**–**h** Tumoricidal activities were detected in the CD8^+^ T cell-positive areas of HCC. HCC tissues from DEN-HFD mice (**e,** **f**) or Hepa1-6-derived orthotopic allografts (**g,** **h**; using the Hepa1-6-non-transplanted mouse liver, or naïve mouse liver, as a control) were digested and stained with CD8-Alexa Fluor 488 (**e,** **f**; as in **a**) or CD8-APC (**g,** **h**; as in **b**). Cells were further stained with granzyme B-PE (**e,** **g**) or IFNγ-PE (**f,** **h**) antibody to differentiate the sub-population of CD8^+^ T cells with high tumoricidal activity by flow cytometry analysis. Data are shown as violin plots; *n* = 18 samples from 6 mice (**e,** **f**) or *n* represents the number of mice as labeled in each panel (**g,** **h**); *P* values were calculated by two-sided Student’s *t*-test (**e**–**h**). See the gating strategy for the flow cytometry analysis in Supplementary information, Fig. S8a (**e,** **f**) and b (**g,** **h**), and the representative density plots in Supplementary information, Fig. S8j (**e**), l (**f**), k (**g**), and m (**h**). **i**–**l** Aldometanib eases the immune barriers in the HCC microenvironment. HCC tissues from Hepa1-6-derived orthotopic allografts were digested as in **b**. Cells were then stained with Ly6G-Alexa Fluor 488, CD45-PerCP-Cy5.5, and CD11b-APC antibodies to label neutrophils (**i**), CD11b-APC and F4/80-PE antibodies to label macrophages (**j**), CD11c-PerCP-Cy5.5, CD11b-APC, and CD45-Alexa Fluor 488 antibodies to label lymphoid DCs (**k**), and MHC II-PE, CD11c-PerCP-Cy5.5, and CD11b-APC antibodies to label MHC II-expressing myeloid DCs (**l**). Statistical analyses are shown as violin plots; *n*  =  18 samples collected from 6 mice, with *P* values calculated by two-sided Student’s *t*-test (**l**) or by two-sided Student’s *t*-test with Welch’s correction (**i**–**k**). See the gating strategy for the flow cytometry analysis in Supplementary information, Fig. S9a (**i,** **j**) and g (**k,** **l**), and the representative density plots in Supplementary information, Fig. S9b (**i**), c (**j**), h (**k**), and i (**l**). **m** Depletion of CD8^+^ T cells impedes aldometanib-induced suppression of HCC. Wild-type C57BL/6 mice were transplanted with wild-type Hepa1-6 cells, and then treated with aldometanib starting from day 2 post transplantation (depicted in the upper panel). Starting from day 6 post transplantation, the mice were intravenously injected with neutralizing antibodies against CD8^+^ T cells twice a week. HCC tissue samples were collected on day 17 post transplantation, and tumor weights were measured (means ± SEM, *n* values represent the number of mice and are labeled in each panel, with *P* values calculated by two-way ANOVA followed by Tukey’s test). See also the morphologies of HCC in Supplementary information, Fig. S10f (using H&E staining) and the validation data for the effectiveness of CD8^+^ T cell depletion in Supplementary information, Fig. S10g (using IHC staining). **n** Re-introduction of CD8^+^ T cells restores the ability of aldometanib to inhibit HCC in immunodeficient NOD-SCID mice. NOD-SCID mice were intraperitoneally injected with human PBMCs. At 3 days after the injection, mice were transplanted with human HCC cells into the left lobe of the liver. HCC tissue samples were collected on day 17, and tumor weights were measured (means ± SEM, *n* values represent the number of mice and are labeled in each panel, with *P* values calculated by two-way ANOVA followed by Tukey’s test). See also tumor:body weight ratios and the infiltration of CD8^+^ T cells in Supplementary information, Fig. S11m–r. **o** Aldometanib enhances the sensitivity of HCC to immunotherapy. Wild-type C57BL/6 mice were transplanted with wild-type Hepa1-6 cells, and then treated with aldometanib starting from day 2 post transplantation (depicted in the upper panel). Starting from day 7 post transplantation, the mice were intraperitoneally injected with anti-PD-1 antibody once every three days. HCC tissue samples were collected on day 16 post transplantation, and tumor weights were measured (means ± SEM, *n* represents the number of mice, with *P* values calculated by two-way ANOVA followed by Tukey’s test). Experiments in this figure were performed three times.

We next asked how aldometanib-induced AMPK activation inhibits the development of HCC tumors inside the mouse. As AMPK is a master regulator of metabolism, its activation may improve the composition of constituents in the microenvironment between the noncancerous and tumor tissues, as well as in the extracellular fluids,^59^ which can generate an immune barrier. It is well-established that cytotoxic lymphocytes, particularly the CD8^+^ T lymphocytes, act to induce the death of cancerous cells.^60^ To explore whether aldometanib affects the association with and infiltration of lymphocytes into the tumors, we next examined CD8^+^ T cells in HCC tissues from DEN-HFD mice or orthotopic allografts of Hepa1-6 cells in wild-type or *AMPKα*-LKO host liver. In both of the HCC tissues, aldometanib drastically enriched CD8^+^ T cells in tumors grown in wild-type host liver, in both intra-tumor and peripheral tumor tissues (Fig. 4a–d; Supplementary information, Fig. S8a–g; measured as both the absolute number of CD8^+^ T cells in the tumors and as the proportion of CD8^+^ T cells within a single-cell suspension generated from HCC tissue or the leukocyte population present in the HCC tissues; full scans; see also the data for validation of the antibody used to detect CD8a, a marker for CD8^+^ T cells, in Supplementary information, Fig. S8h), but not in the *AMPKα*-LKO host liver (Fig. 4c, d). By comparison, CD8^+^ T cells were rarely seen in the HCC from the vehicle (aldometanib-untreated) group (which exhibited sparse staining for CD8a; Fig. 4a–d; Supplementary information, Fig. S8a–g), consistent with previous studies reporting an immunosuppressive microenvironment in HCC.^61,62^ In addition, knockout of *AMPKα* in the host liver blocked such induced enrichment of CD8^+^ T cells in the orthotopic allografts from either wild-type or *AMPKα*^−/−^ Hepa1-6 cells (Fig. 4d), whereas depletion of *AMPKα* in the Hepa1-6 orthotopic allografts did not abolish the enrichment of CD8^+^ T cells (Fig. 4d). The enriched CD8^+^ T cells were activated, as evidenced by an increased proportion of CD62L-negative and CD44-positive (CD62L^−^CD44^+^) effector (cytotoxic) CD8^+^ T cells (T_eff_) and effector memory CD8^+^ T cells (T_em_), along with a decrease in CD62L^+^CD44^−^ naïve (antigen-inexperienced, unactivated) CD8⁺ T cells among the enriched population (Supplementary information, Fig. S8i). Aldometanib also significantly increased the number of central memory CD8^+^ T cells (CD8^+^ T_cm_; CD62L^+^CD44^+^) (Supplementary information, Fig. S8i). Furthermore, increased levels of granzyme B and IFNγ in the CD8^+^ T cells were observed in the aldometanib-treated HCCs (Fig. 4e–h; Supplementary information, Fig. S8j–m), indicative of enhanced tumoricidal effects consistent with enrichment of the CD8^+^ T cells. Aldometanib did not affect the numbers of stem-like CD8^+^ T cells (PD-1^+^CD44^+^Tim3^−^Tcf7^+^) or terminally exhausted CD8^+^ T cells (CD8^+^ T_ex_; PD-1^+^CD44^+^Tim3^+^Tcf7^−^) (Supplementary information, Fig. S8n), indicative of the presence of long-lasting anti-tumor immunity and an effective immune surveillance mechanism.

Consistent with the enhanced tumor infiltration by CD8^+^ T cells, aldometanib did not affect the maturation of CD8^+^ T cells in the thymus, as the mature thymic CD8^+^ single-positive T cells that can differentiate into various types of CD8^+^ T cells were not changed (Supplementary information, Fig. S8o). In addition, aldometanib slightly increased the populations of naïve CD8^+^ T cells and CD8^+^ T_cm_ in the spleen (Supplementary information, Fig. S8p; there was also a slight decrease in CD8^+^ T_em_ and terminally differentiated effector memory CD8^+^ T cells, or CD8^+^ T_EMRA_), suggesting that aldometanib may enhance the potential for CD8^+^ T cell cytotoxicity in the spleen.

Consistent with the increased infiltration of CD8^+^ T cells, we also observed a significant decrease in neutrophils and tumor-associated macrophages (TAMs) in the HCCs of mice treated with aldometanib (Fig. 4i, j; Supplementary information, Fig. S9a–e), both of which are known to act as major immune barriers in the tumor microenvironment, preventing the activation and infiltration of CD8^+^ T cells.^63,64^ Interestingly, aldometanib also helped enrich regulatory T cells (T_reg_) (Supplementary information, Fig. S9f), which are known to create immune barriers within the tumor microenvironment.^65^ However, the T_reg_ cells do not appear to block infiltration of CD8^+^ T cells into the aldometanib-treated HCCs, indicative of a context-dependent role of T_reg_ cells. In addition, the numbers of lymphoid dendritic cells (DCs) and the expression levels of major histocompatibility complex II (MHC II) in myeloid DCs,^66,67^ required for the activation (priming) of CD8^+^ T cells through the stimulation of the Th1 subtype of CD4^+^ T cells,^68,69^ were also increased upon aldometanib treatment (Fig. 4k, l; Supplementary information, Fig. S9g–i). We also observed that aldometanib increased, to some extent, the recruitment of Th2 cells, which are known to create an immunosuppressive environment in HCC,^70,71^ in 4 out of 18 samples analyzed (Supplementary information, Fig. S9j), although the number of Th2 cells was ~30-fold lower than that of Th1 cells. In addition, aldometanib did not promote the infiltration of HCC by Th17 cells, which are known to contribute to the activation of CD8^+^ T cells in tumor tissues (Supplementary information, Fig. S9j). Consistent with these results, the depletion of all these CD4^+^ T cell populations, including Th1, Th2, Th17, and T_reg_ cells, through repeated intravenous injections of a neutralizing antibody against CD4 did not significantly affect the suppression of HCC by aldometanib (Supplementary information, Fig. S9k, l; see also validation data for the efficacy of CD4^+^ T cell clearance by the anti-CD4 neutralizing antibody in Supplementary information, Fig. S9m), suggesting that these CD4^+^ T cell populations do not play a major role in the aldometanib-induced suppression of HCC. Furthermore, aldometanib promoted the infiltration of B cells (Supplementary information, Fig. S9n) and γδT cells (Supplementary information, Fig. S9o), both of which are known to help the recruitment and activation of CD8^+^ T cells in HCC.^72–76^ Importantly, when CD8^+^ T cells in mice were depleted by a neutralizing antibody, the aldometanib-enhanced enrichment of CD8^+^ T cells, the suppression of HCC, and the improvement of median lifespan were significantly reduced (Fig. 4m; Supplementary information, Fig. S10a–h). Furthermore, the suppression of HCC by aldometanib was greatly hindered in JHH-7 or Huh7 orthotopic xenografts grown in the livers of immunocompromised BALB/c nude mice lacking CD8^+^ T cells (Supplementary information, Fig. S11a–l). Similarly, when cells from human HCC tissues were transplanted into the livers of NOD-SCID mice, the tumor-suppressing effects of aldometanib were observed only in mice intraperitoneally injected with human peripheral blood mononuclear cells (PBMCs) containing CD8^+^ T cells from healthy donors (Fig. 4n; Supplementary information, Fig. S11m–r). In an additional control, we found that aldometanib did not significantly enhance the tumoricidal effects of previously activated CD8^+^ T cells toward Hepa1-6 cells when co-cultured in vitro (Supplementary information, Fig. S11s), reinforcing the conclusion that the suppression of HCC by aldometanib relies on its ability to promote the infiltration and activation of CD8^+^ T cells in vivo.

Aldometanib did not significantly promote the infiltration of NK cells (CD3^−^NK1.1^+^; Supplementary information, Fig. S12a, b). Although aldometanib was able to help recruit NKT cells (CD3^+^NK1.1^+^) into HCC tissues, the contents of the NKT cells remained lower than those in the livers of healthy mice (Supplementary information, Fig. S12a). We also observed no change in eosinophils and basophils before or after aldometanib treatment (Supplementary information, Fig. S12c, d). These data indicate that aldometanib stimulates the recruitment of CD8^+^ T cells into the HCC tumors to exert tumoricidal activity. In addition, aldometanib helps maintain the sensitivity of CD8^+^ T cells for potential immunotherapy, as expression levels of the immune checkpoint inhibitors Tim3 and Tcf7 on CD8^+^ T cells remained unchanged, and PD-1 expression was slightly increased after treatment (Supplementary information, Fig. S8n). Consistent with these findings, aldometanib significantly enhanced the sensitivity of HCC to immunotherapy, resulting in more pronounced death of HCC cells when combined with the anti-PD-1 antibody (Fig. 4o; Supplementary information, Fig. S12e).

## Discussion

We have shown that treatment with the non-cytotoxic glucose-starvation mimetic aldometanib can reduce tumor sizes in HCC spontaneously developed in DEN-HFD and *MYC*;*Trp53*^−/−^ mice and in orthotopic allografts of various origins, including Hepa1-6 and patient-derived HCC cells. Remarkably, aldometanib treatment enabled the tumor-carrying mice to live to normal ages, as observed in DEN-HFD mice. We have also demonstrated that AMPK in the para-tumor tissue plays a dominant role, as assessed in orthotopic allografts, to such an extent that even AMPK-null HCC can be effectively suppressed by aldometanib, as long as AMPK is present in the host tissue. This makes it unlikely that aldometanib-induced AMPK activation within tumor cells helps suppress tumor growth or clean tumor cells. The mild inhibitory effect of aldometanib on the proliferation of various HCC cells, possibly through reduced levels of anabolic activities after AMPK activation, also does not account for the reduction in tumor size, as the orthotopic allografts were formed prior to aldometanib administration.

We have also shown that although aldometanib does not possess cytotoxicity toward cancerous or normal cells, it robustly induces tumor cells to undergo apoptosis and necroptosis, signs of tumoricidal activity, and this effect depends on infiltration of the cytotoxic lymphocytes CD8^+^ T cells. As AMPK is known to play a central role in both anabolism and catabolism, influencing levels/functions of both metabolites and proteins, it is reasonable to suggest that aldometanib, via activation of AMPK, clears the way for mobilization of immune cells, particularly CD8^+^ T cells, to induce cytotoxicity in the tumor tissues. To explore what accompanies the promotion of CD8^+^ T cell migration, we analyzed chemokines such as CXCL9, CXCL10, and CXCL11, which are required for the infiltration of CD8^+^ T cells.^77–79^ Surprisingly, levels of these chemokines remained unchanged or even decreased after aldometanib treatment in both tumor and para-tumor tissues (Supplementary information, Figs. S13a–f, S14a–i). As a control, lipopolysaccharide (LPS) significantly increased the levels of these cytokines in the liver (Supplementary information, Fig. S14j). Our findings thus suggest that aldometanib, by causing metabolic changes that improve the microenvironment within the host tissues and/or the microenvironment between the tumor tissue and para-tumor tissue, does not rely on these cytokines to promote the infiltration of CD8^+^ T cells into HCC. Such a metabolic change may result from the combined effects of all these pathways. In addition, the improvement in the microenvironment may influence other immune cell subtypes to facilitate the infiltration of CD8^+^ T cells. Aldometanib treatment changed 15 out of 28 immune cell types/subtypes examined in HCC tissues, including neutrophils, TAMs, lymphoid DCs, myeloid DCs, CD8^+^ T_EMRA_ cells, CD8^+^ T_em_, naïve CD8^+^ T, CD8^+^ T_cm_, CD4^+^ Tregs, Th1, Th2, B, γδT, NK, and NKT cells, each of which is known to play a role in modulating the infiltration or tumoricidal effects of CD8⁺ T_eff_ cells.^80–83^ Furthermore, some immune cell subtypes remain to be characterized, such as the various types of TAMs that can exist in 9 different subtypes in the livers of NASH mice,^84^ which may assist CD8^+^ T cells in infiltrating HCC. The tumoricidal effector CD8^+^ T cells observed in HCC after aldometanib treatment appeared to be durably activated and not exhausted, an effect that could also be influenced by the enhanced microenvironment, particularly given that NASH-induced HCC is originally highly immunosuppressive.^85^ It has also been reported that the clonality of T cells, particularly the oligoclonal or polyclonal characteristics of CD8^+^ T cells that infiltrate HCC, plays a critical role in determining their tumoricidal effectiveness.^86,87^ It remains to be investigated how aldometanib, by improving the microenvironment, affects these systemic changes contributing to HCC suppression.

Current immunotherapies for HCC are often restricted to specific etiologies or subtypes of HCC,^88^ because these therapies must be tailored to restore the particular types of immune cells that are dysregulated in a specific HCC etiology in order to counteract immunosuppression. For example, NASH-induced HCCs often exhibit increased levels of IL-10, one of the cytokines that can suppress CD8^+^ T cells, which is secreted by circulating IgA^+^ plasmocytes.^89^ In hepatitis B virus-induced HCC, IL-10 is mainly produced by intrahepatic Breg cells,^90,91^ whereas in hepatitis C virus-induced HCC, it is mainly produced by circulating CD4^+^CD25^+^ Treg cells.^92^ In addition, in NASH-induced HCCs, a specific subset of T cells, known as PD-1^+^CXCR6^+^CD8^+^ T cells, plays a crucial role in creating an immunosuppressive TME and hindering the response to immune checkpoint inhibitors.^85,93^ In this study, we examined HCCs of DEN-HFD mice and *MYC*;*Trp53*^−/−^ mice and orthotopic allografts of various origins, including Hepa1-6 and patient-derived HCC cells. We showed that aldometanib can elicit inhibitory effects on all tested HCC types in an AMPK-dependent manner. It is possible that AMPK activation by aldometanib may improve the overall metabolic network to clear the way for CD8^+^ T cells to migrate and reach various types of HCC. Consistent with this notion, we and others have shown that caloric restriction (CR) or metformin, which can activate AMPK in the liver,^94,95^ shows some effects on the progression of HCC in mice (ref. ^96–101^; see also Supplementary information, Fig. S15a–h). Also of some relevance, metformin was shown to enhance the efficacy of immunotherapies.^102^ However, mouse models for HCC may have limitations in replicating the entire process of development and metastasis of HCC in humans, as the DEN-HFD and *MYC*;*Trp53*^−/−^ HCCs were both non-metastatic, and the Hepa1-6 allografts demonstrate only limited metastatic ability.^103,104^ In the case of Hepa1-6, aldometanib does not affect the invasiveness or migration of these cells in vitro, nor does it influence the lung metastasis of the allografts in vivo (Supplementary information, Fig. S15i–k). In addition, aldometanib does not alter the proliferation rates and intrahepatic metastasis of residual Hepa1-6 cells derived from the allografts after treatment, nor does it impact their susceptibility to cytotoxicity from CD8^+^ T cells (Supplementary information, Fig. S15l–n). It also does not affect the sensitivity of these residual Hepa1-6 cells to immunotherapy (mediated by anti-PD-1 antibody) or chemotherapy (using oxaliplatin) (Supplementary information, Fig. S15o–r). Moreover, it remains unclear whether aldometanib plays a role in suppressing HCC during its metastasis; further investigation is warranted. In short, we have demonstrated that metabolic intervention, as exemplified by the application of aldometanib, can allow cancers to become a manageable disease, enabling cancer patients to live with cancer (Supplementary information, Fig. S15s).

## Materials and methods

### Data reporting

The chosen sample sizes were similar to those used in this field: *n* = 4–8 samples were used to evaluate the levels of metabolites in serum^36,38,105^ and tissues;^31,36,38^ *n* = 4–15 samples to determine the mRNA levels of a specific gene;^30,38,40,106^ *n* = 3–6 samples to determine the expression levels and phosphorylation levels of a specific protein;^21,30,36^ *n* = 6–15 mice to induce HCC;^21,39,40,107,108^ *n* = 6 mice to determine hepatic TAG;^27,36,38^ *n* = 3–5 mice to determine the formation of HCC using magnetic resonance imaging (MRI);^85,109,110^ *n* = 4–18 samples for the flow cytometry analysis of immune cells;^107,108^ *n* = 3–14 mice for immunohistochemistry (IHC) and H&E staining;^21,38,107,108^ *n* = 10–33 mice to generate survival curves of tumor-bearing mice;^21,39,40^ *n* = 6–15 mice for determination of serum ALT and AST;^38,40^ and *n* = 4 mice for glucose tolerance tests.^21,38^ No statistical methods were used to predetermine the sample size. All experimental findings were repeated as stated in the figure legends, and all additional replication attempts were successful. For animal experiments, mice were housed under the same conditions or place. For cell experiments, cells of each genotype were cultured under the same conditions and were seeded in parallel for different treatments. Each experiment was designed and performed along with proper controls, and samples for comparison were collected and analyzed under the same conditions. Randomization was applied wherever possible. For example, during mass spectrometry (MS) analyses (metabolites and pharmacokinetics), samples were processed and subjected to MS in random order. For animal experiments, age-matched littermates of each genotype were randomly assigned to aldometanib treatments. In cell experiments, cells were randomly assigned to different treatments. Similarly, during microscopy data collection and statistical analyses, the fields of view were chosen on a random basis, often by different operators, preventing potentially biased selection for desired phenotypes. Otherwise, randomization was not performed. For example, when performing immunoblotting, samples needed to be loaded in a specific order to generate the final figures. Blinding was applied wherever possible. For example, during sample collection and processing, samples or cages were labeled with code names that were later revealed by the individual who picked and treated the animals or cells but did not participate in sample collection and processing until assessing the outcome.

### Mouse strains

Protocols for all rodent experiments were approved by the Institutional Animal Care and Use Committee of Xiamen University (XMULAC20180028 and XMULAC20220050). Unless otherwise stated, mice were housed with free access to water and a standard diet (65% carbohydrate, 11% fat, 24% protein) under specific pathogen-free conditions. The light was on from 8:00 to 20:00, with the temperature kept at 21–24 °C and humidity at 40%–70%. Only male mice were used, and male littermate controls were used throughout the study.

For CR, mice were individually caged for 1 week before the treatment. Each mouse was fed 2.5 g of the standard diet (~70% of ad libitum food intake for a mouse at 8 weeks old or older) at 5 p.m. each day.

Wild-type C57BL/6J (Cat# 000664) and BALB/c (Cat# 000651) mice were obtained from the Jackson Laboratory, and BALB/c nude mice (Cat# 401) and NOD-SCID mice (Cat# 394) from the Charles River Beijing branch (Vitalriver). *AMPKα1*^F/F^ (Cat# 014141) and *AMPKα2 *^F/F^ mice (Cat# 014142) were obtained from the Jackson Laboratory and provided by Dr. Sean Morrison. *AMPKα*-LKO mice were generated by crossing *AMPKα1*/*2*^F/F^ mice with *Alb*-*Cre* mice, as described previously.^38^ The primers used for genotyping the *AMPKα*-LKO mice were as follows: 5′-CCCACCATCACTCCATCTCT-3′ and 5′-AGCCTGCTTGGCACACTTAT-3′ for *AMPKα1*; 5′-GCAGGCGAATTTCTGAGTTC-3′ and 5′-TCCCCTTGAACAAGCATACC-3′ for *AMPKα2*; and 5′-ATGAAATGCGAGGTAAGTATGG-3′ and 5′-CGCCGCATAACCAGTGAAAC-3′ for *Alb*-*Cre*.

Mice of the following ages were used: (1) to generate HCC xenograft-bearing mice: 8-week-old wild-type or *AMPKα*-LKO C57BL/6 J mice (for transplanting Hepa1-6 cells), 8-week-old NOD-SCID mice (for transplanting human patient-derived HCC cells), or 8-week-old BALB/c nude mice (for transplanting Huh7 and JHH-7 cells); (2) to induce DEN-HFD HCC: 4-week-old wild-type or *AMPKα*-LKO C57BL/6 J mice; (3) to induce *MYC*;*Trp53*^*−*/−^ HCC: 12-week-old wild-type BALB/c mice; (4) to analyze AMPK activation, adenylate levels, and hepatoma phenotypes in liver tissues: 41-week-old DEN HCC mice (treated with either vehicle or aldometanib dissolved in drinking water for 1 week, starting at 40 weeks of age), 10-week-old Hepa1-6-derived xenograft-bearing mice (also treated with either vehicle or aldometanib for 1 week, starting from day 2 after cell transplantation), or 16-week-old *MYC*;*Trp53*^−/−^ mice (treated similarly starting from 15 weeks of age); (5) to determine the concentrations of aldometanib in serum and liver tissues: 41-week-old DEN HCC mice (treated as above) and 10-week-old Hepa1-6-derived xenograft-bearing mice (treated as above); (6) to assess the composition of immune cells in HCC tissues: 10-week-old Hepa1-6-derived xenograft-bearing mice (treated with either vehicle or aldometanib for a total of 8 days, starting from day 2 after cell transplantation) and 40-week-old DEN-HFD mice (treated for 8 weeks starting from 32 weeks of age); (7) to analyze chemokine levels using RT-PCR or RNA sequencing: 10-week-old Hepa1-6-derived xenograft-bearing mice (treated for a total of 8 days, starting from day 2 after cell transplantation), 40-week-old DEN-HFD mice (treated for 8 weeks starting from 32 weeks of age), and 8-week-old wild-type C57BL/6 J mice; (8) to analyze fat composition: 48-week-old DEN-HFD mice (treated for 36 weeks starting from 12 weeks of age); (9) to analyze glucose tolerance: 40-week-old DEN-HFD mice (treated for 28 weeks starting from 12 weeks of age); (10) to analyze glucose concentration: 11-week-old Hepa1-6-derived xenograft-bearing mice (treated for a total of 15 days, starting from day 2 after cell transplantation); and (11) to isolate primary hepatocytes: 4-week-old wild-type mice.

### Formulation of aldometanib

Aldometanib was formulated as described previously.^38^ In brief, for cell-based experiments, aldometanib powder was dissolved in DMSO, aliquoted, and stored at 4 °C. The solution was incubated in a 37 °C water bath for 10 min (until no precipitate was visible) before addition to the culture medium. For mouse experiments, aldometanib was formulated in 10% (w/v) Kolliphor HS 15 before the experiment. In brief, to prepare 3 L of 1 mg/mL aldometanib stock solution, 3 g of aldometanib was dissolved in 300 mL of ethanol, and then mixed with 300 g of Kolliphor HS 15. The mixture was stirred at room temperature until the Kolliphor HS 15 was completely dissolved. The ethanol was then evaporated using a rotary evaporator (Rotavapor R-300, BUCHI) at 90 rpm and 45 °C, followed by mixing with 2.7 L of water. The control vehicle was prepared similarly, with no added aldometanib. Both the aldometanib solution and the vehicle were stored at room temperature and used within 1 month.

### Isolation of patient-derived HCC cells

Patient HCC tissues were obtained from the Fujian Cancer Hospital, except for the human HCC and para-HCC samples shown in Supplementary Information, Fig. S5j, which were collected from Zhongshan Hospital affiliated with Xiamen University. All procedures were conducted in accordance with the Declaration of Helsinki and were approved by the Human Research Ethics Committee of Zhongshan Hospital (XMZSYYKY 2022-072) and the Tumor Ethics Committee of Fujian (K2023-003-01). All participants signed an informed consent form before enrolment. The collected HCC tissues showed a macrotrabecular-massive type and tested positive for cirrhosis. As determined by IHC staining, the HCC tissues also tested positive for Arg-1, CD34 (vascular expression only), and GS, but negative for HepPar-1, CK7, CK19, and CD10. Approximately 30% of the nuclei were positively labeled for Ki-67, with partial positivity for HSP70 and Glypican-3.

The patient HCC tissues were collected and maintained in NOD-SCID mice as described previously,^111–113^ with minor modifications. In brief, freshly excised HCC tissues were placed in serum-free DMEM in an ice bath, and sliced to a diameter of 1–2 mm at the surgical site. After washing with ice-cold serum-free DMEM 2 times, tissues were subcutaneously implanted into the right flanks of 8-week-old NOD-SCID mice. The tumor tissues were collected and sliced to 1 mm in diameter after reaching ~1000 mm^3^ in the NOD-SCID mice, and then digested with 10 mL of type I collagenase (1 mg/mL final concentration in serum-free DMEM) in a petri dish at 37 °C and 60 rpm for 10 min on an orbital shaker. After the digested tissue was passed through a 70-μm cell strainer (Cat# 352350; BD Falcon), cells were centrifuged at 400× *g* for 5 min at room temperature, then suspended in serum-free DMEM and transplanted into the livers of NOD-SCID mice as described below in “HCC-HFD, *MYC*;*Trp53*^−/−^ mice and HCC orthotopic allografts” section.

### HCC-HFD, *MYC*;*Trp53*^−/−^ mice and HCC orthotopic allografts

The DEN-HFD mouse HCC model was established as described previously,^40^ with minor modifications. In brief, C57BL/6J mice were given two intraperitoneal injections of 80 mg/kg DEN, one on postnatal day 28 and the other on postnatal day 35. Two weeks after the second injection, the mice were switched to HFD (60% calories from fat; Cat# D12492, Research Diets) for another 41 weeks (except for the analysis of AMPK activation and AMP levels in the HCC tissues, for which the duration of HFD feeding was 34 weeks, as shown in Supplementary information, Fig. S1b, c). Mice were then administered aldometanib in the drinking water. At the end of the study, the mice were euthanized, and their liver tissues were collected for analysis of tumor diameter using Vernier calipers (Cat# 150 T, Meinaite Tools, China).

The *MYC*;*Trp53*^−/−^ HCC mice were established as described previously,^39,108^ with minor modifications. In brief, 12-week-old BALB/c mice were hydrodynamically injected with a plasmid mixture consisting of 30 μg of pX330-sg-p53 (ref. ^114^; #59910, Addgene, gift from Dr. Tyler Jacks), 30 μg of PT3-EF1a-Myc (#92046, Addgene, gift from Dr. Xin Chen), and 7.5 μg (4:1 ratio) of pCMV/SB10 transposase-encoding plasmids (#24551, Addgene, gift from Dr. Perry Hackett), all freshly dissolved in 2 mL of 0.9% NaCl (w/v) solution, through the tail vein. For each mouse, a total volume of mixture corresponding to 10% of body weight was injected into the lateral tail vein within 7 s. The mice were then intraperitoneally injected with 10% (v/v in corn oil) CCl_4_ at a dose of 5 mL/kg twice per week for 3 weeks, starting 1 week after hydrodynamic tail-vein injection.

The Hepa1-6-derived hepatoma orthotopic allografts (orthotopic implantation) were established in situ as described previously,^115–117^ with minor modifications. In brief, the Hepa1-6 cells were trypsinized, washed with serum-free DMEM, and then resuspended in serum-free DMEM. Approximately 1.5 × 10^6^ wild-type Hepa1-6 cells or 2 × 10^6^ *AMPKα*^−/−^ Hepa1-6 cells, suspended in 40 μL of serum-free DMEM, were slowly injected into the left liver lobe of each 8-week-old C57BL/6 J mouse. After injection, the liver surface at the needle site was gently covered with a sterile cotton swab for ~2 min to minimize bleeding and potential backflow/leakage. The mice were then administered aldometanib in the drinking water as described in Fig. 1f, followed by analysis of tumor sizes at the indicated time points. Hepatoma orthotopic allografts derived from human HCC cell lines (JHH-7 and Huh7) were established similarly, except that 1 × 10^7^ cells were transplanted into 8-week-old BALB/c nude mice. The mice were then administered aldometanib in the drinking water as described in Supplementary Information, Fig. S11a, g, followed by analysis of tumor sizes at the indicated time points.

To deplete the CD8^+^ T or CD4^+^ T cells in the Hepa1-6-derived xenograft-bearing mice, 200 μg of a neutralizing antibody against CD8a or CD4 was intravenously injected into the mice twice a week, starting from day 6 post cell transplantation, followed by treatment with aldometanib as described in Fig. 4m, Supplementary information, Figs. S9k, S10h. The efficiency of CD8^+^ T cell depletion or CD4^+^ T cell depletion was confirmed by IHC on day 17 (Supplementary information, Fig. S10g) or day 16 (Supplementary information, Fig. S9m) after HCC cell transplantation.

To evaluate the immunotherapeutic effects of the anti-PD-1 antibody on Hepa1-6 allografts, 50 μg of the antibody was injected intraperitoneally into the mice every 3 days, starting on day 7 after HCC cell transplantation, followed by treatment with aldometanib as described in Fig. 4o.

The patient-derived HCC orthotopic allografts were established as described previously,^118–120^ with minor modifications. In brief, 8-week-old NOD-SCID mice were each injected with 2 × 10^7^ PBMCs that were freshly prepared from the whole blood of healthy human donors. PBMCs were prepared by mixing the blood with PBS in a 1:5 ratio, and 5 mL of the mixture containing PBMCs was transferred onto the upper layer of 3 mL of Ficoll, followed by centrifugation for 20 min at 2000× *g* and 25 °C. The centrifuge was set to minimum acceleration, and the brake program was deactivated, as it would otherwise disturb the Ficoll gradient and, in turn, lower the cell yield. After centrifugation, ~1 mL of the fraction located above the Ficoll surface, which contained the PBMCs, was collected using a Pasteur pipette. This fraction was mixed with 10 mL of PBS and centrifuged again for 5 min at 400× *g* and 25 °C. At 3 days after PBMC injection, 1 × 10^7^ human HCC cells, suspended in 40 µL of serum-free DMEM, were transplanted into the left lower lobe of the mouse liver. Mice were then treated with aldometanib in the drinking water, as in Fig. 4n, followed by analysis of tumor sizes.

Note that liver tissue images were not collected throughout the study. This is because AMPK activity is highly sensitive to the time elapsed after tissue dissection.^34^ At the conclusion of liver-tissue imaging, AMPK would have been fully activated not only by the glucose-sensing pathway but also by AMP- and calcium-dependent pathways, mimicking severe stress such as ischemia.^34,121^ Such activation would compromise our ability to assess AMPK activation in the liver at the time of sample collection, which we routinely do to determine the effectiveness of aldometanib.

### Evaluation of the lifespan of HCC-bearing mice

The survival lifespans of DEN-HFD mice and Hepa1-6-derived allograft-bearing mice were determined as described previously,^38,40^ with minor modifications. In brief, mice were examined every morning for signs of illness, and severely moribund mice were censored. A mouse was considered severely moribund if it showed more than one of the following clinical signs: (1) inability to eat or drink; (2) severe lethargy, as indicated by lack of response, such as reluctance to move when gently prodded with a blunt-tip tweezer; or (3) severe balance instability or gait disturbance. Mice found dead were also recorded at each daily inspection. Aldometanib was administered starting at 12 weeks of age and continued throughout the lifespan of the DEN-HFD mice, or starting on day 2 post transplantation of Hepa1-6 cells.

In the cohort shown in Fig. 1a and Supplementary information, Table S1, the experiment started with a total of 104 male mice: 53 in the vehicle group and 51 in the aldometanib group. During the experiment, 38 mice were removed (censored) from the study (20, vehicle; 18, aldometanib). The reasons for removal included fighting (6, vehicle; 3, aldometanib), paralysis (loss of walking ability: 5, vehicle; 6, aldometanib), severe ulcerative dermatitis (5, vehicle; 4, aldometanib), and symptoms of gnawing or bruxing (the presence of long, spiral incisors that prevented the mouse from eating: 4, vehicle; 5, aldometanib). Such censored mice were not included in the lifespan calculations. In the cohort shown in Fig. 1g and Supplementary information, Table S2, the experiment started with a total of 37 male mice: 20 in the vehicle group and 17 in the aldometanib group. Throughout the experiment, no mice were removed (censored) from the study.

In the cohort shown in Supplementary information, Fig. S10h and Table S3, the experiment started with a total of 30 male mice: 10 in the vehicle group, 10 receiving aldometanib, and 10 receiving aldometanib together with a neutralizing antibody against CD8a. No mice were removed (censored) during the experiment.

### MRI

To detect the local growth of hepatoma in DEN-HFD mice, liver MRI was performed using a 9.4-T magnetic resonance scanner (Biospec 94/20 USR, Bruker) as described previously.^110,122^ In brief, the mouse was anaesthetized and placed in a prone position with its extremities extended on the imaging bed. After positioning the mouse coil at the iso-centre of the scanner, imaging was performed using a T2-weighted rapid acquisition with relaxation enhancement (RARE) sequence. The parameters were as follows: (1) echo times: 33 ms; (2) repetition time: 2500 ms; (3) averages: 2; (4) repetitions: 1; (5) echo spacing: 11 ms; (6) rare factor: 8; (7) slices: 20; (8) slice orientation: axial; (9) slice thickness and distance: 1 mm; (10) image size: 256 × 256; (11) field of view: 35 × 35 mm; (12) echo images: 1; (13) excitation angle: 90°; (14) refocusing angle: 180°; (15) dummy scans: 2; (16) dummy duration: 5000 ms; (17) motion averaging, flip back, and fat suppression: ticked; (18) dimension: 2D; (19) isotropic: off; (20) anti aliasing: 1 × 1; (21) object ordering mode: interlaced; (22) read offset: 2.023 mm; (23) phase offset: −2.262 mm; (24) slice offset: 0.792 mm; (25) slice gap mode: non-contiguous; (26) bandwidth: 36,764.7 Hz; (27) excitation pulse and refocusing pulse: calculated; and (28) auto repetition spoiler and auto echo spoiler: ticked.

### Cell lines

No cell lines used in this study are listed as misidentified by the International Cell Line Authentication Committee (https://iclac.org/databases/cross-contaminations/). HEK293T (Cat# CRL-3216), Hepa1-6 (Cat# CRL-1830), and BNL (Cat# TIB-73) cells were purchased from ATCC; Huh7 cells (Cat# CL-0120) from Procell; and JHH-7 (Cat# CTCC-004-0053) from Meisen CTCC. HepaRG (Cat# IM-H415) cells were purchased from IMMOCELL. All cells were verified to be free of mycoplasma contamination and authenticated by STR sequencing. All cell lines were maintained in DMEM supplemented with 10% FBS, 100 IU penicillin, and 0.15 mg/mL streptomycin, except for JHH-7 cells and HepaRG cells, which were maintained in DMEM/F-12 medium and HepaRG cell medium (Cat# IM-H415-1), respectively (also supplemented with 10% FBS, 100 IU penicillin, and 0.15 mg/mL streptomycin). Cells were cultured at 37 °C in a humidified incubator containing 5% CO_2_.

The *PRKAA1* and *PRKAA2* genes were deleted from Hepa1-6 cells using the CRISPR-Cas9 system. The sgRNAs were designed as described previously.^123^ Nucleotides were annealed to complements containing the cloning tag aaac and inserted into the back-to-back *Bsm*BI restriction sites of the lentiCRISPRv2 vector (#52961, Addgene). The sequences of the sgRNAs were as follows: 5′-GGGCCGCAATAAAAGATATC-3′ for mouse *PRKAA1* and 5′-GGGAGCCCGTGCGCCGAACA-3′ for mouse *PRKAA2*. The constructs were then subjected to lentivirus packaging using HEK293T cells that were transfected with 1.5 µg of DNA in Lipofectamine 2000 transfection reagent per well of a 6-well plate. At 30 h after transfection, the virus (DMEM supplemented with 10% FBS, MEM non-essential amino acids, and sodium pyruvate; ~2 mL) was collected and centrifuged at 5000× *g* for 3 min at room temperature. The supernatant was mixed with 10 μg/mL (final concentration) polybrene; the virus mixture was then added to Hepa1-6 cells at 15% confluence, followed by centrifugation at 3000× *g* for 30 min at room temperature (spinfection). Cells were then incubated with the virus for another 48 h before the medium was refreshed. Cells at near confluence were single-cell-sorted into 96-well dishes. The resultant clones were expanded and evaluated for knockout status by sequencing.

### Primary mouse hepatocytes and primary HCC cells

Primary mouse hepatocytes were isolated with a modified two-step perfusion method using Liver Perfusion Medium and Liver Digest Medium as described previously.^30^ In brief, before isolation of hepatocytes, mice were first anaesthetized, followed by insertion of a 0.72 mm × 19 mm intravenous catheter into the postcava. After cutting off the portal vein, the mice were perfused with 50 mL of Liver Perfusion Medium at a rate of 5 mL/min, followed by 50 mL of Liver Digest Medium at a rate of 2.5 mL/min. The digested liver was briefly rinsed with PBS, and then dismembered by gently tearing apart the Glisson’s capsule with two sterilized, needle-pointed tweezers on a 6-cm dish containing 3 mL of PBS. The dispersed cells were mixed with 10 mL of ice-cold William’s Medium E plus 10% FBS and filtered by passing through a 100-μm cell strainer (Cat# 352360, BD Falcon). Cells were then centrifuged at 50× *g* at 4 °C for 2 min, followed by washing twice with 10 mL of ice-cold William’s Medium E plus 10% FBS. Cells were then immediately plated (at 60%–70% confluence) in collagen-coated six-well plates in William’s Medium E plus 10% FBS, GlutaMAX, 100 IU penicillin, and 0.1 mg/mL streptomycin, and maintained at 37 °C in a humidified incubator containing 5% CO_2_. After 2 h of attachment, the medium was replaced with fresh William’s Medium E with 1% (w/v) BSA and GlutaMAX for another 12 h before further use.

Mouse primary HCC cells were isolated from DEN/CCl_4_-induced HCC tissues or from Hepa1-6-derived allografts (generated as described previously,^124^ with minor modifications^108^). Cells were cultured in vitro as described previously.^125,126^ In brief, for HCC cells from DEN-HFD HCC, C57BL/6 J mice at 4 and 5 weeks of age were intraperitoneally injected twice with 80 mg/kg DEN, followed by intraperitoneal injection with 10% (v/v in corn oil) CCl_4_ at a dose of 5 mL/kg every week for 14 weeks from 8 weeks of age. The DEN/CCl_4_-induced HCC tissues were collected from 40-week-old mice. Tumor tissues were sliced into cubes with a diameter of 1–2 mm in serum-free DMEM in an ice bath, washed with ice-cold serum-free DMEM twice, and then intraperitoneally implanted into 8-week-old C57BL/6 J mice. After 1 month, the tumor tissues were collected. For HCC cells from Hepa1-6-derived allografts, the allograft-bearing mice were established as described in the “HCC-HFD, *MYC;Trp53*^−/−^ mice and HCC orthotopic allografts” section. Aldometanib was administered starting on day 2 post transplantation of Hepa1-6 cells (as described in Fig. 1f), and tumor tissues were collected on days 8, 14, and 17 post transplantation of Hepa1-6 cells. Tumor tissues were minced and digested with 10 mL of type I collagenase (1 mg/mL final concentration, in serum-free DMEM) in a Petri dish at 37 °C for 10 min. The cells were then centrifuged at 400× *g* for 5 min at room temperature, resuspended, and cultured in DMEM medium supplemented with 10% FBS and 10 µM Y-27632 for 24 h. The medium was refreshed to DMEM medium supplemented with 10% FBS every two days. Cells were ready for further use after at least 3 passages of culture.

### Reagents

Rabbit anti-phospho-AMPKα-Thr172 (Cat# 2535, RRID: AB_331250; 1:1000 dilution for immunoblotting (IB)), anti-AMPKα (Cat# 2532, RRID: AB_330331; 1:1000 for IB), anti-phospho-ACC-Ser79 (Cat# 3661, RRID: AB_330337; 1:1000 for IB), anti-ACC (Cat# 3662, RRID: AB_2219400; 1:1000 for IB), anti-phospho-p70 S6K-Thr389 (Cat# 9234, RRID: AB_2269803; 1:1000 for IB), anti-p70 S6K (Cat# 2708, RRID: AB_390722; 1:1000 for IB), anti-PUMA (Cat# 98672, RRID: AB_3096180; 1:1000 for IB), anti-caspase-3 (Cat# 14220, RRID: AB_2798429; 1:1000 for IB), anti-caspase-7 (Cat# 12827, RRID: AB_2687912; 1:1000 for IB), anti-GSDMD (Cat# 39754, RRID: AB_2916333; 1:1000 for IB), anti-Ki-67 (Cat# 12202, RRID: AB_2620142; 1:200 for IHC staining), anti-mTOR (Cat# 2972, RRID: AB_330978; 1:1000 for IB), anti-TCF1/TCF7-Alexa Fluor (Cat# 6709, RRID: AB_2797631; 1:100 for flow cytometry (F)), and mouse anti-caspase-9 (Cat# 9508, RRID: AB_2068620; 1:1000 for IB) antibodies were purchased from Cell Signaling Technology. Rabbit anti-tubulin (Cat# 10068-1-AP, RRID: AB_2303998; 1:1000 for IB), anti-GSDME (Cat# 13075-1-AP, RRID: AB_2093053; 1:1000 for IB), anti-AFP (Cat# 14550-1-AP, RRID: AB_2223933; 1:100 for IHC), anti-CXCL9 (Cat# 22355-1-AP, RRID: AB_2879086; 1:500 for IHC), anti-ALDOA (Cat# 11217-1-AP, RRID: AB_2224626; 1:5000 for IB), anti-ALDOB (Cat# 18065-1-AP, RRID: AB_2273968; 1:2000 for IB), and anti-CXCL10 (Cat# 10937-1-AP, RRID: AB_2088002; 1:200 for IHC) antibodies were purchased from Proteintech. Rabbit anti-CXCL11 (Cat# orb13425, RRID: AB_10749546; 1:100 for IHC) was purchased from Biorbyt. Rat anti-CD16/CD32 (Cat# 14-0161-86, RRID: AB_467135; 1:100 for F), anti-CD3 (Cat# 16-0032-85, RRID: AB_468852), syrian hamster anti-CD28 (Cat# 16-0281-85, RRID: AB_468922), and mouse anti-CD3 eFluor 506 (Cat# 69-0032-82, RRID: AB_2637489; 1:100 for F) antibodies were purchased from Invitrogen. Rat anti-IFNγ PE-CF594 (Cat# 562303, RRID: AB_11153140; 1:200 for F) antibody was purchased from BD Biosciences. Rat anti-CD45 Alexa Fluor 488 (Cat# 53-0451-82, RRID: AB_2848416; 1:200 for F), anti-CD45 PerCP-Cyanine5.5 (PerCP-Cy5.5) (Cat# 45-0451-82, RRID: AB_1107002; 1:200 for F), anti-CD8a APC (Cat# 17-0081-82, RRID: AB_469335; 1:200 for F), anti-CD11b APC (Cat# 17-0112-83, RRID: AB_469344; 1:200 for F), anti-CD11c PerCP-Cy5.5 (Cat# 45-0114-82, RRID: AB_925727; 1:200 for F), anti-granzyme B PE (Cat# 12-8898-82, RRID: AB_10870787; 1:200 for F), anti-IL-1β PE (Cat# 12-7114-82, RRID: AB_10732630; 1:200 for F), anti-IL-4 PE (Cat# 12-7041-82, RRID: AB_466156; 1:200 for F), anti-IL-17a PE (Cat# 12-7177-81, RRID: AB_763582; 1:200 for F), and mouse anti-NK1.1 APC (Cat# 17-5941-82, RRID: AB_469479; 1:200 for F) antibodies were purchased from eBioscience. Rat anti-CD3 PE (Cat# 100206, RRID: AB_312663; 1:200 for F), anti-CD4 Alexa Fluor 488 (Cat# 100529, RRID: AB_389303; 1:200 for F), anti-CD8a Alexa Fluor 488 (Cat# 100723, RRID: AB_389304; 1:200 for F), anti-F4/80 PE (Cat# 123110, RRID: AB_893486; 1:200 for F), anti-Ly6G-1A8 Alexa Fluor 488 (Cat# 127626, RRID: AB_2561339; 1:200 for F), anti-CD4 APC (Cat# 116014, RRID: AB_2563024; 1:200 for F), anti-CD19 PerCP-Cy5.5 (Cat# 152406, RRID: AB_2629815; 1:200 for F), anti-CD206 Alexa Fluor 488 (Cat# 141710, RRID: AB_10900445; 1:200 for F), anti-MHC II PE (Cat# 107608, RRID: AB_313323; 1:200 for F), anti-CD170-Siglec-F PE (Cat# 155506, RRID: AB_2750234; 1:200 for F), CCR3-APC (Cat# 144511, RRID: AB_2565737; 1:200 for F), anti-FOXP3 PE (Cat# 126404, RRID: AB_1089117; 1:100 for F), anti-CD366 (Tim-3) PerCP-Cy5.5 (Cat# 134012, RRID: AB_2632736; 1:100 for F), anti-CD62L APC (Cat# 104412, RRID: AB_313099, 1:100 for F), anti-CD44 PE (Cat# 103008, RRID: AB_312959; 1:100 for F), IgG2b PE, κ Isotype Ctrl Antibody (Cat# 400636, RRID: AB_893669; 1:100 for F), anti-CD279 (PD-1) PE Cy7 (Cat# 135216, RRID: AB_10689635; 1:100 for F), Armenian hamster anti-TCRγ/δ APC (Cat# 118116, RRID: AB_1731813; 1:200 for F), and anti-FcεRIα Alexa Fluor 488 (Cat# 134330, RRID: AB_2687239; 1:200 for F) antibodies were purchased from BioLegend. The neutralizing antibody for CD8^+^ T cells (clone 2.43; Cat# BE0061, RRID: AB_1125541), the neutralizing antibody for CD4^+^ T cells (clone GK1.5; Cat# BP0003-1, RRID: AB_1107636) and its isotype control antibody (rat IgG2b isotype control; clone LTF-2; Cat# BE0090, RRID: AB_1107780), and the neutralizing antibody for PD-1 (clone RMP1-14; Cat# BE0146, RRID: AB_10949053) and its isotype control antibody (rat IgG2a isotype control; clone 2A3; Cat# BE0089, RRID: AB_1107769) were purchased from BioXCell. Rat anti-CD8 (Cat# ab217344, RRID: AB_2890649; 1:2000 for IHC of mouse CD8a) and its isotype control (Cat# ab172730, RRID: AB_2687931; 1:2000 for IHC), anti-NK1.1 (Cat# ab289542, RRID: AB_3094493; 1:100 for IHC), rabbit anti-CD8 (Cat# ab245118, RRID: AB_3068617; 1:2000 for IHC of human CD8a), anti-CD4 antibody (Cat# ab288724, RRID: AB_2941893; 1:100 for IHC), and mouse anti-Ly6G (Cat# ab210204; 1:100 for IHC) were purchased from Abcam. The horseradish peroxidase (HRP)-conjugated goat anti-mouse IgG (Cat# 115-035-003, RRID: AB_10015289; 1:5000 for IB) and goat anti-rabbit IgG (Cat# 111-035-003, RRID: AB_2313567; 1:5000 for IB) antibodies were purchased from Jackson ImmunoResearch. Mouse anti-aldolase C (ALDOC; Cat# sc-271593, RRID: AB_10659113; 1:1000 for IB) was purchased from Santa Cruz Biotechnology.

Aldometanib was synthesized as described previously,^38^ and is now available at MedChemExpress (Cat# HY-148189), GLPBIO (Cat# GC66024), and CymitQuimica (Cat# TM-T60122). DMSO (Cat# D2650), methanol (Cat# 646377), ethanol (Cat# 459836), isopropanol (Cat# 34863), chloroform (Cat# C7559), NaCl (Cat# S7653), H_2_O_2_ (Cat# H1009), SDS (Cat# 436143), paraformaldehyde (Cat# 158127), glycine (Cat# G8898), penicillin G (Cat# P7794), streptomycin (Cat# S9137), sucrose (Cat# S7903), PBS (Cat# P5493), xylene (Cat# 534056), Tween-20 (Cat# P9416), Trizma base (Tris; Cat# T1503), polybrene (Cat# H9268), ammonium persulfate (APS; Cat# A3678), tetramethylethylenediamine (TEMED; Cat# T9281), diethylpyrocarbonate (DEPC)-treated water (Cat# 693520), ammonium hydroxide solution (Cat# 338818), hydrochloric acid in ethanol (Cat# 1.00327), trypsin (Cat# T1426), non-fat dried milk bovine (Cat# M7409), collagenase B (Cat# 11088831001), type I collagenase (Cat# C0130), Y-27632 (Cat# Y0503), (N-methyl-d3)-palmitoyl-L-carnitine (d_3_-L-carnitine; Cat# 55107), BSA (Cat# A2153), N-nitrosodiethylamine (DEN; Cat# N0756), Kolliphor HS 15 (Cat# 42966), Percoll (Cat# GE17-0891-09), Ficoll-Paque Premium (Ficoll; Cat# GE17-5442-03), LPS (Cat# L2630), and corn oil (Cat# C8267) were purchased from Sigma. ReverTra Ace qPCR RT Master Mix with gDNA Remover (Cat# FSQ-301) was purchased from Toyobo. The SignalStain DAB Substrate Kit (Cat# 8059) was purchased from Cell Signaling Technology. LabAssay triglyceride reagent (Cat# 290-63701) was purchased from Wako Pure Chemical Industries. WesternBright ECL and Peroxide solutions (Cat# 210414-73) were purchased from Advansta. Acrylamide/Bis Solution (30%), 29:1 (Cat# 1610156) was purchased from Bio-Rad. Lipofectamine 2000 (Cat# 11668500), GlutaMAX (Cat# 35050061), MEM non-essential amino acids solution (Cat# 11140050), sodium pyruvate (Cat# 11360070), Maxima SYBR Green/ROX qPCR Master Mix (Cat# K0223), FBS (Cat# 10099141 C), William’s E medium, no glutamine (Cat# 12551032), DMEM-high glucose (Cat# 12800082), DMEM/F-12 (Cat# 11320033), RPMI 1640 (Cat# 11875093), Liver Perfusion Medium (Cat# 17701), Liver Digest Medium (Cat# 17703), Prestained Protein MW Marker (Cat# 26612), and TRIzol (Cat# 15596018) were purchased from Thermo Scientific. Cytofix/Cytoperm Fixation/Permeabilization Kit (Cat# 554714) was purchased from BD Biosciences. The ALT assay kit (Cat# C009-2-1) and AST assay kit (Cat# C010-2-1) were purchased from Nanjing Jiancheng Bioengineering Institute. Paraplast High Melt Paraffin (Cat# 39601095) was purchased from Leica. [U-^13^C]-glutamine (Cat# 184161-19-1) was purchased from Cambridge Isotope Laboratories. CCl_4_ (Cat# C805329) was purchased from Macklin. Annexin V Apoptosis Detection Kits (Cat# 88-8007-74) were purchased from eBioscience. BsmBI (Cat# R0580L), the NEBNext Poly(A) mRNA Magnetic Isolation Module (Cat# E7490L), and the NEBNext Ultra RNA Library Prep Kit for Illumina (Cat# E7770L) were purchased from New England Biolabs. Phosphatase Inhibitor Cocktail I (Cat# HY-K0021), Phosphatase Inhibitor Cocktail II (Cat# HY-K0022), Protease Inhibitor Cocktail (Cat# HY-K0010), and DAPI (Cat# HY-D0814) were purchased from MedChemExpress. Hematoxylin solution (Cat# BSBA-4021A) and eosin Y solution (Cat# ZLI-9613) were purchased from ZSGB-BIO, China. Red Cell Lysis Buffer (Cat# RT122) was purchased from Tiangen. The TUNEL Apoptosis Detection Kit (Cat# 40307ES60) and 0.4% trypan blue solution (Cat# 40207ES20) were purchased from Yeason. Oxaliplatin (Cat# O124003) was purchased from Aladdin.

### Quantification of chemokine mRNA levels by real-time PCR

Experiments were performed as described previously,^127,128^ with minor modifications. Mice were killed by cervical dislocation, immediately followed by dissection of the tumor and para-carcinoma tissues. Total RNA was then prepared by lysing the tissue in 1 mL of TRIzol, followed by addition of 270 µL of chloroform and vigorous mixing. After centrifugation at 12,000× *g* for 15 min at 4 °C, 450 µL of the upper aqueous layer was transferred to a clean tube. The RNA was precipitated by adding 675 µL of isopropanol, followed by centrifugation at 12,000× *g* for 15 min at 4 °C. The pellet was washed with 75% ethanol 3 times by centrifugation at 12,000× *g* for 5 min, and then dissolved in 200 µL of DEPC-treated water. The RNA concentration was determined using a NanoDrop 2000 spectrophotometer (Thermo Scientific). A total of 2 µg of RNA was diluted with DEPC-treated water to a final volume of 4 µL, heated at 65 °C for 5 min, and then immediately chilled on ice. Random Primer Mix, Enzyme Mix, and 5× RT buffer (all from the ReverTra Ace qPCR RT kit) were added to the RNA solution, followed by incubation at 37 °C for 15 min and then at 98 °C for 5 min on a thermocycler. The reverse-transcribed cDNA was quantified with Maxima SYBR Green/ROX qPCR Master Mix on a LightCycler 480 system (Roche) with the following programme: pre-denaturation at 95 °C for 10 min; denaturation at 95 °C for 10 s, followed by annealing and extension at 60 °C for 30 s in each cycle; total cycles: 40. Primer pairs were designed using the PrimerBank database (https://pga.mgh.harvard.edu/primerbank/), and their sequences were: mouse *Actb*, 5′-GGCTGTATTCCCCTCCATCG-3′ and 5′-CCAGTTGGTAACAATGCCATGT-3′; mouse *Cxcl9*, 5′-TCCTTTTGGGCATCATCTTCC-3′ and 5′-TTTGTAGTGGATCGTGCCTCG-3′; mouse *Cxcl10*, 5′-CCAAGTGCTGCCGTCATTTTC-3′ and 5′-GGCTCGCAGGGATGATTTCAA-3′; and mouse *Cxcl11*, 5′-GGCTTCCTTATGTTCAAACAGGG-3′ and 5′-GCCGTTACTCGGGTAAATTACA-3′. Data were analyzed using LightCycler 96 software (v1.1, Roche). The mRNA level relative to that of beta-actin (*Actb*) was calculated using Excel software (2016, Microsoft).

### RNA sequencing

Levels of chemokine gene expression in tumor and para-tumor tissues were determined through RNA-sequencing performed by Tissuebank Biotechnology Co., Ltd. (Shanghai, China). In brief, 50 mg of tumor and para-tumor tissue dissected by freeze-clamping were instantly lysed in 1 mL of TRIzol, and then centrifuged at 12,000× *g* for 15 min at 4 °C. Approximately 900 μL of supernatant (without the lipid layer) was transferred to an RNase-free tube and mixed with 270 μL of chloroform. After vigorous vortexing for 15 s, the mixture was centrifuged at 12,000× *g* for 15 min at 4 °C, and ~450 μL of the upper aqueous layer was transferred to an RNase-free tube. The RNA was precipitated by addition of 675 μL of isopropanol, followed by centrifugation at 12,000× *g* for 30 min at 4 °C. The pellet was washed twice with 75% ethanol (v/v, in DEPC-treated water) and dissolved in 200 μL of DEPC-treated water. The RNA concentration was determined using a NanoDrop 2000 spectrophotometer (Thermo), and RNA integrity was assessed with a Fragment Analyzer 5400 (Agilent Technologies).

cDNA libraries were generated using the NEBNext Poly(A) mRNA Magnetic Isolation Module and the NEBNext Ultra RNA Library Prep Kit for Illumina following the manufacturer’s instructions. In brief, mRNA from each sample was captured using NEBNext Oligo d(T)_25_ beads, and then fragmented in First Strand Synthesis Reaction Buffer at 94 °C for 15 min. The fragmented mRNA was used to synthesize first-strand cDNA with random hexamer primers and M-MuLV Reverse Transcriptase (M-MLV), followed by second-strand cDNA with DNA Polymerase I and RNase H. The remaining cDNA overhangs were converted into blunt ends, followed by adenylation of the 3′ ends using the NEBNext End Prep Enzyme Mix and ligation to NEBNext Adaptors. The ligation products were purified using AMPure XP Beads (Beckman Coulter), during which cDNA fragments of 250–300 bp were enriched, followed by treatment with the USER Enzyme at 37°C for 15 min to remove hairpin structures on the adaptors. After incubation at 95 °C for 15 min, the adaptor-ligated cDNAs were amplified by PCR using Phusion High-Fidelity DNA Polymerase, Universal PCR Primers, and the Index Primer, and then purified with AMPure XP Beads. The quality of the resulting library was assessed on a Bioanalyzer 2100 (Agilent Technologies). The amplified library was clustered using the TruSeq PE Cluster Kit v3-cBot-HS kit (Illumina) on a cBot Cluster Generation System (Illumina) and sequenced on the NovaSeq 6000 platform (Illumina) to generate 150-bp paired-end reads.

The original image files generated during sequencing were transformed to short reads (raw data, in FASTQ format) by base calling. Low-quality sequences (Phred quality score < 5), sequences with adaptor contamination (detected in either read of a read pair), and unrecognizable sequences (over 10% of bases unrecognizable in either read of a read pair) were removed from the raw reads using fastp software (v0.21.1; ref. ^129^). Gene expression levels were quantified as FPKM (fragments per kilobase of gene per million mapped reads) values. To obtain the FPKM value of each gene, reads were first mapped to the mouse reference sequence using HISAT2 software (v2.2.1) as described previously^130^ to ensure that the reads could be uniquely mapped to the gene chosen for FPKM calculation. For genes with more than one alternative transcript, the longest transcript was selected for FPKM calculation. FPKM values were calculated using featureCounts software (v2.0.1) as described previously.^131^ FPKM values for *Cxcl9*, *Cxcl10*, and *Cxcl11* were plotted using Prism 9 software (GraphPad).

### Determination of aldometanib concentrations in mouse serum and tissues

The concentrations of aldometanib in the livers and serum of DEN-HFD mice were determined using the protocol described previously,^38^ with minor modifications. In brief, after intraperitoneal injection with two doses of DEN on postnatal days 28 and 35 and feeding with HFD starting from day 49, C57BL/6J mice were administered 100 mg/L aldometanib in the drinking water for 1 week starting after 33 weeks of HFD treatment. At the end of the aldometanib treatment, liver tissues and blood were collected. The blood was left at room temperature for 20 min and then centrifuged at 3000× *g* for 30 min at 4 °C to obtain the serum. A total of 100 µL of serum or 100 mg of liver or tumor tissue was lysed with 1 mL of ice-cold 80% (v/v) methanol in water containing 2.3 ng/mL d_3_-L-carnitine C16:0 as an internal standard, followed by centrifugation at 20,000× *g* for 15 min at 4 °C. Approximately 600 µL of the supernatant was collected, lyophilized in a vacuum concentrator (CentriVap Benchtop Centrifugal Vacuum Concentrator equipped with a CentriVap −84 °C Cold Trap and a Scroll Vacuum Pump, Labconco) at 4 °C, and then dissolved in 100 µL of 70% (v/v, in water) methanol. Samples were analyzed on a QTRAP MS (QTRAP 6500 + , SCIEX) interfaced with a UPLC system (Acquity I-class, Waters). A total of 2 µL of each sample was loaded onto a reverse-phase column (ACQUITY UPLC BEH C18, 1.7 μm, 2.1 mm × 50 mm; Cat# 186002350, Waters). The mobile phase consisting of 0.1% formic acid in LC-MS-grade water (mobile phase A) and LC-MS-grade methanol (mobile phase B) was run at a flow rate of 0.2 mL/min. The analytes were separated with the following gradient program: 70% B increased to 100% B in 5 min, held for 2 min, with the post time set to 3 min. The QTRAP mass spectrometer used a Turbo V ion source and ran in positive mode with a spray voltage of 5500 V, source temperature of 400 °C, gas 1 at 40 psi, gas 2 at 50 psi, and curtain gas at 40 psi. Aldometanib was measured using the multiple reaction monitoring (MRM) mode, and declustering potentials and collision energies were optimized through the use of analytical standards. The following transitions were used to monitor each compound: 465.3/240.6 and 465.3/159.1 for aldometanib and 403.4/85 for d_3_-L-carnitine C16:0 as an internal standard. Data were collected using Analyst software (v1.6.3, SCIEX), and the relative amounts of aldometanib were analyzed using MultiQuant software (v3.0.2, SCIEX).

### Immunoblotting

Immunoblotting was performed as described previously.^132,133^ In brief, to analyze the levels of p-AMPKα, p-ACC, p-S6K, and the protein levels of cell death markers in cultured cells, cells were grown to 95% confluence in wells of a 6-well dish, and then lysed with 300 μL of ice-cold lysis buffer in each well. To analyze the levels of apoptotic markers in HCC tissues, 100 mg of freshly excised tissue was lysed with ice-cold lysis buffer (10 μL/mg liver weight), and homogenized with a T 10 basic ULTRA-TURRAX hand-held homogenizer equipped with an S10N-5G dispersing tool (IKA). The lysates were sonicated using a sonicator (VCX130PB, SONICS) equipped with a 5/64′′ (2-mm) stepped microtip (Cat# 630-0423, SONICS) for 3 s at 25% maximum power on ice, then centrifuged at 20,000× *g* for 10 min at 4 °C. The pellet was discarded, and an equal volume of 2× SDS sample buffer was added to the supernatant. Samples were then boiled for 10 min before gel electrophoresis and immunoblotting.

All protein samples were subjected to immunoblotting on the same day of preparation without any freeze-thaw cycles.

For immunoblotting, SDS-PAGE gels were prepared in-house as described previously.^36^ In brief, the resolving gel solution (8%, 10 mL) was prepared by mixing 1.9 mL of 30% Acryl/Bis solution, 1 mL of 10× Lower Buffer (3.5 M Tris, 1% (w/v) SDS, pH 8.8), and 0.48 mL of 65% (w/v) sucrose (dissolved in water) with 6.62 mL of water; the stacking gel solution (5 mL) was prepared by mixing 668 μL of 30% Acryl/Bis solution and 1.25 mL of 4× Stacking Buffer (0.5 M Tris, 0.4% (w/v) SDS, pH 6.8) with 3.08 mL of water. For each glass gel plate (with 1.0-mm spacer; Cat# 1653308 and 1653311, Bio-Rad), ~7 mL of resolving gel solution and 2.5 mL of stacking gel solution were required. APS (to 0.1% (w/v) final concentration) and TEMED (to 0.1% (v/v) final concentration) were added to the resolving gel solution. The resolving gel was overlaid with 2 mL of 75% (v/v) ethanol before acrylamide polymerization. After ~20 min (when a clear line was visible between the resolving gel and the ethanol), the overlaid ethanol was poured off, and the gel was dried with filter paper and placed at room temperature for another 15 min to let the ethanol evaporate completely. The gel cassette was then filled with APS/TEMED-supplemented stacking gel solution, a 15-well comb was placed into the cassette, and the gel was kept at room temperature for 20 min. After removal of the comb, the gel was rinsed with Running Buffer (25 mM Tris, 192 mM glycine, 1% (w/v) SDS, pH 8.3) before sample loading. Samples of less than 10 μL were loaded into wells, and electrophoresis was run at 100 V in a Mini-PROTEAN Tetra Electrophoresis Cell (Bio-Rad). All samples were resolved on 8% resolving PAGE gels, except those for caspase-3, caspase-7, GSDME, GSDMD, and PUMA, which were run on 12% gels (prepared like the 8% gels, except that a final concentration of 12% Acryl/Bis was added to the resolving gel solution). The resolved proteins were electrically transferred to pre-cut PVDF membranes (0.45 μm; IPVH00010, Merck), which were pre-incubated in methanol for 1 min, then equilibrated and soaked in pre-cooled Transfer Buffer (25 mM Tris, 192 mM glycine, 10% (v/v) methanol) for more than 5 min. After preparing the gel/membrane sandwich, the transfer was performed at a voltage of 100 V in a Mini Trans-Blot Cell (Bio-Rad) for 1 h at 4 °C. The blotted PVDF membrane was then incubated in blocking buffer (5% (w/v) BSA or 5% (w/v) non-fat milk (according to the instructions from the antibody suppliers)) dissolved in TBST (40 mM Tris, 275 mM NaCl, 0.2% (v/v) Tween-20, pH 7.6) for another 2 h on an orbital shaker at room temperature, and then rinsed with TBST twice for 5 min each. The PVDF membrane was incubated with the desired primary antibody overnight at 4 °C on an orbital shaker with gentle shaking, rinsed with TBST three times for 5 min each at room temperature, and then incubated with the secondary antibody for 3 h at room temperature with gentle shaking. The secondary antibody was then removed, and the PVDF membrane was washed with TBST three times for 5 min each at room temperature. PVDF membranes were incubated in ECL mixture (by mixing equal volumes of ECL solution and peroxide solution for 5 min), then placed in plastic wrap and covered with Medical X-Ray Film (FUJIFILM) in a light-proof cassette for a desired period of time. The films were developed with X-OMAT MX Developer and Replenisher and X-OMAT MX Fixer and Replenisher solutions (Carestream) on a Medical X-Ray Processor (Carestream) using Developer (Model 002, Carestream). The developed films were scanned using a Perfection V850 Pro scanner (Epson) with Epson Scan software (v3.9.3.4, Epson) and cropped using Photoshop (2023, Adobe). Levels of total proteins and phosphorylated proteins were analyzed on separate gels, and representative immunoblots are shown. The band intensities on the developed films were quantified using ImageJ (v1.8.0, National Institutes of Health freeware). Uncropped immunoblots are uploaded as a “Full scans” file.

### Measurement of adenylates

To analyze ATP, ADP, and AMP in tissues, HPLC-MS was performed as described previously.^38,134^ In brief, 100 mg of liver tissue dissected by freeze clamping was instantly lysed in 1 mL of methanol, then mixed with 1 mL of chloroform and 400 µL of water (containing 4 µg/mL [U-^13^C]-glutamine), followed by 20 s of vortexing. After centrifugation at 15,000× *g* for 15 min at 4 °C, 800 µL of the aqueous phase was collected, lyophilized in a vacuum concentrator at 4 °C, and dissolved in 30 µL of 50% acetonitrile (v/v) in water. AMP and ATP levels were measured as described in ref. ^135^ using a QTRAP MS (SCIEX, QTRAP 5500) interfaced with a UPLC system (SCIEX, ExionLC AD). A total of 2 µL of each sample was loaded onto an HILIC column (ZIC-pHILIC, 5 μm, 2.1 mm × 100 mm, PN: 1.50462.0001, Millipore). The mobile phase consisted of 15 mM ammonium acetate containing 3 mL/L ammonium hydroxide ( > 28%, v/v) in LC-MS-grade water (mobile phase A) and LC-MS-grade 90% (v/v) acetonitrile in LC-MS-grade water (mobile phase B) run at a flow rate of 0.2 mL/min. AMP, ADP, and ATP were separated using the following HPLC gradient elution program: 95% B held for 2 min, then to 45% B in 13 min, held for 3 min, and then back to 95% B for 4 min. The mass spectrometer was run on a Turbo V ion source in negative mode with a spray voltage of −4500 V, source temperature of 550 °C, gas no. 1 at 50 psi, gas no. 2 at 55 psi, and curtain gas at 40 psi. The following transitions were used to monitor each compound: 505.9/158.9 and 505.9/408.0 for ATP; 425.9/133.9, 425.9/158.8, and 425.9/328.0 for ADP; 345.9/79.9, 345.9/96.9, and 345.9/133.9 for AMP; and 149.9/114 for [U-^13^C]-glutamine. Data were collected using Analyst 1.7.1 software (SCIEX), and the relative amounts of metabolites were analyzed using MultiQuant 3.0.3 software (SCIEX). Note that a portion of ADP and ATP could lose one or two phosphate groups during in-source fragmentation, thus yielding the same m/z ratios as AMP and ADP; this was corrected according to their different retention times in the column.

### Determination of the composition of immune cells in HCC tissues

The composition of immune cells in HCC tissues was determined by a flow-cytometry-based method as described previously,^136,137^ with minor modifications. In brief, HCC-bearing mice were euthanized by cervical dislocation, and the HCC tissues were quickly excised without draining the blood. The tissues were then minced into 1-mm pieces using ophthalmic scissors, and ~100 mg of tissue was digested with 2 mL of type Ⅰ collagenase (1 mg/mL) dissolved in PBS supplemented with 10% FBS in a 5-mL conical tube for 60 min at 37 °C in a shaker at 50 rpm. The digestions were filtered using a 70-μm cell strainer, and the filtrate was centrifuged at 400× *g* for 5 min at 25 °C. The resulting cell pellets were gently mixed in 5 mL of 40% Percoll (prepared by mixing 2 mL of Percoll with 0.22 mL of 10× PBS and 2.78 mL of 1× PBS), then overlaid on top of a 3 mL of 80% Percoll cushion (consisting of 2.4 mL Percoll, 0.27 mL of 10× PBS, and 0.33 mL of 1× PBS) in a 15-mL conical tube and centrifuged for 20 min at 2000× *g* and 25 °C. Note that the centrifuge was set to minimum acceleration and the brake program deactivated, as this could disturb the Percoll gradient and, in turn, lower the cell yield. After centrifugation, the fraction located at the 40%–80% Percoll interface (~2 mL), which contained immune cells, was collected with a Pasteur pipet and mixed with 10 mL of PBS solution, and then centrifuged for 5 min at 400× *g* and 25 °C. A total of 1 × 10^6^ cells were incubated with anti-CD16/CD32 antibody (1:100 in PBS in a total volume of 50 µL) for 30 min at room temperature in a 96-well round-bottom plate (non-tissue culture treated, Cat# CLS7007-24EA, Corning). The desired combinations of primary antibodies, in a total volume of 50 µL as specified in the figure legends, were then added to the cells. After incubation for 30 min at room temperature, the cells were washed twice with 200 µL of PBS. In particular, before staining granzyme B, IFNγ, IL-1β, IL-4, and IL-17a, cells were treated with 200 µL of the fixation and permeabilization buffer included in the Cytofix/Cytoperm Fixation/Permeabilization Kit for 30 min at room temperature, and then washed once with 200 µL of washing buffer. Cells were incubated in 200 μL of 1% (v/v) formalin in the dark at 4 °C before the flow cytometry analysis.

Flow cytometry was performed using an LSRFortessa X20 cell analyzer (BD Biosciences), which was equipped with 5 solid-state lasers (355 nm, 15 mW; 405 nm, 50 mW; 488 nm, 50 mW; 561 nm, 30 mW; and 640 nm, 40 mW), a forward scatter (FSC) detector, a side scatter (SSC) detector, and an 18-channel fluorophore detector. The 488-nm laser and the 530/30 filter were used for excitation and detection of Alexa Fluor 488 fluorescence, the 488-nm laser and the 710/50 filter for PerCP-Cy5.5, the 561-nm laser and the 586/15 filter for PE, and the 640-nm laser and the 670/14 filter for APC. Detector voltages were optimized using a modified voltage titration approach.^138^ Gating strategies used to identify each type of immune cells during the analysis are shown in the corresponding panels. Gate boundaries were either set based on control samples or followed density distributions based on best practices. Data were collected with FACSDiva software (v8.0.2, BD Biosciences), and then exported in FCS 3.1 format. The numbers of each type of immune cells were quantified with FlowJo software (v10.4.0, BD Biosciences). During the analysis, a combination of manual gating and computational analysis approaches^139^ was used.

### Determination of the composition of immune cells in the thymus and spleen

The composition of immune cells in the thymus and spleen was determined by flow cytometry as described previously,^140^ with minor modifications. In brief, mice bearing Hepa1-6 allografts were sacrificed through cervical dislocation, and the thymus and spleen were carefully collected. The tissues were placed on a 70-μm cell strainer (Cat# 352350, BD Falcon) and gently dispersed using a syringe plunger in pre-cooled PBS until fully disrupted. Approximately 5 mL of PBS was added to rinse and filter the cells from the cell mixture. Approximately 1 × 10^6^ cells were then incubated with anti-CD16/CD32 blocking antibody (1:100 in PBS in a total volume of 50 µL) for 30 min at room temperature in a 96-well round-bottom plate (non-tissue culture treated, Cat# CLS7007-24EA, Corning). The desired combinations of primary antibodies (1:100 in PBS), in a total volume of 50 µL as specified in the figure legends, were then added to the cells and incubated for an additional 30 min at room temperature. After two washes with 200 µL of PBS, the cells were incubated in 200 μL of 1% (v/v) formalin in the dark at 4 °C before flow cytometry analysis.

### Histology

For H&E staining, liver tissues excised from blood-drained mice were cut into pieces, fixed in 4% (v/v) paraformaldehyde within 48 h at room temperature, and transferred to embedding cassettes. The cassettes were washed in running water for 12 h, then successively soaked in 70% ethanol (v/v in water), 80% ethanol, and 95% ethanol for 1 h each. The fixed tissues were further dehydrated in anhydrous ethanol for 1 h twice, followed by immersion in 50% xylene (v/v in ethanol) for 30 min, two changes of xylene for 15 min each, and two changes of paraffin wax (58–60 °C) for 1 h each. The dehydrated tissues were embedded in paraffin on a HistoCore Arcadia Paraffin Embedding Machine (Leica). Paraffin blocks were sectioned to a thickness of 4 μm, dried on an adhesion microscope slide, and rehydrated in the following order: two changes of xylene at 70 °C for 10 min each; two changes of anhydrous ethanol for 5 min each; two changes of 95% ethanol for 5 min each; one change each of 80% ethanol, 70% ethanol, and 50% ethanol for 5 min each; and a final brief immersion in water. The sections were then stained in hematoxylin solution for 8 min, washed in running water for 5 min, differentiated in 1% hydrochloric acid (in ethanol) for 30 s, washed in running water for 1 min, immersed in 0.2% (v/v in water) ammonium hydroxide solution for 30 s, washed in running water for 1 min, and stained in eosin Y solution for 30 s. The stained sections were dehydrated in 70% ethanol for 5 min, twice in 95% ethanol for 5 min each, twice in anhydrous ethanol for 5 min each, and twice in xylene for 15 min each. The stained sections were mounted with Canada balsam and visualized using a Zeiss Observer Z1 microscope (Carl Zeiss, Jena, Germany).

For IHC, liver tissues were fixed, dehydrated, embedded, sectioned, and re-hydrated as in H&E staining and washed with water three times for 5 min each at room temperature. The sections were then incubated in pre-heated (~95 °C) citrate antigen retrieval buffer (1 mM sodium citrate, pH 6.0, 0.05% (v/v) Tween-20) for 1.5 min and cooled at room temperature for 60 min. The sections were washed with washing buffer (0.1% (v/v) Tween-20 in PBS) twice for 5 min each at room temperature, incubated in 10% (v/v) H_2_O_2_ (in methanol) for 5 min at room temperature, and washed with washing buffer three times for 5 min each at room temperature. The sections were then incubated in 1% (w/v) BSA (diluted with PBS) at room temperature for 20 min. After draining, the liver sections were circled with a PAP pen (Cat# Z377821, Sigma), incubated with primary antibodies (diluted in 1% BSA solution) at 4 °C in a dark, humidified chamber, and washed with washing buffer 3 times for 5 min each at room temperature. The sections were incubated with HRP-conjugated goat anti-rabbit IgG or goat anti-mouse IgG (diluted in 1% BSA solution) for 1 h at room temperature in a dark, humidified chamber, and then washed with washing buffer 3 times for 5 min each at room temperature. The sections were then incubated with DAB working solution, which was freshly prepared by adding 30 μL of SignalStain DAB Chromogen Concentrate to 1 mL of SignalStain DAB Diluent (both included in the SignalStain DAB Substrate Kit) and mixing well. The incubation lasted ~5 min in the dark at room temperature, until a good staining intensity developed, as examined on a Zeiss Observer Z1 microscope (Carl Zeiss, Jena, Germany). The sections were then washed with running water for 5 min, stained in hematoxylin solution for 8 min, and washed in running water for another 5 min. They were differentiated in 1% hydrochloric acid (in ethanol) for 30 s, washed in running water for 1 min, dehydrated in 70% ethanol for 5 min, washed twice in 95% ethanol for 5 min each, washed twice in anhydrous ethanol for 5 min each, and given two changes of xylene for 15 min each. The stained sections were mounted with Canada balsam and visualized using an Axioscan 7 microscope (Zeiss).

To measure hepatic TAG contents, mice were euthanized by cervical dislocation, and the livers were immediately excised and rinsed in PBS three times. Approximately 50 mg of tissue was homogenized with a hand-held homogenizer (T 10 basic ULTRA-TURRAX equipped with an S10N-5G dispersing tool, IKA) in 1 mL of PBS containing 5% (v/v) Triton X-100 and sonicated with a sonicator (VCX130PB equipped with a 5/64′′ stepped microtip, SONICS) at 30% maximum power for 20 cycles, 1 s per cycle with 2-s intervals at room temperature. Approximately 600 μL of the supernatant was centrifuged at 20,000× *g* for 10 min at 25 °C. The resulting supernatant was boiled for 20 min, and then centrifuged at 20,000× *g* at 25 °C for 10 min. Approximately 3 μL of the supernatant was used for each test performed with the LabAssay Triglyceride Kit according to the manufacturer’s instructions.

### Determination of serum ALT and AST levels

Serum samples were freshly prepared as described in the “HCC-HFD, *MYC*;*Trp53*^−/−^ mice and HCC orthotopic allografts” section. Levels of serum ALT and AST were determined using the ALT assay kit and AST assay kit following the manufacturer’s instructions. Approximately 5 μL of serum was used for each test, and the optical density (OD) was recorded with a SpectraMax M5 microplate reader (Molecular Devices) using SoftMax Pro software (v5.4.1.1, Molecular Devices).

### Determination of the rates of cell death

The effects of aldometanib on cell death in cultured cells were determined by a flow cytometry-based method as described previously,^108^ with minor modifications. In brief, HCC cells or normal liver cells grown to 70%–80% confluence in a 6-well dish were treated with aldometanib, washed with 1 mL of PBS (pre-heated to 37 °C), trypsinized with 0.25% trypsin, and centrifuged at 1200× *g* for 5 min at 4 °C. Approximately 1 × 10^6^ of trypsinized cells were washed with 0.2 mL of the Binding Buffer provided in the Annexin V Apoptosis Detection Kit. Cell suspensions were centrifuged at 1200× *g* for 5 min at 4 °C, resuspended in 200 μL of Binding Buffer, and stained with 5 μL of Annexin V-APC solution for 30 min at room temperature in the dark. Cell suspensions were incubated with 5 μL of PI solution for 5 min at room temperature in the dark, and then centrifuged at 1200× *g* for 5 min at 4 °C to remove dye that was not bound to cells. Cell suspensions were diluted with 400 μL of Binding Buffer and immediately subjected to flow cytometry analysis. Flow cytometry was performed on a BD LSRFortessa X20 cell analyzer, with the 640-nm (40 mW) laser and 670/14 filter used to excite and detect the fluorescence of Annexin V-APC, and the 561-nm (30 mW) laser and 585/42 filter for PI. Detector voltages were optimized using a modified voltage titration approach.^138^ Gating strategies used during the analysis were: (1) FSC-H and SSC-H for selecting intact cells and (2) FSC-H and FSC-A for excluding doublets. Gate boundaries were either set based on control samples or followed density distributions based on best practices. Data were collected with FACSDiva software (v8.0.2, BD Biosciences) and exported in FCS 3.1 format. The numbers of early apoptotic cells (Annexin V positive and PI negative populations) and late apoptotic and necroptotic cells (Annexin V positive and PI positive populations) were quantified with FlowJo software (v10.4.0, BD Biosciences). For this experiment, a combination of manual gating and computational analysis approaches^139^ was used.

The effects of aldometanib on cell death in HCC tissues were determined by a TUNEL assay using the TUNEL Apoptosis Detection Kit according to the manufacturer’s instructions, with minor modifications. In brief, liver paraffin sections were baked at 70 °C for 4 h, quickly immersed in two changes of xylene at room temperature for 5 min each, and then washed twice in anhydrous ethanol at room temperature for 5 min each. The sections were sequentially incubated in 90%, 80%, and 70% ethanol for 3 min each, and then briefly washed with PBS. After draining, the sections were circled with a PAP pen, incubated with 100 μL of proteinase K solution (20 μg/mL final concentration in PBS) at room temperature for 20 min, and rinsed with PBS for 5 min at room temperature. The sections were incubated with 100 μL of 1× Equilibration Buffer (freshly prepared by diluting 5× Equilibration Buffer in double-distilled water) at room temperature for 30 min, and then incubated in 100 μL of TdT Reaction Buffer (freshly prepared by mixing double-distilled water, 5× Equilibration Buffer, FITC-12-dUTP Labeling Mix, and Recombinant TdT Enzyme at a ratio of 34:10:5:1) in a dark, humidified chamber at 37 °C for 1 h. The sections were then rinsed with PBS for 5 min at room temperature, incubated in 100 μL of 0.1% (v/v) Triton X-100 solution (in PBS containing 5 mg/mL BSA) three times for 5 min each at room temperature, and rinsed with PBS for 5 min at room temperature. The sections were then incubated with freshly prepared DAPI solution (2 μg/mL, in PBS) for 5 min in the dark at room temperature and rinsed with double-distilled water three times for 5 min each at room temperature. The drained sections were mounted with Canada balsam and visualized using an Axioscan 7 microscope (Zeiss).

The effects of aldometanib on cell growth were determined by quantifying the numbers of viable cells at different time points during culture by trypan blue staining. Specifically, 2 × 10^5^ BNL cells, 1.5 × 10^5^ Huh7 cells, 1.5 × 10^5^ JHH-7 cells, 1 × 10^6^ mouse primary HCC cells, and 4 × 10^5^ Hepa1-6 cells were seeded into 6-well dishes. After 12 h of incubation, the cells were treated with aldometanib at the desired concentrations for 24 h, then rinsed with PBS and trypsinized. To determine the growth of Hepa1-6 cells derived from control or aldometanib-treated Hepa1-6 allografts, 2 × 10^5^ Hepa1-6 cells were seeded into 6-well dishes and cultured for 24 h before harvest by trypsinization. The trypsinized cells were collected by centrifugation at 2000× *g* for 5 min at room temperature, then resuspended in 500 μL of PBS. Approximately 100 μL of cell suspension was incubated with 100 μL of 0.4% trypan blue solution for 10 min at room temperature, and 20 μL of stained cells were then added to a Millicell Hemocytometer (Cat# MDH-2N1, Sigma) to quantify the number of living cells.

### Glucose tolerance test

Glucose tolerance of mice was assessed by the ipGTT as described previously,^36,127^ with minor modifications. In brief, DEN-HFD mice were individually caged for one week before the experiment. The mice were treated with aldometanib (100 mg/L) at 12 weeks of age (as depicted in the upper panel of Fig. 1a). At 40 weeks of age, mice were fasted for 12 h (10 p.m. to 10 a.m.), then administered glucose at 1 g/kg body weight via intraperitoneal injection. Blood glucose was measured at the indicated time points through tail-vein bleeding using the OneTouch UltraVue automatic glucometer (LifeScan).

### Determination of the cytotoxic effects of CD8^+^ T cells on Hepa1-6 cells in vitro

This experiment was performed as described previously,^141,142^ with minor modifications. In brief, wild-type C57BL/6 J mice were sacrificed by cervical dislocation, and the spleens were excised under sterile conditions. The tissues were placed on a 70-μm cell strainer (Cat# 352350, BD Falcon) and gently dispersed with a syringe plunger in pre-cooled PBS until completely disrupted. Approximately 5 mL of PBS was then added to rinse and filter the splenocytes from the cell mixture. After centrifugation at 400× *g* for 5 min, the cell pellet was resuspended in red blood cell lysis buffer at a ratio of 1 mL of buffer per mL of splenocytes. This mixture was pipetted and incubated at room temperature for 1 min. The lysate was then mixed with five times the volume of PBS and centrifuged again at 400× *g* for 5 min. The cell pellets were resuspended in RPMI-1640 medium containing 10% FBS, 1% GlutaMAX, and 1% penicillin/streptomycin, adjusting the concentration to 3 × 10^6^ cells/mL. Approximately 2 mL of the cell suspension was then incubated with anti-mouse CD3 antibody (final concentration of 2 µg/mL, diluted in PBS) in a 6-well plate and incubated at 37 °C in a 5% CO_2_ incubator for 2 h. After aspiration of the supernatant, cells were incubated with 2 mL of anti-mouse CD28 antibody (final concentration of 4 μg/mL, diluted in PBS) at 37 °C in a 5% CO_2_ incubator for 72 h to enable complete activation of the CD8^+^ T cells. Aldometanib was added 6 h after the initiation of CD8^+^ T cell activation.

To prepare the cytotoxicity assay, 8 × 10^4^ Hepa1-6 cells (target cells; T) were added to a well of a 24-well plate and incubated for 8 h, and then mixed with the activated CD8^+^ T cells (effector cells; E) at various effector-to-target ratios (E:T = 0:1 (control), 2:1, 5:1, and 10:1). The mixtures were then centrifuged at 400× *g* for 5 min, and the pellet was resuspended in RPMI-1640 complete medium. After 48 h of incubation at 37 °C in a 5% CO_2_ incubator, the medium was removed, and the wells were rinsed three times with PBS to thoroughly remove CD8^+^ T cells. Approximately 500 μL of a 0.1% crystal violet methanol solution (Cat# G1072, Solarbio, diluted with methanol) was added to each well to stain the attached remaining Hepa1-6 cells. The wells were then incubated at room temperature for 30 min and gently rinsed three times with deionized water, and 200 μL of 33% acetic acid was added to each well. The wells were gently shaken for 10 min, and the OD_570_ was recorded using a SpectraMax M5 microplate reader (Molecular Devices) with SoftMax Pro software (v5.4.1.1). The survival rate of the Hepa1-6 cells was calculated using the following formula: survival rate = OD value of co-culture wells/ΔOD value (where ΔOD is the average OD value of the control group, which contained only Hepa1-6 cells).

### Determination of the invasive and migration ability of HCC cells

Cell invasiveness was assessed using the Transwell assay as described previously.^143,144^ Prior to this assay, cell culture inserts with 8-μm pores (Transwell; Cat# CLS3422, Corning) were hydrated overnight. Each Transwell insert was then coated with 60 µL of Matrigel (1 mg/mL, Cat# 356230, Corning) using pre-cooled pipette tips, placed on ice, and incubated at 37 °C for 2 h. Approximately 200 µL of serum-free DMEM containing 2 × 10^5^ Hepa1-6 cells was then seeded into the coated Transwell inserts, which were subsequently placed into 24-well plates containing 500 µL of DMEM medium supplemented with 20% FBS and cultured for 24 h. After culturing, the Transwell inserts were retrieved and rinsed three times with PBS. The inserts were then gently dried with cotton swabs, fixed, and stained with a 0.1% crystal violet solution diluted in methanol (Cat# G1072, Solarbio) at room temperature for 30 min. After rinsing three additional times with PBS, the inserts were air-dried and observed under an inverted microscope. The number of stained cells was counted at 100× magnification, with five random areas selected from each Transwell insert, using ImageJ software to calculate the average of the five areas.

Cell migration was determined using the scratch assay, with minor modifications as described previously.^143^ In brief, Hepa1-6 cells were seeded into 6-well plates until they reached > 90% confluence. A straight wound was created in each well by scraping the cells vertically with a sterile 200-μL pipette tip. The cells were washed three times with PBS and then incubated with various concentrations of aldometanib (0 nM, 10 nM, 20 nM, 50 nM, 100 nM, or 200 nM) dissolved in serum-free DMEM. Images of the wounded area were taken at identical locations at 0 h and 24 h after treatment with aldometanib. The migration area was quantified using ImageJ software, and the relative wound closure at 24 h was calculated.

### Determination of glucose concentration in tumor and para-tumor tissues

The concentration of glucose in tumor and para-tumor tissues was determined using the Glucose (GO) Assay Kit (Cat# GAGO20, Sigma-Aldrich) following the manufacturer’s instructions, with minor modifications. In brief, 100 mg of freshly excised tumor and para-tumor tissues from Hepa1-6 allograft-bearing mice at day 17 post-transplantation were lysed in ice-cold deionized water (10 µL/mg tissue weight). The samples were then homogenized using a hand-held homogenizer (T 10 basic ULTRA-TURRAX) equipped with an S10N-5G dispersing tool (IKA). The lysates were sonicated using a sonicator (Cat# VCX130PB, SONICS) with a 5/64′′ (2-mm) stepped microtip (Cat# 630-0423, SONICS) for 30 s at 25% maximum power while on ice. Following sonication, the samples were centrifuged at 12,000× *g* for 15 min at 4 °C. The supernatants were analyzed using the Glucose (GO) Assay Kit (Cat# GAGO20, Sigma-Aldrich). In this procedure, 100 µL of the sample or diluted standards was added to a 1.5-mL EP tube, followed by 200 µL of Assay Reagent. The mixture was incubated at 37 °C for 30 min, and the reaction was stopped by adding 200 µL of 6 M H_2_SO_4_. The OD_540_ was measured using a SpectraMax M5 microplate reader (Molecular Devices) with SoftMax Pro software (v5.4.1.1). The concentration of total protein was quantified using a BCA assay kit (Cat# 20201ES86, Yeasen), and the glucose concentration was normalized to tissue protein (μg glucose/mg protein).

### Statistical analysis

Statistical analyses were performed using Prism 9 (GraphPad Software), except for the survival curves, which were analyzed with SPSS 27.0 (IBM) using the log-rank (Mantel–Cox) test. Each group of data was subjected to the Kolmogorov–Smirnov test, Anderson–Darling test, D’Agostino–Pearson omnibus test, or Shapiro–Wilk test for normal distribution when applicable. An unpaired two-sided Student’s *t*-test was used to determine the significance of differences between two groups of normally distributed data. Welch’s correction was used for groups with unequal variances. An unpaired two-sided Mann–Whitney test was used to determine the significance of differences between data without a normal distribution. For comparisons among multiple groups with one fixed factor, an ordinary one-way ANOVA was used, followed by Tukey’s or Dunnett’s multiple comparisons test as specified in the legends. For comparisons among multiple groups with two fixed factors, an ordinary two-way ANOVA was used, followed by Tukey’s or Sidak’s multiple comparisons test as specified in the legends. The assumption of homogeneity of error variances was tested using an *F*-test (*P* > 0.05). The adjusted means ± SEM were recorded when the analysis met the above standards. Differences were considered significant when *P* < 0.05 or *P* > 0.05 with large differences in observed effects (as suggested in refs. ^145,146^).

## Supplementary information

Supplementary information, Figure S1

Supplementary information, Figure S2

Supplementary information, Figure S3

Supplementary information, Figure S4

Supplementary information, Figure S5

Supplementary information, Figure S6

Supplementary information, Figure S7

Supplementary information, Figure S8

Supplementary information, Figure S9

Supplementary information, Figure S10

Supplementary information, Figure S11

Supplementary information, Figure S12

Supplementary information, Figure S13

Supplementary information, Figure S14

Supplementary information, Figure S15

Supplementary information, Table S1

Supplementary information, Table S2

Supplementary information, Table S3

Full scans

## Source data

Source Data

## Data Availability

The data supporting the findings of this study are available within the paper and its Supplementary information files. The raw RNA sequencing data corresponding to the expression of chemokines in mouse tissues have been deposited in the Genome Sequence Archive^147^ at the National Genomics Data Center, China National Center for Bioinformation/Beijing Institute of Genomics, Chinese Academy of Sciences (GSA: CRA019674 and CRA019771) and are publicly accessible at https://ngdc.cncb.ac.cn/gsa. Materials and reagents are available upon request. Questions regarding the details of the experiments are welcome. Full immunoblots are provided as Supplementary information, full scans. Raw data and the statistical analysis data are provided as a “Source data” file.

## Code availability

The analyses were performed using standard protocols with previously described computational tools. No custom code was used in this study.
