# Supplementary material for: Glucose starvation mimetic aldometanib removes immune barriers permitting mice with hepatocellular carcinoma to live to normal ages
Source: Cell Res. 2025 Nov 25;35(12):934–53. doi: 10.1038/s41422-025-01195-4 (PMC12690099; doi:10.1038/s41422-025-01195-4)
Supplement: Supplementary file 1 — Supplementary information, Figure S1 [file 41422_2025_1195_MOESM1_ESM.pdf]

Supplementary information, Figure S1

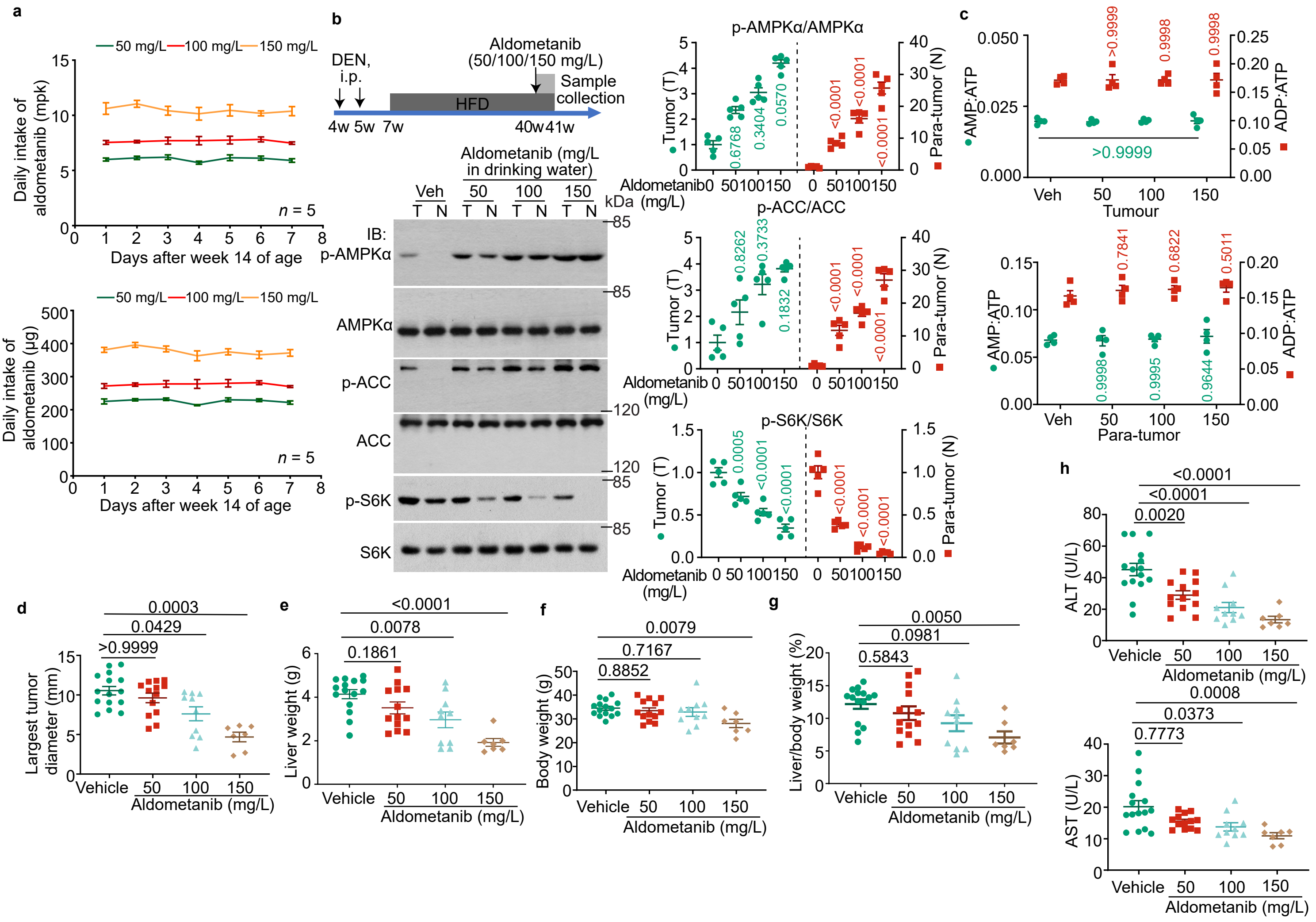

Fig. S1 Aldometanib inhibits hepatocellular carcinomas in a dose-dependent manner.

a Daily intake of aldometanib in DEN-HFD mice. Mice were induced to develop HCC using DEN and HFD, and treated with aldometanib as in Fig. 1b. At 12 weeks of age, the mice absorbed aldometanib dissolved in drinking water at concentrations of 50, 100, or 150 mg/L. After two weeks of treatment, the daily intake of aldometanib for each mouse was recorded over the next week. Data are shown as means ± s.e.m., n = 5 mice.

b, **c** Aldometanib activates AMPK in the liver tissues of DEN-HFD mice. Wildtype C57BL/6 mice (4 weeks old) were intraperitoneally injected with DEN once a week for 2 weeks, followed by feeding with HFD 2 weeks later (depicted in the upper left panel of **b**). At 40 weeks old (after the formation of HCC, validated in Supplementary information, Fig S2a), mice were treated with aldometanib dissolved in drinking water at 50, 100 or 150 mg/L for 7 days. Mice were then euthanized, and the HCC (tumor; T) and para-HCC (para-tumor; N) tissues were freeze-clamped. AMPK activation and mTORC1 inhibition were determined by immunoblotting (**b**, representative blots are shown on the left lower panel; the band intensities of blots from five independent experiments were quantified to calculate the ratios of p-AMPKα/AMPKα, p-ACC/ACC and p-S6K/S6K, and are shown on the right panel (means ± s.e.m., n = 5 mice for each treatment, with P values calculated by two-way analysis of variance (ANOVA), followed by Tukey's test)), and the ratios of AMP:ATP and ADP:ATP were determined by HPLC-MS (**c**, data are means ± s.e.m., n = 4 mice, and P values calculated by two-way ANOVA, followed by Tukey's test).

d-h Mice were induced to develop HCC using DEN and HFD, and treated with aldometanib as in Fig. 1b, followed by collection of tissue (**d**-**g**) and serum (**h**) samples at week 48 of age. The largest tumor diameters (**d**), liver weights (**e**), body weights (**f**), liver:body weight ratios (**g**), and serum ALT (**h**, upper panel) and AST (**h**, lower panel) were then determined. Data are shown as means ± s.e.m., n = 15 (vehicle), 13 (50 mg/L), 10 (100 mg/L), or 7 (150 mg/L) mice, with P values calculated by one-way ANOVA, followed by Dunnett's test (**e**, **f**, **g**, and upper panel of **h**), or by Kruskal-Wallis test, followed by Dunn's test (**d**, and lower panel of **h**).

Experiments in this figure were performed three times.
