# Supplementary material for: Glucose starvation mimetic aldometanib removes immune barriers permitting mice with hepatocellular carcinoma to live to normal ages
Source: Cell Res. 2025 Nov 25;35(12):934–53. doi: 10.1038/s41422-025-01195-4 (PMC12690099; doi:10.1038/s41422-025-01195-4)
Supplement: Supplementary file 2 — Supplementary information, Figure S2 [file 41422_2025_1195_MOESM2_ESM.pdf]

Supplementary information, Figure S2

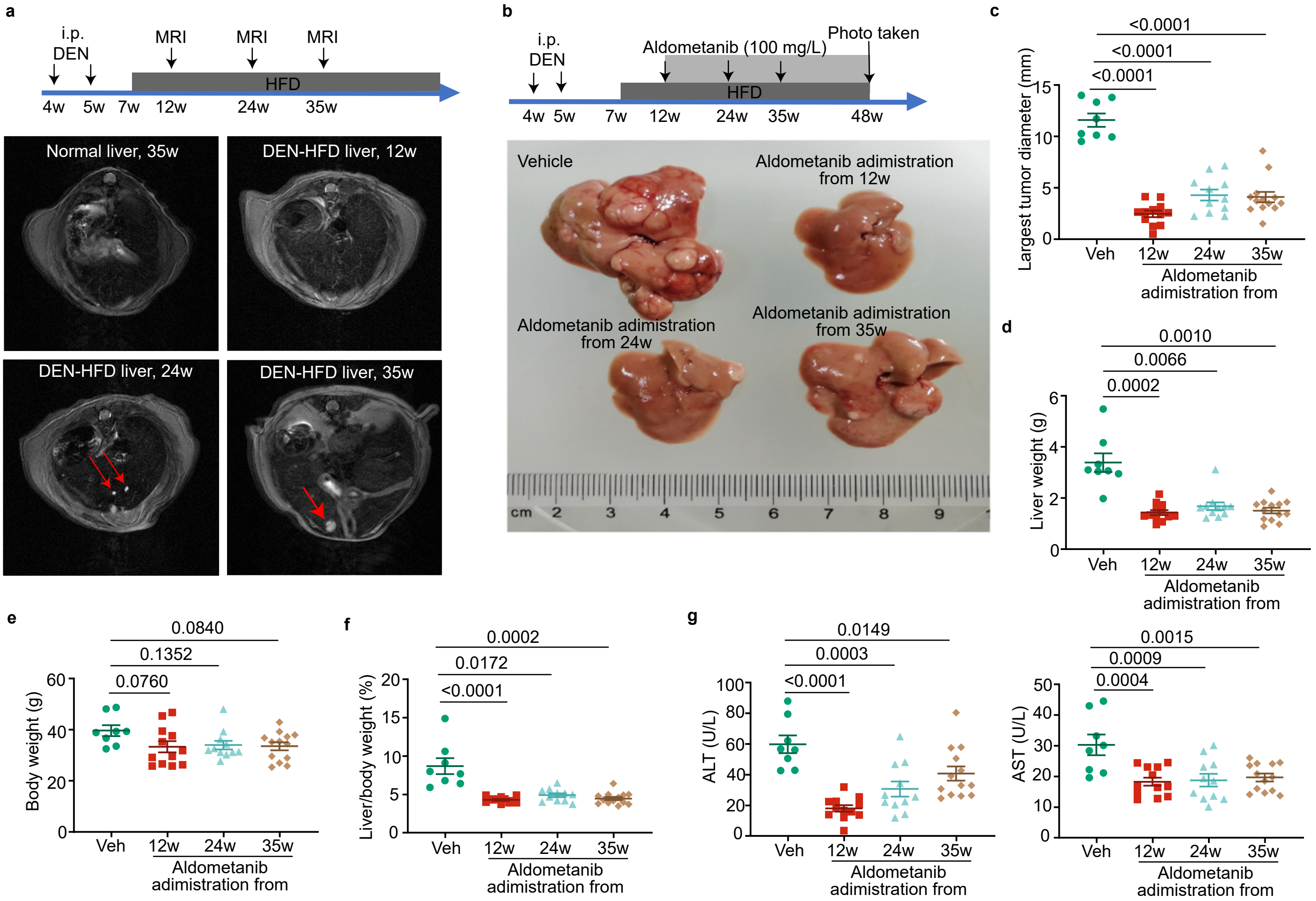

Fig. S2 Aldometanib is effective in inhibiting late-stage HCC.
a Timeline for the development of HCC in the DEN-HFD mice. Mice were induced to develop HCC using DEN and HFD as in Fig. 1a. At 12, 24, and 35 weeks old, the mice were subjected to MRI (depicted in the upper panel, using the DEN- and HFD-untreated normal mice as controls). Representative MRI images are shown in the lower panel, with red arrows pointing to solid HCC tumors.
b-**g** Aldometanib effectively inhibits late-stage HCC. The DEN-HFD mice were treated with aldometanib as in Fig. 1c. The appearance of liver tissues (**b**), largest tumor diameters (**c**), liver weights (**d**), body weights (**e**), liver:body weight ratios (**f**), and serum ALT (**g**, left panel) and AST (**g**, right panel) were determined. Data are shown as means ± s.e.m., $n = 8$ (vehicle), 12 (12 weeks old), 11 (24 weeks old), or 13 (35 weeks old) mice, with P values calculated by one-way ANOVA, followed by Dunnett's test (**c**, **e**, **g**), or by Kruskal-Wallis test, followed by Dunn's test (**d**, **f**).
Experiments in this figure were performed three times.
