# Supplementary material for: Glucose starvation mimetic aldometanib removes immune barriers permitting mice with hepatocellular carcinoma to live to normal ages
Source: Cell Res. 2025 Nov 25;35(12):934–53. doi: 10.1038/s41422-025-01195-4 (PMC12690099; doi:10.1038/s41422-025-01195-4)
Supplement: Supplementary file 3 — Supplementary information, Figure S3 [file 41422_2025_1195_MOESM3_ESM.pdf]

Supplementary information, Figure S3

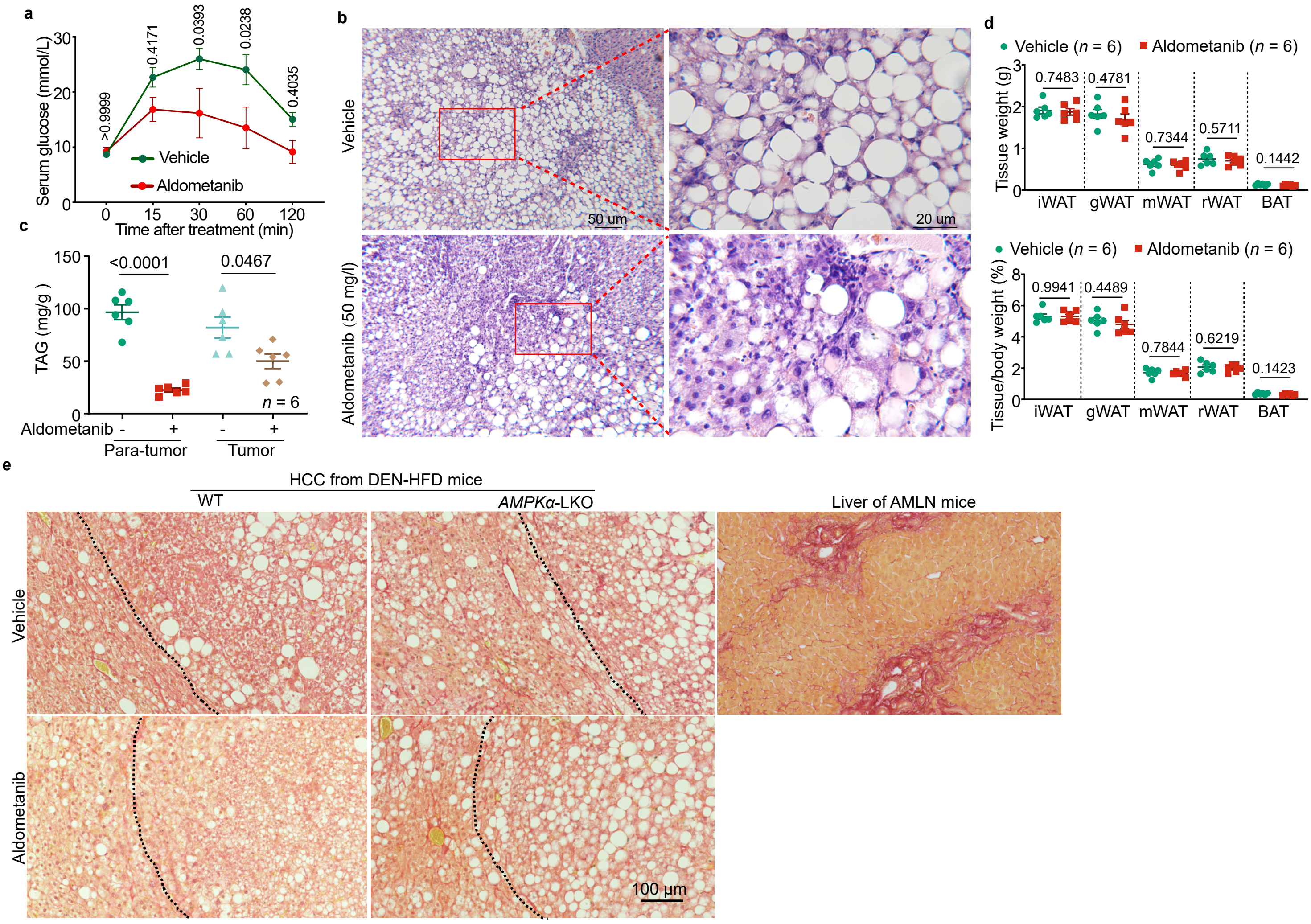

Fig. S3 Aldometanib decreases triglyceride levels in the liver of DEN-HFD mice.

a Aldometanib improves glucose tolerance in DEN-HFD mice. The DEN-HFD mice, induced as described in the upper panel of Fig. 1a, were treated with aldometanib (100 mg/L) starting at 12 weeks of age. At 40 weeks of age, the mice were subjected to an intraperitoneal glucose tolerance test (ipGTT) to assess their glucose tolerance. The data, represented as blood glucose levels, are shown as the mean ± s.e.m.; *n* = 4 mice, and *P* values calculated by two-way ANOVA, followed by Sidak's test.

b, **c** The DEN-HFD mice were treated with aldometanib as in Fig. 1b, followed by the collection of liver tissue samples at week 48 of age. Representative images from H&E staining (**b**) and the triglyceride (TAG) content (**c**; data are shown as means ± s.e.m., *n* = 6 mice, with *P* values calculated by two-way ANOVA, followed by Tukey) of the liver tissues are shown.

d Aldometanib does not change fat composition in DEN-HFD mice. The DEN-HFD mice, induced as described in the upper panel of Fig. 1a, received aldometanib treatment starting at 12 weeks of age. Mice were then given aldometanib at 100 mg/L for 36 weeks, after which the fat composition was analyzed. Data are shown as the mean ± s.e.m.; *n* = 6 mice, and *P* values calculated by two-sided Student's *t*-test. The fat tissues evaluated included inguinal white adipose tissue (iWAT), gonadal white adipose tissue (gWAT), mesenteric white adipose tissue (mWAT), perirenal white adipose tissue (rWAT), and brown adipose tissue (BAT).

e Aldometanib had no effect on fibrosis in both the tumor and para-tumor tissues of DEN-HFD mice. Mice were induced to develop HCC using DEN and HFD as described in the upper panel of Fig. 1a, received 100 mg/L aldometanib starting at 12 weeks of age, and were housed until 48 weeks of age. The extent of fibrosis in tumor and para-tumor tissues were then determined. Representative images are displayed in the left panel, with a positive control from samples of the liver of mice fed with AMLN (Amylin Liver NASH) for 16 weeks shown in the right panel. The scale bars in the images are 100 µm.

Experiments in this figure were performed three times.
