# Supplementary material for: Glucose starvation mimetic aldometanib removes immune barriers permitting mice with hepatocellular carcinoma to live to normal ages
Source: Cell Res. 2025 Nov 25;35(12):934–53. doi: 10.1038/s41422-025-01195-4 (PMC12690099; doi:10.1038/s41422-025-01195-4)
Supplement: Supplementary file 4 — Supplementary information, Figure S4 [file 41422_2025_1195_MOESM4_ESM.pdf]

Supplementary information, Figure S4

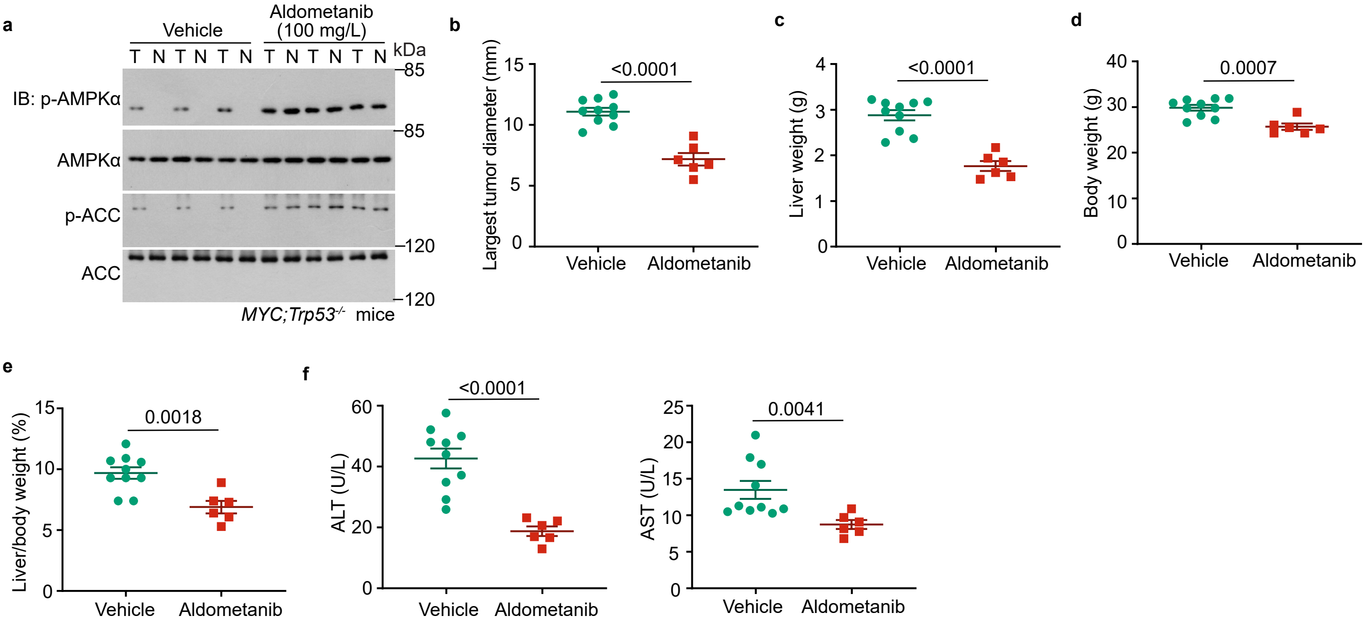

Fig. S4 Aldometanib inhibits HCC in *MYC*;*Trp53*$^{-/-}$ mice.

a-**f** The *MYC*;*Trp53*$^{-/-}$ HCC mice were established and treated with aldometanib as in Fig. 1e. At week 18, mice were euthanized, followed by determination of AMPK activation (**a**), largest tumor diameters (**b**), liver weights (**c**), body weights (**d**), liver:body weight ratios (**e**), and serum ALT (**f**, left panel) and AST (**f**, right panel). Data are shown as means ± s.e.m., n = 10 (vehicle) or 6 mice (aldometanib), with P values calculated by two-sided Student's t-test (**b**-**e**), or by two-sided Student's t-test with Welch's correction (**f**).
Experiments in this figure were performed three times.
