# Supplementary material for: Glucose starvation mimetic aldometanib removes immune barriers permitting mice with hepatocellular carcinoma to live to normal ages
Source: Cell Res. 2025 Nov 25;35(12):934–53. doi: 10.1038/s41422-025-01195-4 (PMC12690099; doi:10.1038/s41422-025-01195-4)
Supplement: Supplementary file 5 — Supplementary information, Figure S5 [file 41422_2025_1195_MOESM5_ESM.pdf]

Supplementary information, Figure S5

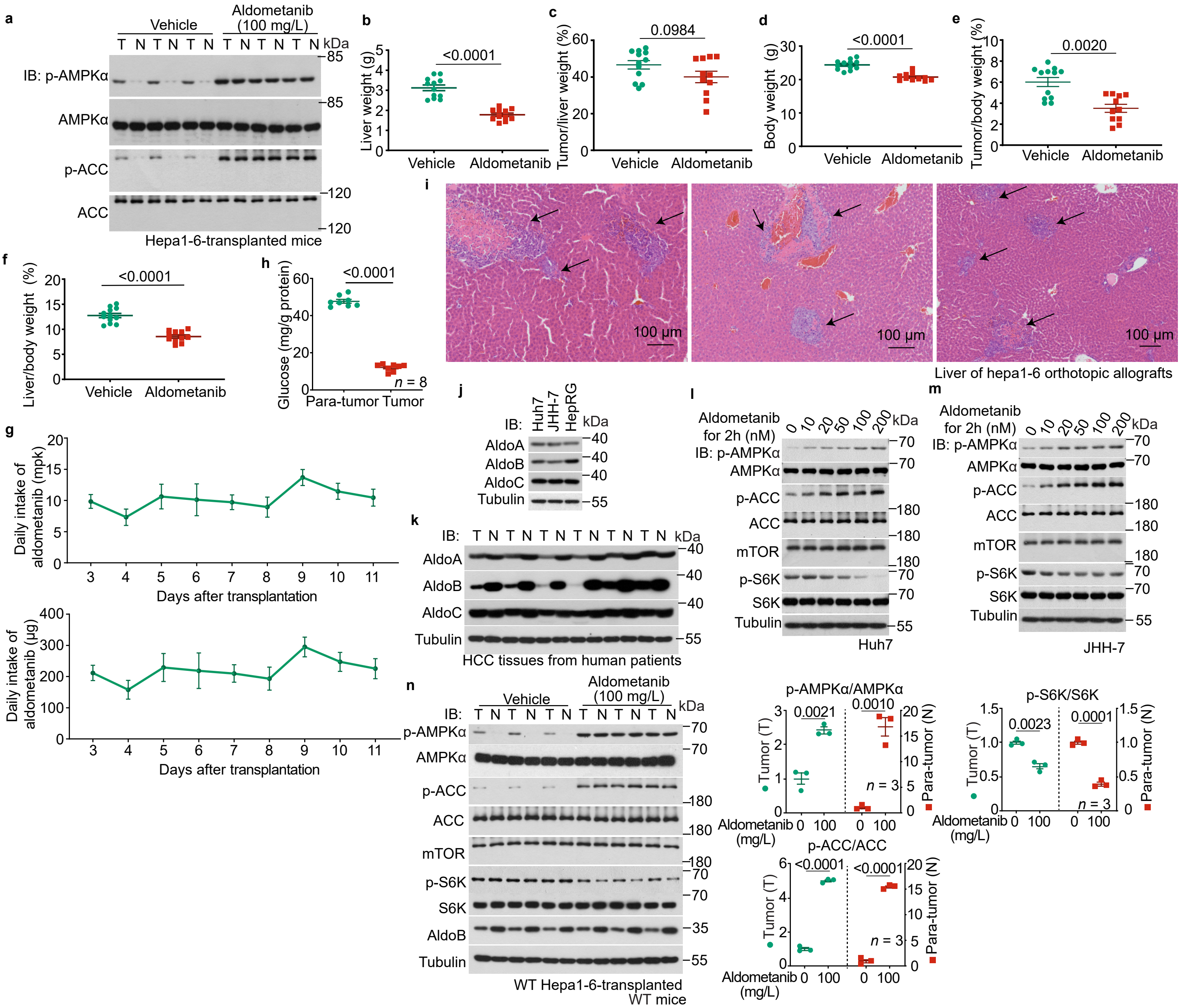

Fig. S5 Aldometanib inhibits HCC orthotopic allografts.

a-f Mice were transplanted with Hepa1-6 cells into the left liver lobes to develop solid tumors, followed by treatment with aldometanib, as in Fig. 1f. At day 17, mice were euthanized, and the HCC (tumor; T) and para-HCC (para-tumor; N) tissues were freeze-clamped, followed by determination of AMPK activation (**a**), liver weights (**b**), tumor:liver weight ratios (**c**), body weights (**d**), tumor:body weight ratios (**e**), and liver:body weight ratios (**f**). Data are shown as means ± s.e.m., *n* = 12 (vehicle) or 11 mice (aldometanib), with *P* values calculated by two-sided Student's *t*-test (**b**, **c**, **d**, **f**), or by two-tailed Mann–Whitney test (**e**).

g Daily intake of aldometanib in Hepa1-6-transplanted mice. Wildtype C57BL/6 mice were transplanted with wildtype Hepa1-6 cells and then treated with 75 or 100 mg/L of aldometanib dissolved in drinking water. The treatment began on day 2 post transplantation, as outlined in Fig. 1f, and the intake of aldometanib was recorded for 9 consecutive days, starting on day 3 post-transplantation. Data are shown as means ± s.e.m., *n* = 4 mice for each condition/group.

h Glucose concentrations are lower in the tumor than in the para-tumor tissues. Wildtype C57BL/6 mice were transplanted with wildtype Hepa1-6 cells and treated with aldometanib starting on day 2 post-transplantation (as outlined in Fig. 1f). The glucose concentrations in both tumor and para-tumor tissues were measured on day 17 after transplantation. *n* = 8 mice for each condition/group, *P* values were calculated by two-sided Student's *t*-test.

i Allografts are already formed in the liver after 2 days of orthotopic transplantation of Hepa1-6 cells. Wildtype C57BL/6 mice were transplanted with wildtype Hepa1-6 cells into the left liver lobes. Liver tissue samples were then collected 48 h post transplantation. The representative images from H&E staining of the liver tissues are shown, with black arrows indicating the HCC tumors. The scale bars in the images represent 100 **μm**.

j-m Aldometanib can activate AMPK and inhibit mTORC1 in human HCC cells that have lower protein levels of ALDOB. The protein levels in human HCC cells (Huh7 and JHH-7; **j**), normal human liver cells (HepRG; **j**), and HCC and para-HCC tissues from human patients (**k**) were assessed. The activity of AMPK and mTORC1 in Huh7 and JHH-7 cells was determined after a 2-h treatment with aldometanib (**l**, **m**).

n Aldometanib activates AMPK in allografts derived from Hepa1-6 cells that have lower protein levels of ALDOB. Wildtype C57BL/6 Mice were transplanted with Hepa1-6 cells into the left liver lobes, followed by treatment with aldometanib (as described in Fig. 1f). At day 17, mice were euthanized, and the HCC (tumor; T) and para-HCC (para-tumor; N) tissues were freeze-clamped, followed by determination of AMPK activation, mTORC1 inhibition, and the protein levels of ALDOB. Blots are shown on the left; the band intensities were quantified to calculate the ratios of p-AMPKα/AMPKα, p-ACC/ACC and p-S6K/S6K, and are shown on the right panel (means ± s.e.m., *n* = 3 mice for each treatment, with *P* values calculated by two-sided Student's *t*-test).

Experiments in this figure were performed three times.
