# Supplementary material for: Glucose starvation mimetic aldometanib removes immune barriers permitting mice with hepatocellular carcinoma to live to normal ages
Source: Cell Res. 2025 Nov 25;35(12):934–53. doi: 10.1038/s41422-025-01195-4 (PMC12690099; doi:10.1038/s41422-025-01195-4)
Supplement: Supplementary file 6 — Supplementary information, Figure S6 [file 41422_2025_1195_MOESM6_ESM.pdf]

Supplementary information, Figure S6

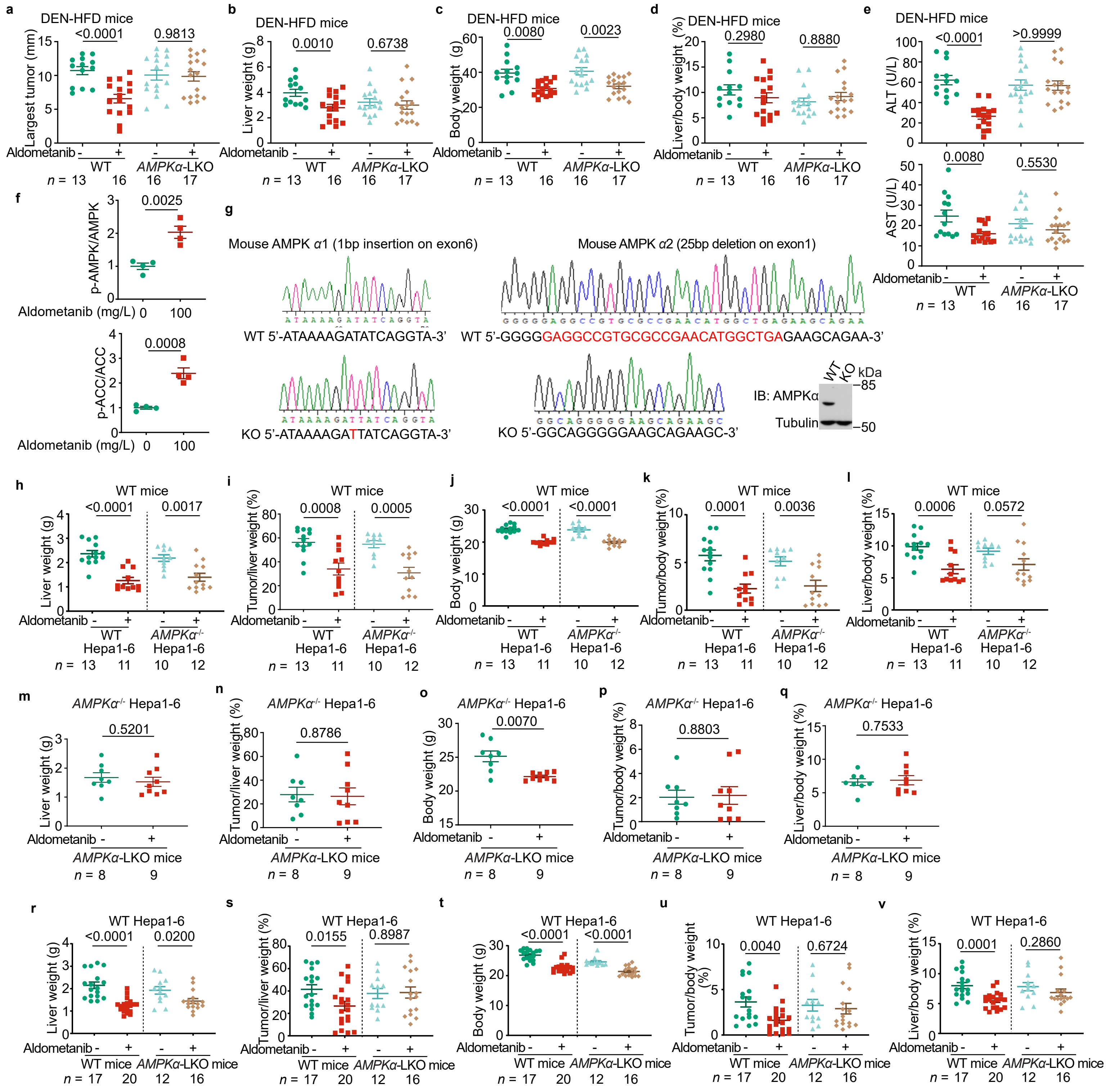


**Fig. S6 AMPK in para-HCC plays a dominant role in aldometanib-induced suppression of HCC.**

**a-e** Liver-specific knockout of AMPKα impairs aldometanib-induced suppression of HCC in DEN-HFD mice. Mice were induced to develop HCC using DEN and HFD and were treated with aldometanib as in Fig. 1a, followed by determination of the largest tumor diameters (**a**), liver weights (**b**), body weights (**c**), liver:body weight ratios (**d**), and serum ALT (**e**, upper panel) and AST (**e**, lower panel). Data are shown as means ± s.e.m., *n* represents the number of mice, and are labelled in each panel, with *P* values calculated by two-way ANOVA, followed by Tukey.

**f** Aldometanib activates AMPK in the tumor of DEN-HFD mice. Mice were induced to develop HCC using DEN and HFD, and were treated with aldometanib as in Fig. 2a, followed by determination of AMPK activation in HCC tissues by immunoblotting. The band intensities of blots from four independent experiments were quantified to calculate the ratios of p-AMPKα/AMPKα, and p-ACC/ACC in the tumor tissues, and data are shown as means ± s.e.m., *n* = 4 mice for each treatment, with *P* values calculated by two-sided Student's *t-test*. See also representative blots, along with the activation of AMPK in non-tumor tissues and in *AMPKα*-KO liver, in Fig. 2a

**g-l** Aldometanib can still inhibit HCC orthotopic allografts of *AMPKα*$^{-/-}$ Hepa1-6 cells. Mice were transplanted with Hepa1-6 cells and treated with aldometanib as in Fig. 2d, followed by determination of liver weights (**h**), tumor:liver weight ratios (**i**), body weights (**j**), tumor:body weight ratios (**k**), and liver:body weight ratios (**l**). Data are shown as means ± s.e.m., *n* represents the number of mice, and are labelled in each panel, with *P* values calculated by two-sided Student's *t*-test (**h**, **i**, **j**, **k** and left panel of **l**), or by two-sided Student's *t*-test with Welch's correction (right panel of **l**). See also the validation data for the *AMPKα*$^{-/-}$ Hepa1-6 cells in **g**, as determined by immunoblotting (right panel) and sequencing (left panel).

**m-q** Aldometanib fails to inhibit HCC orthotopic allografts of *AMPKα*$^{-/-}$ Hepa1-6 cells transplanted into the *AMPKα*-KO liver. Mice were transplanted with Hepa1-6 cells and treated with aldometanib as in Fig. 2f, followed by determination of the liver weights (**m**), tumor: liver weight ratios (**n**), body weights (**o**), tumor:body weight ratios (**p**), and liver:body weight ratios (**q**). Data are shown as means ± s.e.m., *n* = 8 (vehicle) or 9 (aldometanib) mice, with *P* values calculated by two-sided Student's *t*-test (**m**, **n**, **p**, **q**), or by two-sided Student's *t*-test with Welch's correction (**o**).

**r-v** Aldometanib fails to inhibit HCC orthotopic allografts of wildtype Hepa1-6 cells transplanted into the *AMPKα*-KO liver. Mice were transplanted with Hepa1-6 cells and treated with aldometanib as in Fig. 2h, followed by determination of the liver weights (**r**), tumor:liver weight ratios (**s**), body weights (**t**), tumor:body weight ratios (**u**), and liver:body weight ratios (**v**). Data are shown as means ± s.e.m., *n* represents the number of mice, and are labelled in each panel, with *P* values calculated by two-sided Student's *t*-test (right panels of **r** and **u**, and **s**, **t**, **v**), or by two-sided Student's *t*-test with Welch's correction (left panel of **r**, and left panel of **u**).

Experiments in this figure were performed three times.
