# Supplementary material for: Glucose starvation mimetic aldometanib removes immune barriers permitting mice with hepatocellular carcinoma to live to normal ages
Source: Cell Res. 2025 Nov 25;35(12):934–53. doi: 10.1038/s41422-025-01195-4 (PMC12690099; doi:10.1038/s41422-025-01195-4)
Supplement: Supplementary file 7 — Supplementary information, Figure S7 [file 41422_2025_1195_MOESM7_ESM.pdf]

Supplementary information, Figure S7

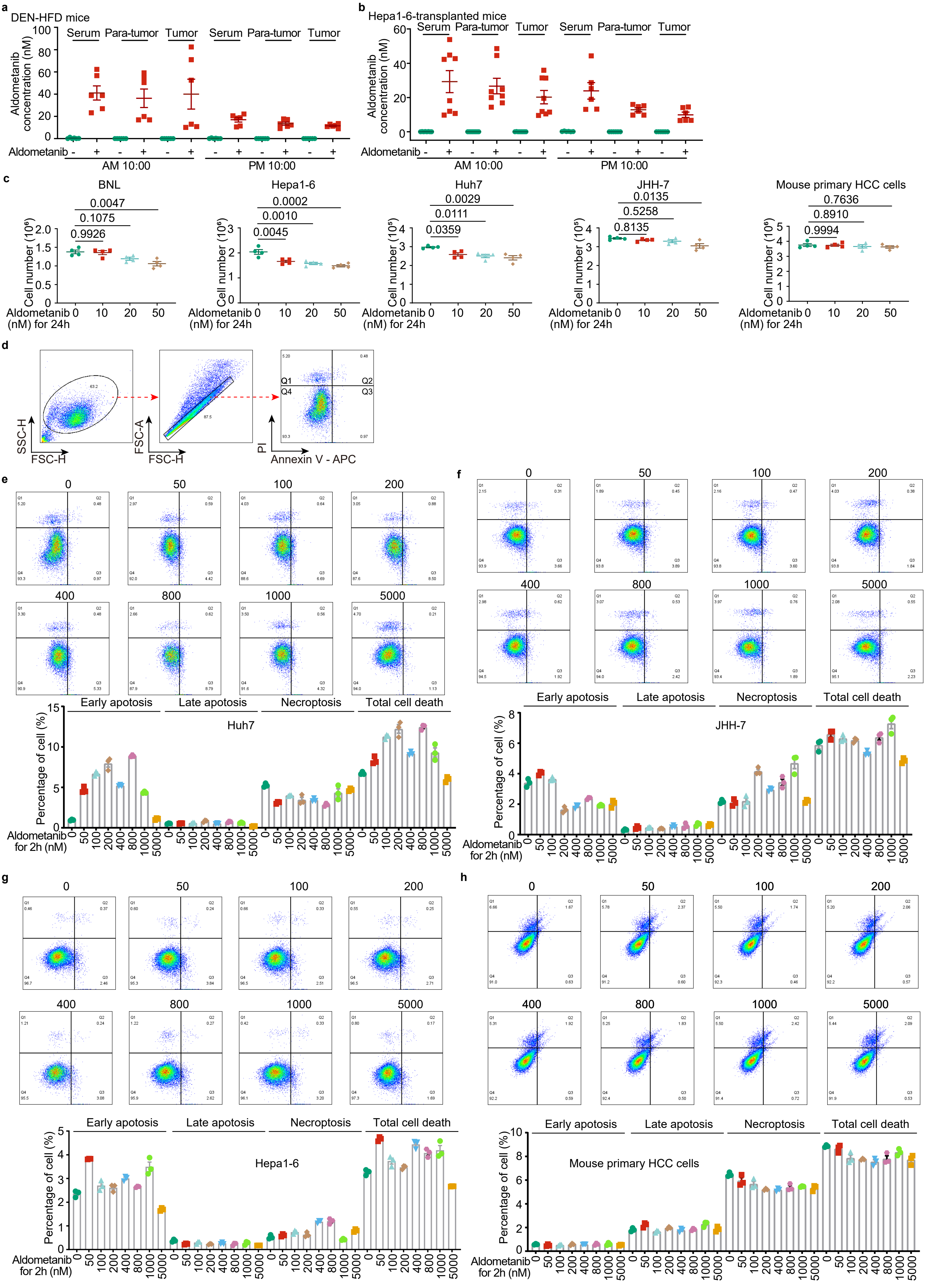

Fig. S7 (cont.)

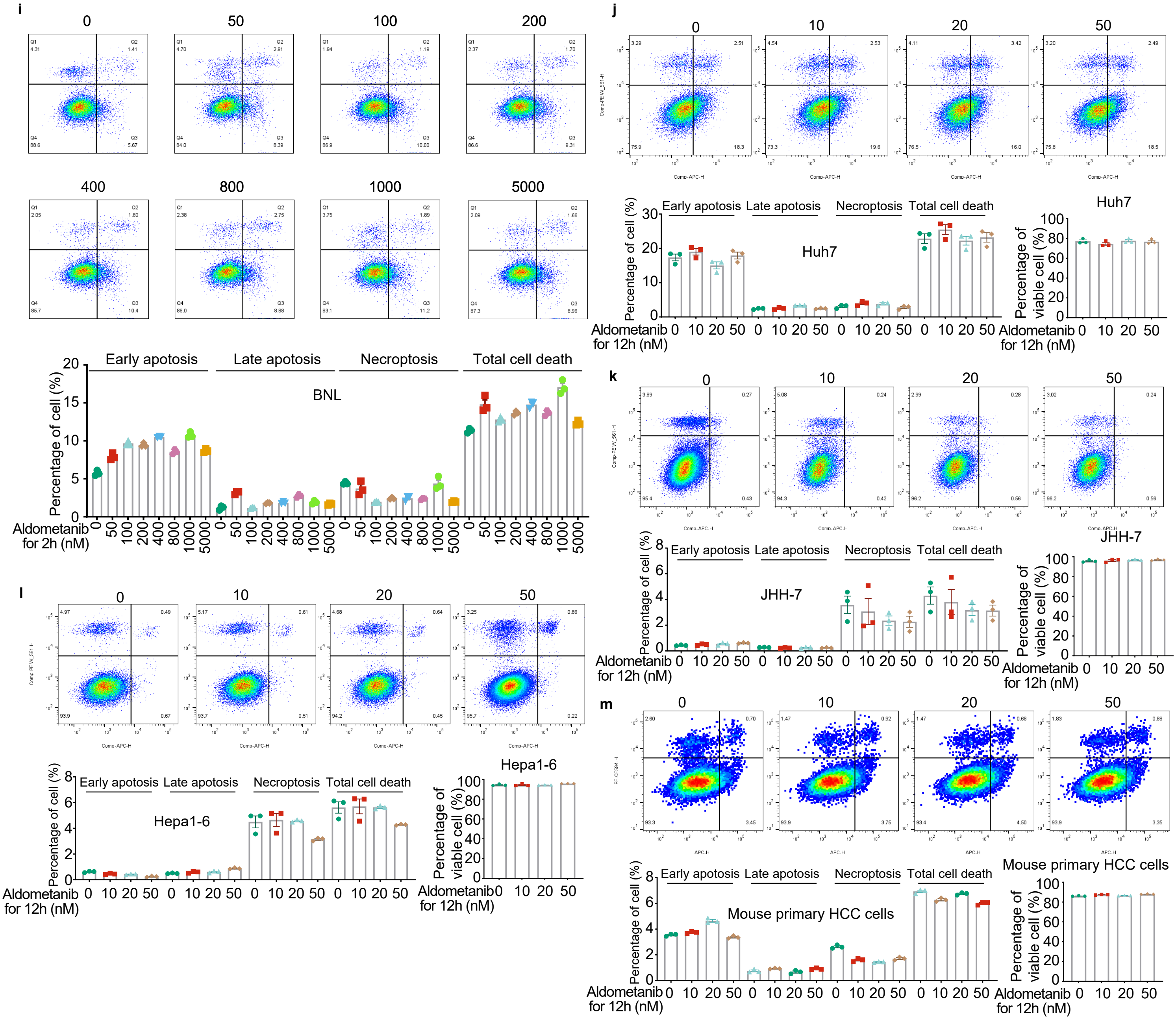

Fig. S7 Aldometanib exhibits little cytotoxicity to cells in culture.

a, **b** Detected concentrations of aldometanib in the serum, liver, and HCC tissues of aldometanib-treated DEN-HFD mice (**a**) and Hepa1-6-transplanted mice (**b**). Mice were induced to develop HCC using DEN and HFD as in Fig. 1a (**a**), or transplanted with Hepa1-6 cells into the left lobe of liver, as in Fig. 1f (**b**). Mice were then treated with 100 mg/L aldometanib at 40 weeks old. At 41 weeks old (**a**), or 1 week after the administration of aldometanib (**b**), mice were euthanized, followed by the determination of aldometanib concentrations in the serum, liver, and HCC tissues (tumor) at the indicated time points. Data are shown as means ± s.e.m., $n = 8$ (10:00 a.m. of **b**) or 6 (others) mice.

c Aldometanib slightly inhibits the growth of HCC cells. The normal mouse liver cell line BNL, HCC cell lines Hepa1-6, Huh7, and JHH-7 cells, as well as the primary HCC cells derived from mouse HCC tissues, were treated with aldometanib at indicated concentrations for 24 h. Cells were then trypsinised, followed by determination of the living cell numbers by Trypan blue staining. Data are shown as means ± s.e.m., $n = 4$ biological replicates, with P values calculated by one-way ANOVA, followed by Tukey.

d Gating strategies used for quantifying the populations of dead cells. During the analysis, intact cells from each sample were selected by FSC-H and SSC-H (left, using a linear scale), followed by FSC-H and FSC-A to exclude doublets (middle, using a linear scale). The fluorescence intensities of propidium iodide (PI) and Annexin V-APC were then determined and presented as density plots (right, using a logarithmic scale). The plot was divided into four quadrants (Q1, Q2, Q3, and Q4), in which Q1 (Annexin V negative and PI positive populations) represents necroptotic cells, Q2 (Annexin V positive and PI positive populations) represents late apoptotic cells, and Q3 (Annexin V positive and PI negative populations) represents early apoptotic cells. The percentage of each type of dead cell was then calculated.

e-i Aldometanib does not trigger apoptosis or necroptosis in cultured HCC cells or normal liver cells after 2 h of treatment. The HCC cell lines, including Huh7 (**e**), JHH-7 (**f**), Hepa1-6 (**g**), the primary HCC cells derived from mouse HCC tissues (**h**), and the normal liver cell line BNL (**i**), were treated with aldometanib at indicated concentrations for 2 h. Cell death was assessed using Annexin V-PI staining followed by flow cytometry. The percentages of early apoptotic, late apoptotic, and necroptotic cells are shown on the bottom of each panel as means ± s.e.m., $n = 3$ biological replicates. See also representative density plots on the top of each panel, and the gating strategy for quantifying each cell population in **d**.

j-m Aldometanib does not trigger apoptosis or necroptosis in cultured HCC cells after 12 h of treatment. Huh7 (**j**), JHH-7 (**k**), Hepa1-6 (**l**) and mouse primary HCC cells (**m**) were treated with aldometanib at indicated concentrations for 12 h. Cell death was assessed as in **e-i** using flow cytometry. Data are shown as means ± s.e.m., $n = 3$ biological replicates. See also representative density plots on the upper of each panel, and the gating strategy for quantifying each cell population in **d**.

Experiments in this figure were performed three times.
