# Supplementary material for: Glucose starvation mimetic aldometanib removes immune barriers permitting mice with hepatocellular carcinoma to live to normal ages
Source: Cell Res. 2025 Nov 25;35(12):934–53. doi: 10.1038/s41422-025-01195-4 (PMC12690099; doi:10.1038/s41422-025-01195-4)
Supplement: Supplementary file 8 — Supplementary information, Figure S8 [file 41422_2025_1195_MOESM8_ESM.pdf]

Supplementary information, Figure S8

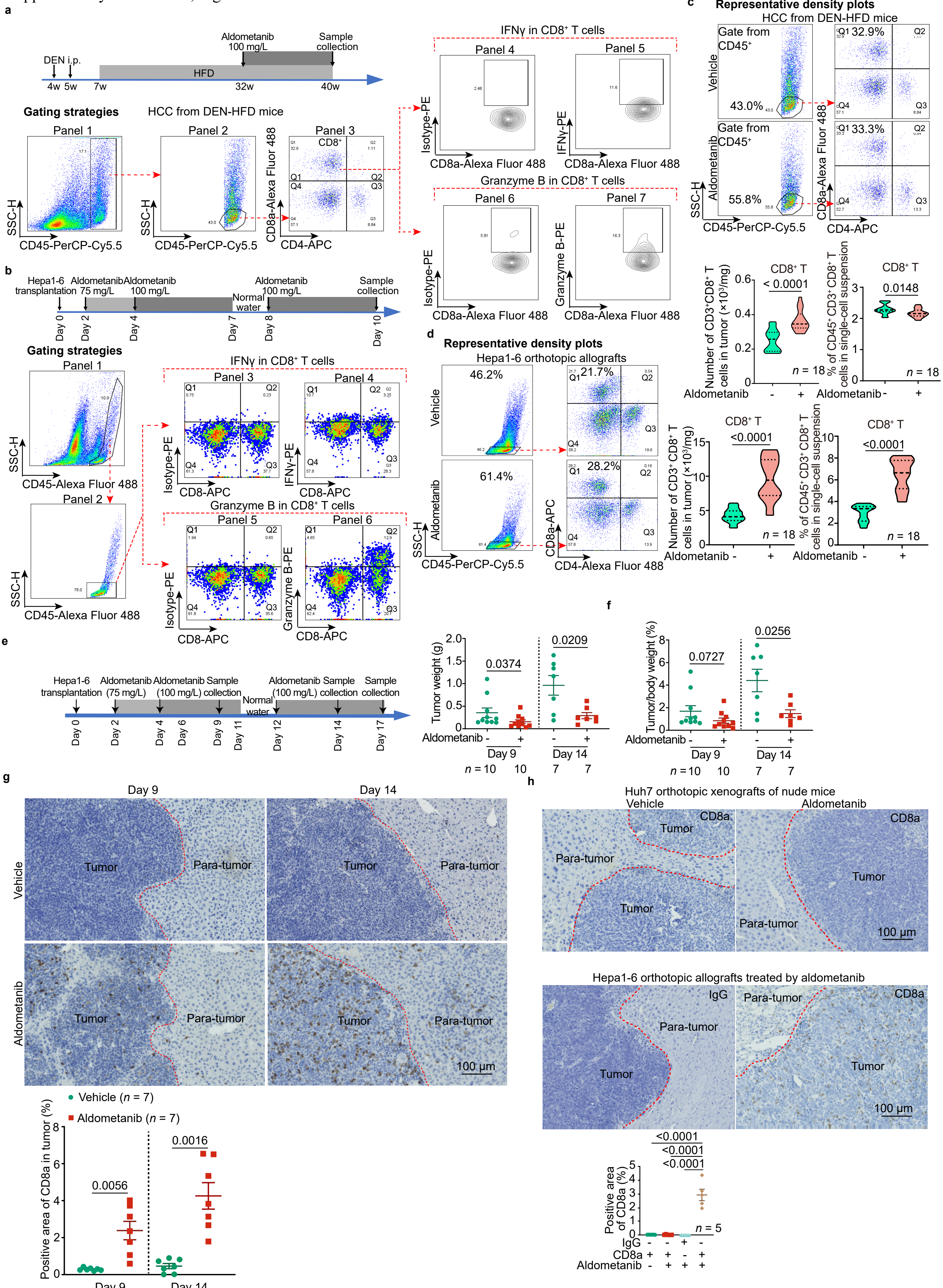

Fig. S8

Supplementary information, Figure S8

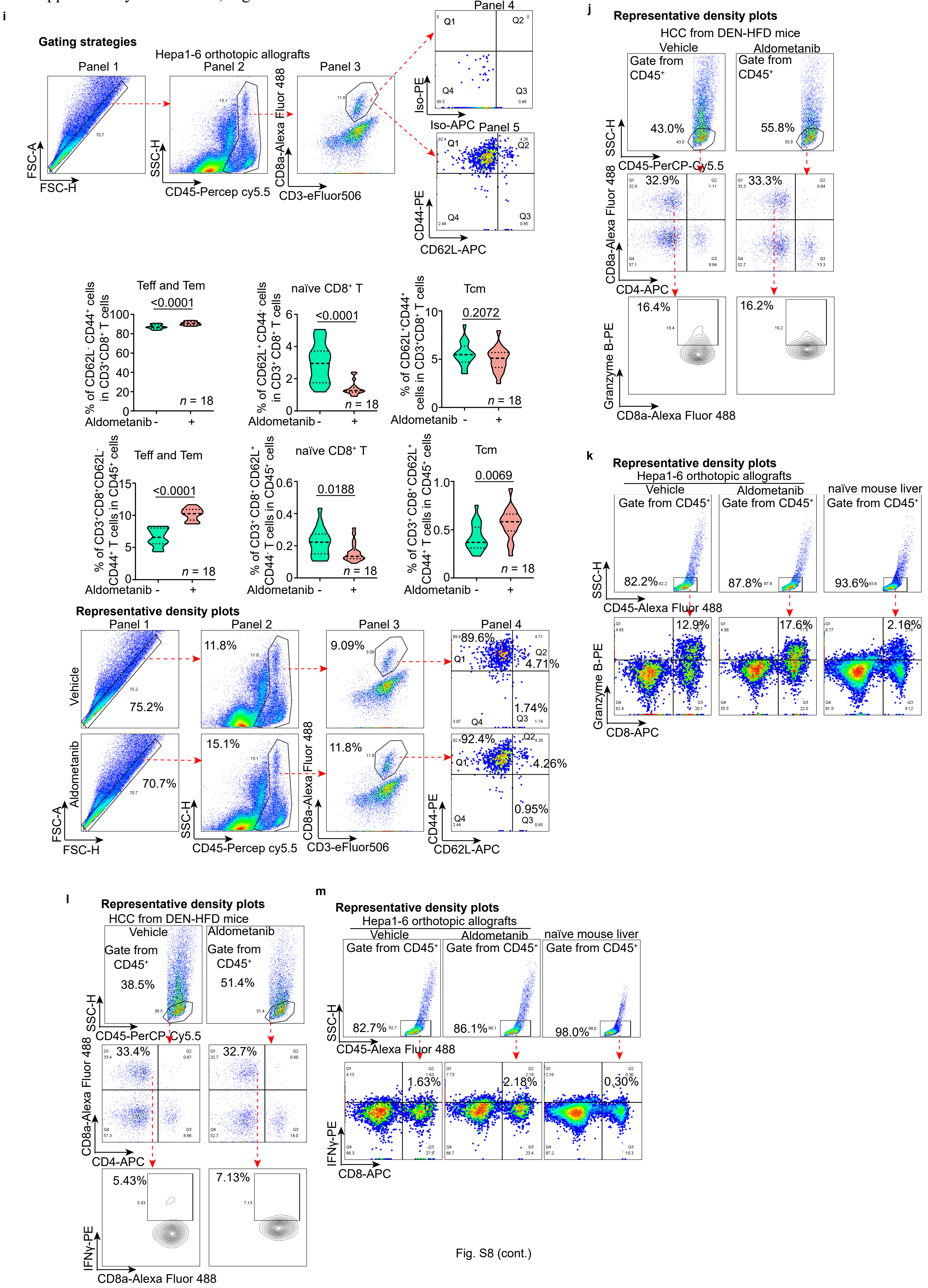

Fig. S8 (cont.)

Supplementary information, Figure S8

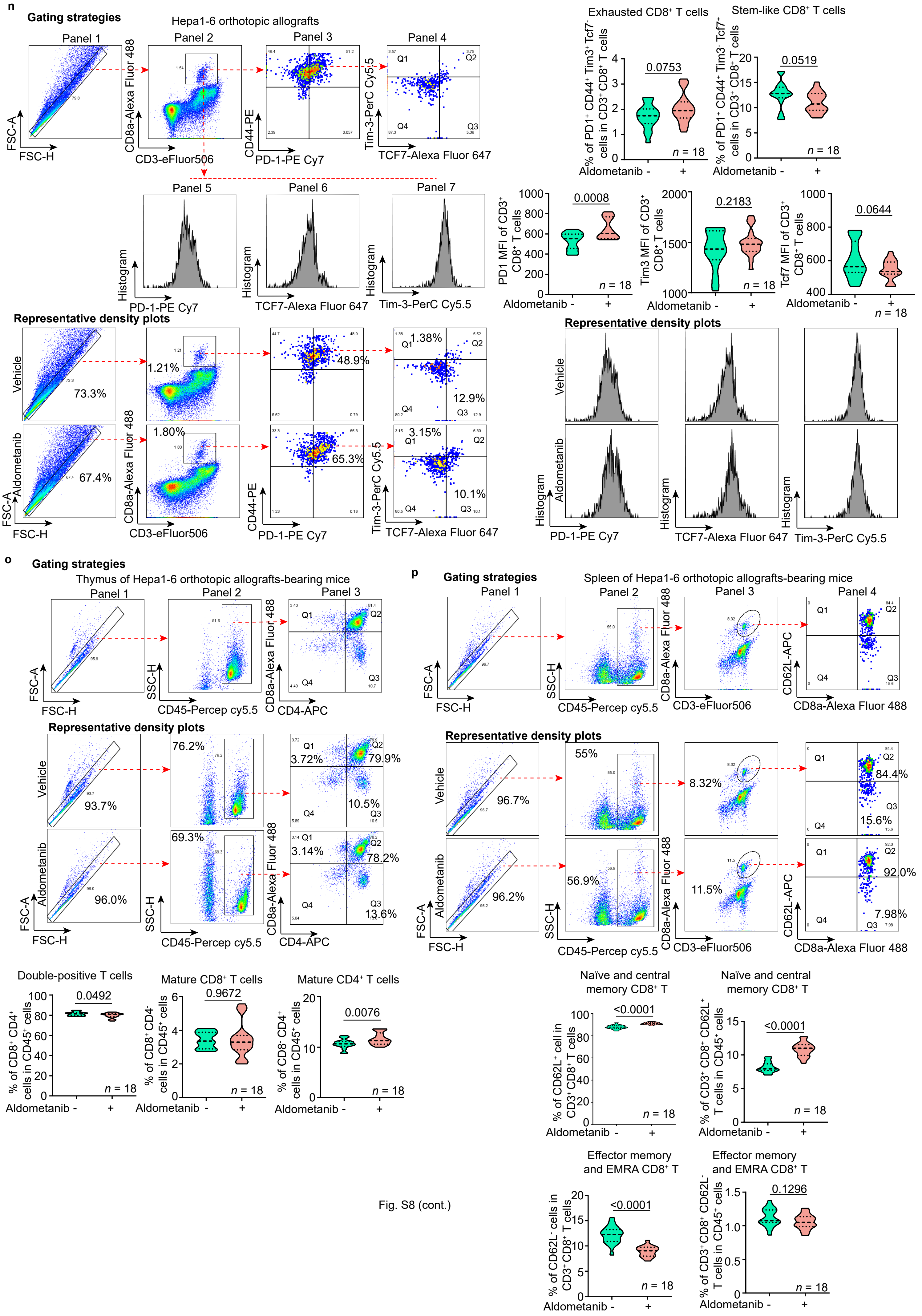

Fig. S8 (cont.)

Fig. S8 Aldometanib induces infiltration of CD8$^+$ T cells into tumors.

a Gating strategies used to quantify CD8$^+$ T cells in DEN-HFD mice. HCC tissues derived from the DEN-HFD mice were digested using type I collagenase to obtain single-cell mixture containing immune cells. The cell mixtures were then incubated with antibodies of CD8a-Alexa Fluor 488, CD4-APC and CD45-PerCP-Cy5.5, along with either granzyme B-PE or IFNγ-PE, or their respective isotype antibody controls that lacked specificity for the target, but matched the class and type of the antibody used to stain the target. To identify CD8$^+$ T cells, the initial gating was performed using a combination of PerCP-Cy5.5 and SSC-H (panel 1), and the PerCP-Cy5.5-positive and SSC-H-low populations were selected (panel 2), which were then plotted with APC against Alexa Fluor 488 (panel 3). The cells identified as positive for Alexa Fluor 488 and negative for APC (Q1 of panel 3) represented the CD8$^+$ T cells, which were then quantified. To determine the expression levels of IFNγ (panels 4 and 5) and granzyme B (panels 6 and 7) in CD8$^+$ T cells, the background fluorescence of PE was first established by using CD8$^+$ T cells stained with isotype control antibody (panels 4 and 6). The CD8$^+$ T cells stained with granzyme B-PE or IFNγ-PE, showing PE intensities above this background (panels 5 and 7), were considered positive for PE and were then quantified. In all cytogram plots, the axes are displayed on a logarithmic scale, except for SSC-H and mean fluorescence intensity (MFI), which are shown on a linear scale; the same applies to all plots below.

b Gating strategies to quantify CD8$^+$ T cells in mice transplanted with Hepa1-6 cells. Experiments were performed as in **a**, but the cell mixture from allografts was not stained with CD4, and the CD45-Alexa Fluor 488 antibody was used to stain CD45. The populations that were positive for Alexa Fluor 488 with low SSC-H (panels 1 and 2) and CD8-APC (panel 3) were identified as CD8$^+$ T cells. To determine the expression levels of IFNγ (panels 3 and 4) and granzyme B (panels 5 and 6) in the CD8$^+$ T cells, those stained with granzyme B-PE or IFNγ-PE and showed PE intensities above the background levels (Q2 of panels 4 and 6, compared to Q2 of panels 3 and 5, which is set by the CD8$^+$ T cells stained with isotype control antibody). The positive cells were then quantified.

c, **d** Aldometanib promotes infiltration of CD8$^+$ T cells into HCC tissues. The HCC tissues from the DEN-HFD mice (**c**) or Hepa1-6-derived orthotopic allografts (**d**) were digested, followed by quantifying CD8$^+$ T cells as in **a** (except that in **d**, the CD45-PerCP Cy5.5, CD8a-APC and CD4-Alexa Fluor 488 antibodies were used to stain CD8$^+$ T cells). Representative density plots are shown the upper of the panel (**c**), or the left of the panel (**d**), with the percentages from gated cell populations labelled on cytograms, and the same hereafter for all cytograms. The percentages of CD8$^+$ T cells in the vehicle group of **c**, for example, is 14.1%, which is calculated by multiplying 43.0% (left panel, representing the CD45-expressing and SSC-H-low populations) by 32.9% (the Q1 of right panel, representing the CD45-expressing and SSC-H-low populations that express CD8a but not CD4). See also statistical analysis data in Fig. 4a (**c**), b (**d**), the lower of **c**, and the right of **d**. Data are shown as means ± s.e.m., n = 18 samples from 6 mice, with P values calculated by two-sided Student's t-test (**c**), or two-sided Student's t-test with Welch' s correction (**d**).

e-g Aldometanib promotes infiltration of CD8$^+$ T cells into HCC tissues after as early as 1 week of treatment. Mice were transplanted with Hepa1-6 cells into the left liver lobes to develop solid tumors, followed by treatment with aldometanib, as in Fig. 1f. At day 9 and day 14, mice were euthanized, followed by determination of tumor weight (**e**) and tumor:body weight ratios (**f**) (shown as means ± s.e.m., n represents the number of mice, and are labelled in each panel, with P values calculated by two-sided Student's t-test with Welch's correction (the right panel of **e**, and **f**), or Mann Whitney test (the left panel of **e**, and **f**)), along with the presence of CD8$^+$ T cells in tumors (**g**; by immunohistochemistry staining; representative images are shown on the upper panels, and the percentages of CD8$^+$ T cells within the tumor were calculated and are shown on the lower panel as means ± s.e.m., n represents the number of mice; P values were calculated by two-sided Student's t-test with Welch's correction). The scale bars are 100 μm.

h Validation for the CD8a antibody for CD8$^+$ T cells through immunohistochemistry staining. The Huh7 orthotopic xenografts collected from CD8$^+$ T-deficient nude mice (established as described in Fig. S11g), along with the Hepa1-6 orthotopic allografts of wildtype C57BL/6J mice, were collected, followed by sectioning and staining using anti-CD8a antibody. Representative images are shown on the upper panels, and the percentages of CD8$^+$ T cells within the tissue were calculated and are shown on the lower panel (means ± s.e.m., n represents the number of mice; P values were calculated by two-way ANOVA, followed by Tukey). The scale bars are 100 μm.

i Aldometanib promotes the infiltration of effector CD8$^+$ T cells and effector memory CD8$^+$ T cells. Hepa1-6-derived orthotopic allografts, collected as in **b**, were digested with type I collagenase, followed by quantification of effector and effector memory CD8$^+$ T cells (T_{eff} and T_{em}; stained with CD45-PerCP-Cy5.5, CD8a-Alexa Fluor 488, CD3-eFluor506, and CD44-PE antibodies), naïve CD8$^+$ T (stained with CD45-PerCP-Cy5.5, CD8a-Alexa Fluor 488, CD3-eFluor506, and CD62L-APC antibodies) and central memory CD8$^+$ T (T_{cm}; stained with CD45-PerCP-Cy5.5, CD8a-Alexa Fluor 488, CD3-eFluor506, CD44-PE, and CD62L-APC antibodies) by flow cytometry. Gating strategies are shown on the upper panel, where the FSC-A, FSC-H (panel 1), CD45-PerCP-Cy5.5-positive, SSC-H (panel 2), CD8a-Alexa Fluor 488-positive, CD3-eFluor506-positive (panel 3), CD44-PE-positive, and CD62L-APC-negative (Q1 of panel 5) populations were identified as CD8$^+$ T_{eff} and CD8$^+$ T_{em}. The FSC-A, FSC-H (panel 1), CD45-PerCP-Cy5.5-positive, SSC-H (panel 2), CD8a-Alexa Fluor 488-positive, CD3-eFluor506-positive (panel 3), CD44-PE-negative, and CD62L-APC-positive (Q3 of panel 5) populations were identified as naïve CD8$^+$ T. The FSC-A, FSC-H (panel 1), CD45-PerCP-Cy5.5-positive, SSC-H (panel 2), CD8a-Alexa Fluor 488-positive, CD3-eFluor506-positive (panel 3), CD44-PE-positive, and CD62L-APC-positive (Q2 of panel 5) populations were identified as CD8$^+$ T_{cm}. The iso-PE and iso-APC were used as negative control. The representative density plots are shown on the lower panel, and the statistical analysis data on the middle panel (means ± s.e.m., n = 18 samples from 6 mice, with P values calculated by two-sided Student's t-test with Welch's correction (percentage of CD62L$^+$CD44$^-$ cells in CD3$^+$CD8$^+$ T), and the others by two-sided Student's t-test).

j-m Tumoricidal activity can be detected in the CD8$^+$ T cells found in HCC. Experiments were performed as in Fig. 4e-h, and the representative density plots for the populations of CD8$^+$ T cells with expression of granzyme B (**j**, **k**) and IFNγ (**l**, **m**) are shown. See also the gating strategies in **a** (**j**, **l**) and **b** (**k**, **m**).

n Aldometanib does not promote the infiltration of exhausted CD8$^+$ T cells or stem-like CD8$^+$ T cells. The Hepa1-6-derived orthotopic allografts, collected as in **b**, were digested with type I collagenase, followed by quantification of exhausted CD8$^+$ T cells (stained with CD3-eFluor506, CD8a-Alexa Fluor 488, CD44-PE, PD-1-PE Cy7, and Tim-3-PerC Cy5.5 antibodies) and stem-like CD8$^+$ T cells (stained with CD3-eFluor506, CD8a-Alexa Fluor 488, CD44-PE, PD-1-PE Cy7, and TCF7-Alexa Fluor 647 antibodies), by flow cytometry. The gating strategies are shown on the left upper panel, where the FSC-A, FSC-H (panel 1), CD8a-Alexa Fluor 488-positive, CD3-eFluor506-positive (panel 2), CD44-PE-positive, PD-1-PE-cy7-positive (panel 3), Tim-3-PerC Cy5.5-positive, and TCF7-Alexa Fluor 647-negative (Q1 of panel 4) populations were identified as exhausted CD8$^+$ T cells. The FSC-A, FSC-H (panel 1), CD8a-Alexa Fluor 488-positive, CD3-eFluor506-positive (panel 2), CD44-PE-positive, PD-1-PE Cy7-positive (panel 3), Tim-3-PerC Cy5.5-negative, and TCF7-Alexa Fluor 647-positive (Q3 of panel 4) populations were identified as stem-like CD8$^+$ T cells. To determine the expression of exhausted CD8$^+$ T cells and stem-like CD8$^+$ T cells, the MFI (geometric mean value) of PD-1, Tim3, and Tcf in the CD8a-Alexa Fluor 488, and CD3-eFluor506 positive populations, as depicted in the left panel (panel 5, panel 6, and panel 7), was further analyzed. The representative density plots are shown on the left lower panel, and the statistical analysis data on the right upper panel (means ± s.e.m., n = 18 samples from 6 mice, with P values calculated by two-sided Student's t-test with Welch's correction (Tcf7 MFI of CD3$^+$CD8$^+$ T cells), and the others by two-sided Student's t-test).

o Aldometanib does not affect the maturation of CD8$^+$ T cells in the thymus. The thymus of Hepa1-6-derived orthotopic allografts-bearing mice, which is collected as in **b**, was dispersed in PBS, followed by quantification of mature CD8$^+$ T cells (stained with CD45-PerCP-Cy5.5, and CD8a-Alexa Fluor 488 antibodies), mature CD4$^+$ T cells (stained with CD45-PerCP-Cy5.5, and CD4-APC antibodies), and double-positive T cells (stained with CD45-PerCP-Cy5.5, CD8a-Alexa Fluor 488, and CD4-APC antibodies) by flow cytometry. The gating strategies are shown on the upper panel, where the FSC-A, FSC-H (panel 1), CD45-PerCP-Cy5.5-positive, SSC-H (panel 2), CD4-APC-negative, CD8a-Alexa Fluor 488-positive (Q1 of panel 3) populations were identified as mature CD8$^+$ T cells. The FSC-A, FSC-H (panel 1), CD45-PerCP-Cy5.5-positive, SSC-H (panel 2), CD4-APC-positive, CD8a-Alexa Fluor 488-negative (Q3 of panel 3) populations were identified as mature CD4$^+$ T cells. The FSC-A, FSC-H (panel 1), CD45-PerCP-Cy5.5-positive, SSC-H (panel 2), CD4-APC-positive, CD8a-Alexa Fluor 488-positive (Q2 of panel 3) populations were identified as double-positive T cells. The representative density plots are shown on the middle panel, and the statistical analysis data on the lower panel (means ± s.e.m., n = 18 samples from 6 mice, with P values calculated by two-sided Student's t-test with Welch's correction (mature CD8$^+$ T cells), and the others by two-sided Student's t-test).

p Aldometanib increases the populations of naïve and central memory CD8$^+$ T cells in the spleen. The spleen of Hepa1-6-derived orthotopic allografts-bearing mice, collected as in **b**, was dispersed in PBS, followed by quantification of naïve and central memory CD8$^+$ T cells (stained with CD45-PerCP-Cy5.5, CD62L-APC, CD3-eFluor506 and CD8a-Alexa Fluor 488 antibodies), and CD8$^+$ T_{EMRA} cells (stained with CD45-PerCP-Cy5.5, CD3-eFluor506 and CD8a-Alexa Fluor 488 antibodies) by flow cytometry. The gating strategies are shown on the upper panel, where the FSC-A, FSC-H (panel 1), CD45-PerCP-Cy5.5-positive, SSC-H (panel 2), CD8a-Alexa Fluor 488-positive, CD3-eFluor506-positive (panel 3), CD62L-APC-positive, CD8a-Alexa Fluor 488-positive (Q2 of panel 4) populations were identified as naïve and central memory CD8$^+$ T cells. The FSC-A, FSC-H (panel 1), CD45-PerCP-Cy5.5-positive, SSC-H (panel 2), CD8a-Alexa Fluor 488-positive, CD3-eFluor506-positive (panel 3), CD62L-APC-negative, CD8a-Alexa Fluor 488-positive (Q3 of panel 4) populations were identified as effector memory and EMRA CD8$^+$ T cells. The representative density plots are shown on the middle panel, and the statistical analysis data on the lower panel (means ± s.e.m., n = 18 samples from 6 mice, with P values calculated by two-sided Student's t-test with Welch's correction (percentage of CD62L$^+$ cell in CD3$^+$CD8$^+$ T cells, percentage of CD62L$^-$ cells in CD3$^+$CD8$^+$ T cells), and the others by two-sided Student's t-test).
