# Supplementary material for: Glucose starvation mimetic aldometanib removes immune barriers permitting mice with hepatocellular carcinoma to live to normal ages
Source: Cell Res. 2025 Nov 25;35(12):934–53. doi: 10.1038/s41422-025-01195-4 (PMC12690099; doi:10.1038/s41422-025-01195-4)
Supplement: Supplementary file 9 — Supplementary information, Figure S9 [file 41422_2025_1195_MOESM9_ESM.pdf]

Supplementary information, Figure S9

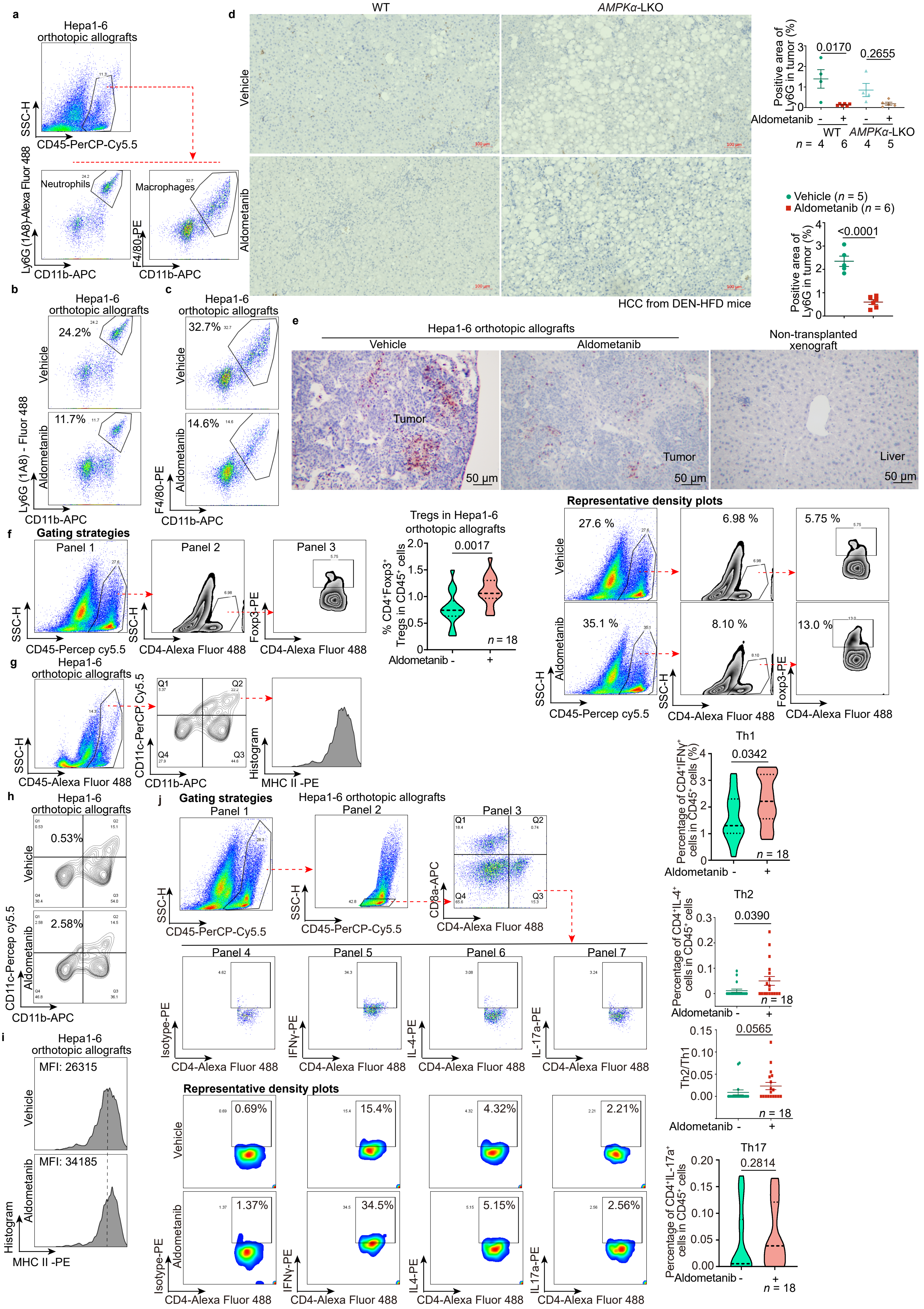

Fig. S9

Supplementary information, Figure S9

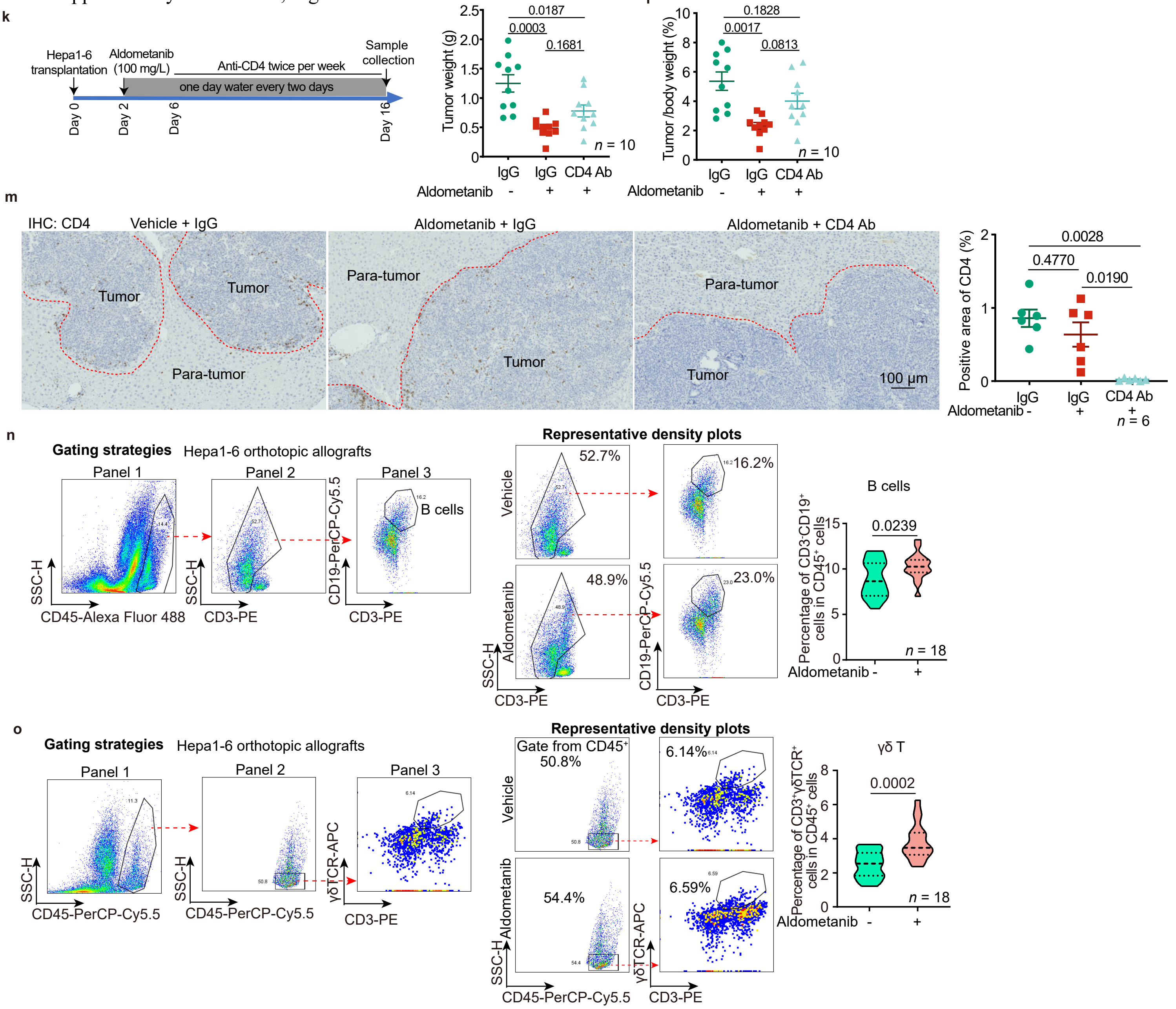

Fig. S9 Aldometanib enables infiltration of CD8$^+$ T cells into tumors.

a-e Aldometanib eases the immune barrier in HCC. The Hepa1-6-derived allografts, collected as in Fig. S8b, were digested with type I collagenase. The quantities of neutrophils (**b**), and macrophages (**c**) were quantified by flow cytometry as described in Fig. 4i (**b**), and j (**c**). The representative density plots are shown in **b**, and **c**. The gating strategies used to quantify the neutrophils and macrophages are shown in **a**. The population that is positive for CD11b-APC and Ly6G (1A8)-Alexa Fluor 488 (**a**) represents neutrophils, and the population that is positive for CD11b-APC and F4/80-PE (**a**) represents macrophages. In addition, the presence of neutrophils in both DEN-HFD-induced HCC (**d**) and Hepa1-6 orthotopic allografts (**e**) was determined by immunohistochemistry staining. Representative images are shown on the left panels (**d**), or lower panel (**e**), and the percentages of Ly6G-positive cells within the tumor area were calculated and are shown on the right panel (**d**), or the upper panel (**e**) (means ± s.e.m., *n* represents the number of mice, and are labelled in each panel; *P* values were calculated by two-sided Student's *t*-test (**e**), or two-way ANOVA, followed by Tukey (**d**)). The scale bars are 50 μm (**e**) or 100 μm (**d**).

f Aldometanib promotes the infiltration of T_{reg} cells. The Hepa1-6-derived allografts, collected as in Fig. S8b, were digested with type I collagenase, followed by quantification of T_{reg} cells (stained with CD45-Percep cy5.5, CD4-Alexa Fluor 488, and Foxp3-PE antibodies) by flow cytometry. See also the gating strategies on the left panel, where the SSC-H, CD45-Percep cy5.5-positive (panel 1), CD4-Alexa Fluor 488-positive (panel 2), CD4-Alexa Fluor 488-positive, and Foxp3-PE-positive (panel 3) populations were identified as T_{reg} cells. Representative density plots are shown on the left panel, and the statistical analysis data on the right panel (means ± s.e.m., *n* = 18 samples from 6 mice, with *P* values calculated by two-sided Student's *t*-test).

g-i Aldometanib promotes infiltration of DCs cells. The quantities of DCs (**h**), and the levels of MHC II expression in the myeloid DCs (**i**) were quantified by flow cytometry as described in Fig. 4k (**h**) and l (**i**). The representative density plots are shown in **h**, and **i**. The gating strategies used to quantify the DCs are shown in **g**. The CD11c-PerCP-Cy5.5-positive and CD11b-negative population represents lymphoid DCs (Q1 of **g**) and the CD11c-PerCP-Cy5.5-positive and CD11b-positive population represents myeloid DCs (Q2 of **g**). To determine the expression of MHC II in myeloid DCs, the MFI (geometric mean value) of PE in the CD11b-APC-positive and CD11c-PerCP-Cy5.5-positive populations, as depicted in **i**, was further analyzed.

j Aldometanib promotes infiltration of Th1 cells. The Hepa1-6-derived orthotopic allografts, collected as in Fig. S8b, were digested with type I collagenase, followed by quantification of CD4$^+$ T cells, including the Th1 subset (stained with CD45-PerCP-Cy5.5, CD4-Alexa Fluor 488, and IFNγ-PE antibodies), the Th2 subset (stained with CD45-PerCP-Cy5.5, CD4-Alexa Fluor 488, and IL-4-PE antibodies), and the Th17 subset (stained with CD45-PerCP-Cy5.5, CD4-Alexa Fluor 488, and IL-17a-PE antibodies), by flow cytometry. Representative density plots are shown on the left lower panel, and the statistical analysis data on the right panel (means ± s.e.m., *n* = 18 samples from 6 mice, with *P* values calculated by two-sided Student's *t*-test (upper panel), or by Mann Whitney test (others)). See also the gating strategies for each subset of CD4$^+$ T cells on the left upper panel, where the CD45-PerCP-Cy5.5-positive, SSC-H-low (panels 1 and 2), and CD4-Alexa Fluor 488-positive, CD8a-APC-negative (Q3 of panel 3) populations were identified as CD4$^+$ T cells. The CD4$^+$ T cells that are positive for IFNγ-PE represent the Th1 subset (panel 5), while those positive for IL-4-PE represent the Th2 subset (panel 6), and the cells positive for IL-17a-PE indicate the Th17 subset (panel 7). All these subsets exhibited higher PE intensities compared to the background PE intensities set by CD4$^+$ T cells stained with isotype controls (panel 4).

k-m CD4$^+$ T cells do not play a major role in the aldometanib-induced suppression of HCC. Mice were transplanted into the left lobe with Hepa1-6 cells, treated with aldometanib, and depleted of CD4$^+$ T cells by intraperitoneally injection of anti-CD4 neutralizing antibody (depicted in the left panel of **k**). HCC tissue samples were collected, followed by determination of the tumor weights (**k**), and tumor:body weight ratios (**l**). The efficacy of CD4$^+$ T cell clearance in the Hepa1-6 allografts was also determined by immunohistochemistry staining (**m**; representative images are shown on the left panels, and the percentages of CD4$^+$ T cells within the tumor area were calculated and are shown on the right panel). Statistical analysis data in this panel are means ± s.e.m., *n* represents the number of mice, and are labelled in each panel, with *P* values calculated by two-way ANOVA, followed by Tukey (**k**, **l**, and **m**). The scale bars are 100 μm.

n Aldometanib promotes the infiltration of B cells. Experiments were performed as in Fig. S8b, except that B cells (stained with CD3-PE, CD19-PerCP-Cy5.5, and CD45-Alexa Fluor 488 antibodies) were determined. Representative density plots are shown on the middle panel, and the statistical analysis data on the right panel (means ± s.e.m., *n* = 18 samples from 6 mice, with *P* values calculated by two-sided Student's *t*-test). See also the gating strategies on the left panel, where the Alexa Fluor 488-positive, PE-negative (panels 1 and 2), and PerCP-Cy5.5-positive (panel 3) populations were identified as B cells.

o Aldometanib promotes infiltration of γδT cells. Experiments were performed as in Fig. S8b, except that γδT cells (labelled with CD3-PE, γδTCR-APC, and CD45-PerCP-Cy5.5 antibodies) were determined. Representative density plots are shown on the middle panel, and the statistical analysis data on the right panel (means ± s.e.m., *n* = 18 samples from 6 mice, with *P* values calculated by two-sided Student's *t*-test). See also the gating strategies on the left panel, where the PerCP-Cy5.5-positive (panel 1), and the PerCP-Cy5.5-positive and SSC-H-low populations were selected (panel 2), which were then plotted with PE-positive, and APC-positive (panel 3) populations were identified as γδT cells.

Experiments in this figure were performed three times.
