# Supplementary material for: Glucose starvation mimetic aldometanib removes immune barriers permitting mice with hepatocellular carcinoma to live to normal ages
Source: Cell Res. 2025 Nov 25;35(12):934–53. doi: 10.1038/s41422-025-01195-4 (PMC12690099; doi:10.1038/s41422-025-01195-4)
Supplement: Supplementary file 10 — Supplementary information, Figure S10 [file 41422_2025_1195_MOESM10_ESM.pdf]

Supplementary information, Figure S10

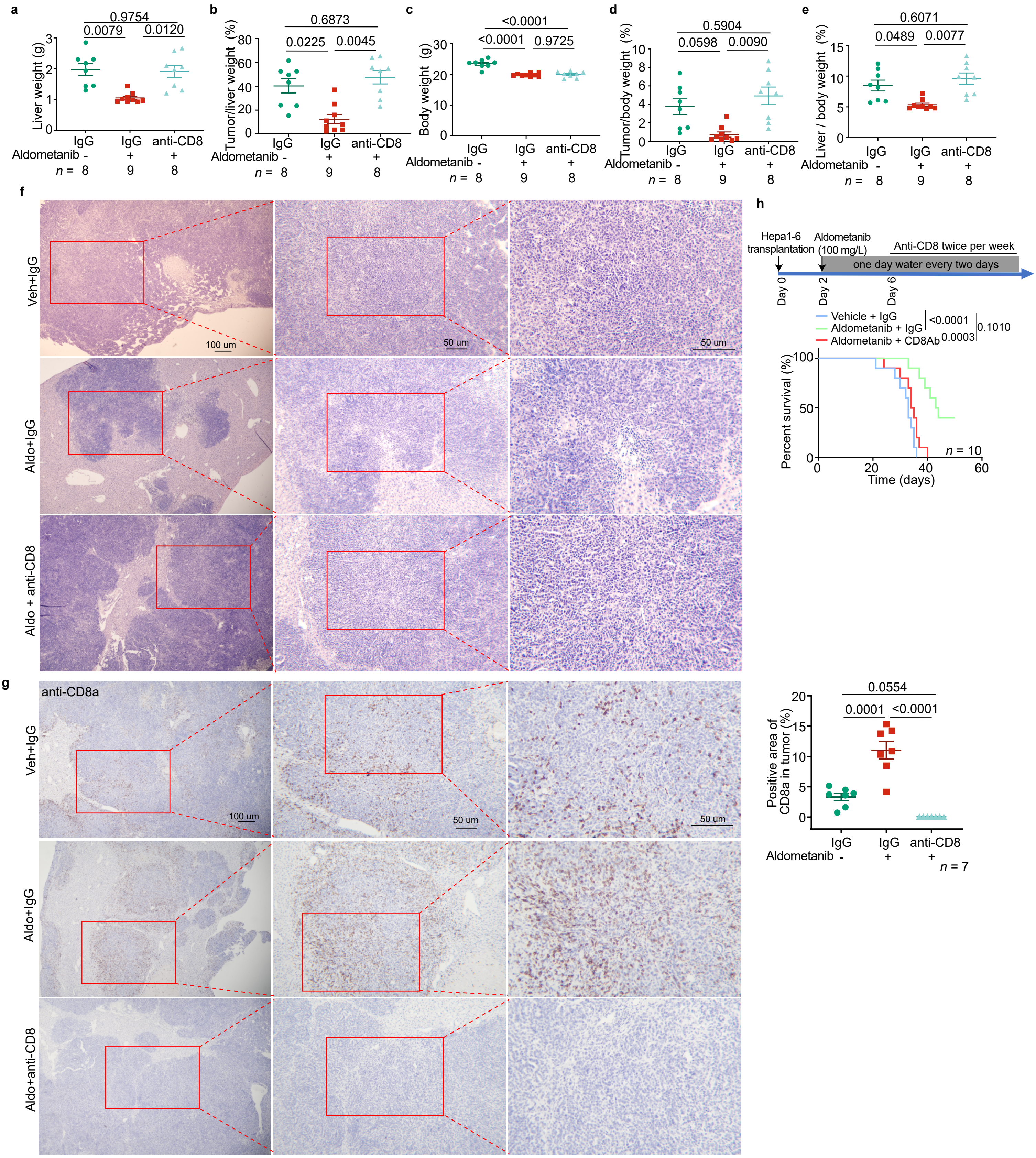

Fig. S10 CD8⁺ T cells are required for aldometanib-induced suppression of HCC.

a-g Depletion of CD8⁺ T cells diminishes the aldometanib-mediated suppression of HCC. Mice were transplanted into the left lobe with Hepa1-6 cells, treated with aldometanib, and depleted of CD8⁺ T cells, as in Fig. 4m. HCC tissue samples were collected, followed by determination of the liver weights (**a**), tumor:liver weight ratios (**b**), body weights (**c**), tumor:body weight ratios (**d**), and liver:body weight ratios (**e**). Data are shown as means ± s.e.m., *n* represents the number of mice, and are labelled in each panel, with *P* values calculated by two-way ANOVA, followed by Tukey), and morphology (**f**; by H&E staining, and **g**; by immunohistochemistry staining of CD8a, in which representative images are shown on the left, and the percentages of CD8a-positive areas within the tumor region were calculated and are shown on the right (means ± s.e.m., *n* represents the number of mice, and are indicated in each panel; *P* values were calculated by two-way ANOVA, followed by Tukey).

h Depletion of CD8⁺ T cells diminishes the aldometanib-improved median lifespan. Mice were transplanted into the left lobe with Hepa1-6 cells, treated with aldometanib, and depleted CD8⁺ T cells using neutralizing antibody (depicted in the upper panel). The lifespan of mice was determined and is shown as Kaplan-Meier curves (see also statistical analyses in Supplementary Table 3).

Experiments in this figure were performed three times.
