# Supplementary material for: Glucose starvation mimetic aldometanib removes immune barriers permitting mice with hepatocellular carcinoma to live to normal ages
Source: Cell Res. 2025 Nov 25;35(12):934–53. doi: 10.1038/s41422-025-01195-4 (PMC12690099; doi:10.1038/s41422-025-01195-4)
Supplement: Supplementary file 11 — Supplementary information, Figure S11 [file 41422_2025_1195_MOESM11_ESM.pdf]

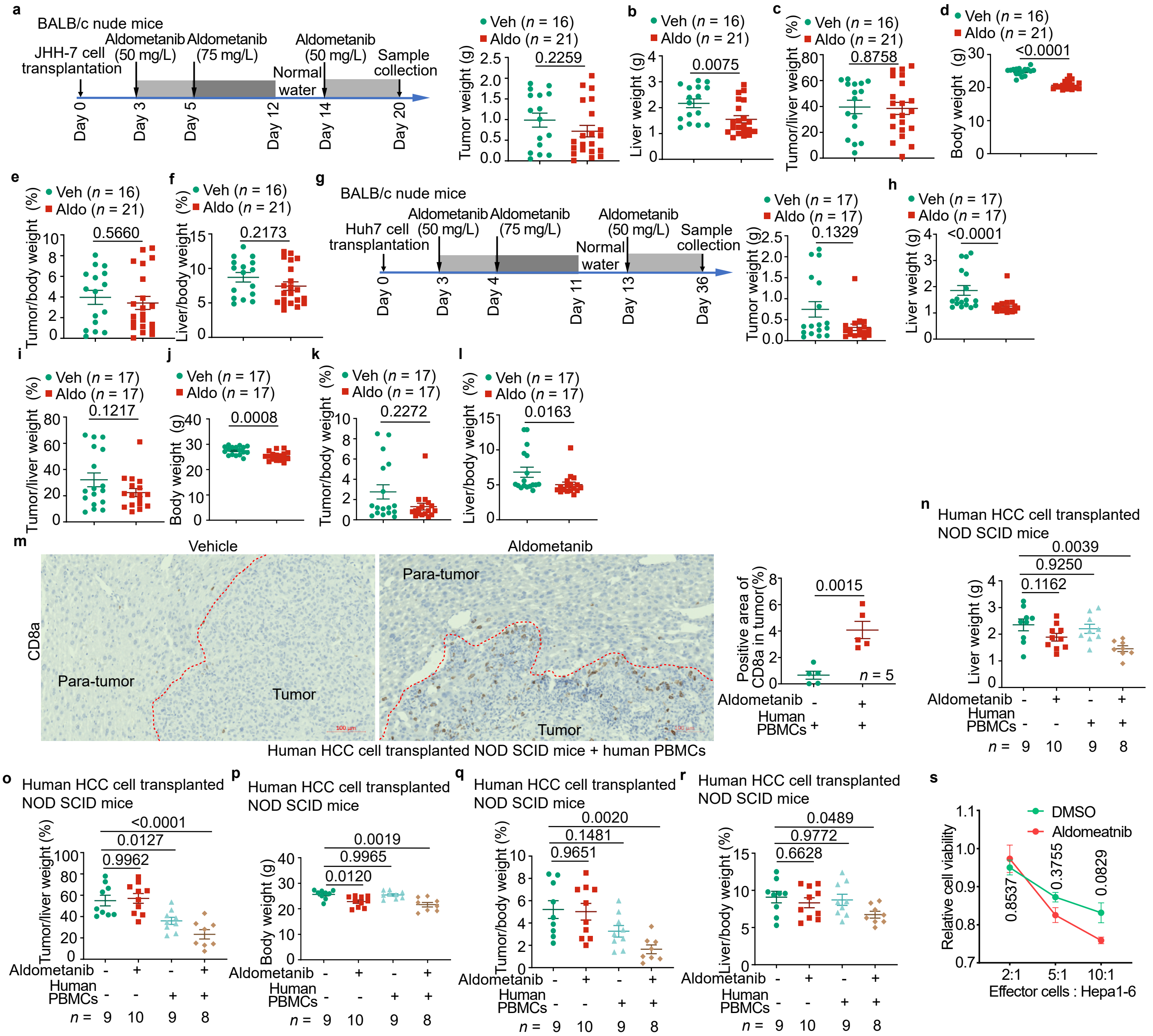

Fig. S11 Aldometanib fails to inhibit orthotopic allografts grown in the liver of nude mice lacking CD8⁺ T cells.

a-f BALB/c nude mice were transplanted with JHH-7 cells, followed by treatment of aldometanib starting from day 3 post-transplantation as described in **a**. HCC tissue samples were collected on day 20, followed by determination of the tumor weights (**a**), liver weights (**b**), tumor:liver weight ratios (**c**), body weights (**d**), tumor:body weight ratios (**e**), and liver:body weight ratios (**f**). Data are shown as means ± s.e.m., *n* represents the number of mice, and are labelled in each panel, with *P* values calculated by two-sided Student's *t*-test (**a-e**), or by Mann-Whitney test (**f**).

g-l BALB/c nude mice were transplanted Huh7 cells, followed by treatment of aldometanib starting day 3 post-transplantation as described in **g**. HCC tissue samples were collected on day 36, followed by determination of the tumor weights (**g**), liver weights (**h**), tumor:liver weight ratios (**i**), body weights (**j**), tumor:body weight ratios (**k**), and liver:body weight ratios (**l**). Data are shown as means ± s.e.m., *n* represents the number of mice, and are labelled in each panel, with *P* values calculated by two-sided Student's *t*-test (**j**), by two-sided Student's *t*-test with Welch's correction (**i**), or by Mann-Whitney test (others).

m-r Re-introduction of human PBMCs restores inhibitory effects of aldometanib on HCC in NOD-SCID mice. The NOD-SCID mice were intraperitoneally injected with human PBMCs, followed by transplantation with HCC cells from human HCC tissues, as in Fig. 4n. HCC tissue samples were collected, followed by determination of CD8⁺ T cell infiltration (**m**, by immunohistochemistry staining for CD8a; representative images are shown on the left, and the percentages of CD8a-positive areas within the tumor region were calculated and are shown on the right (means ± s.e.m., *n* represents the number of mice, and are labelled in each panel; *P* values were calculated by two-sided Student's *t*-test)), liver weights (**n**), tumor:liver weight ratios (**o**), body weights (**p**), tumor:body weight ratios (**q**), and liver:body weight ratios (**r**). Data are shown as means ± s.e.m., *n* represents the number of mice, and are labelled in each panel, with *P* values calculated by two-way ANOVA, followed by Tukey. The scale bars are 100 µm.

s Aldometanib does not increase the cytotoxicity of already activated CD8⁺ T cells against Hepa1-6 cells. Splenocytes were isolated from the spleens of wildtype C57BL/6J mice, followed by sequential incubation with anti-mouse CD3 and anti-mouse CD28 antibodies to activate CD8⁺ T cells inside. The activated CD8⁺ T cells were treated with aldometanib for 6 h, followed by co-culturing with Hepa1-6 cells at varying effector-to-target (E:T) ratios (0:1 (control), 2:1, 5:1, and 10:1) for another 48 h. The relative viability of the remaining Hepa1-6 cells was assessed using crystal violet staining. Data are shown as means ± s.e.m., *n* = 3 biological replicates, with *P* values calculated by two-way ANOVA, followed by Sidak's test.

Experiments in this figure were performed three times.
