# Supplementary material for: Glucose starvation mimetic aldometanib removes immune barriers permitting mice with hepatocellular carcinoma to live to normal ages
Source: Cell Res. 2025 Nov 25;35(12):934–53. doi: 10.1038/s41422-025-01195-4 (PMC12690099; doi:10.1038/s41422-025-01195-4)
Supplement: Supplementary file 12 — Supplementary information, Figure S12 [file 41422_2025_1195_MOESM12_ESM.pdf]

Supplementary information, Figure S12

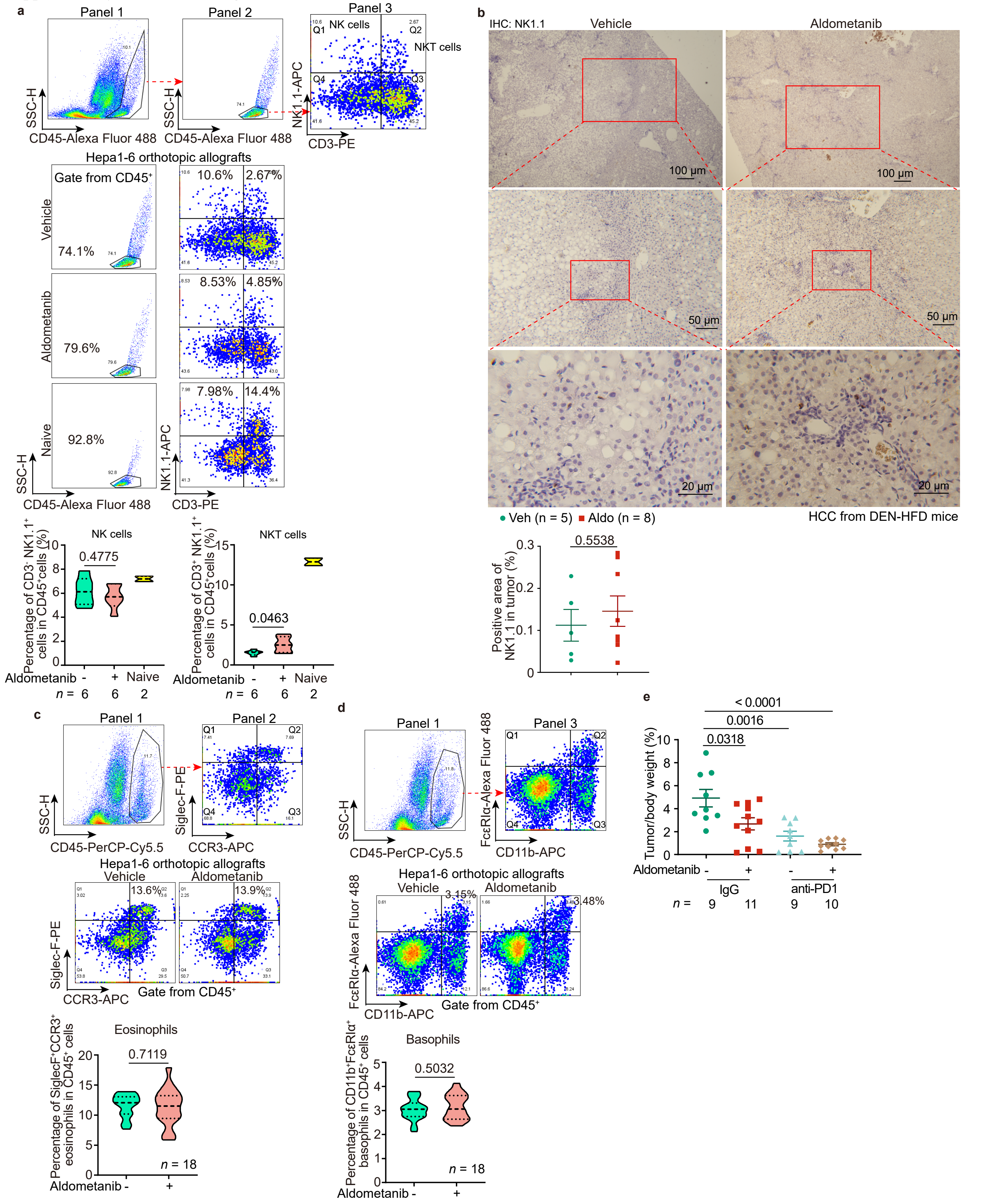

Fig. S12 Effects of aldometanib on other tumor-infiltrating lymphocytes than CD8⁺ T cells.

a, b Aldometanib promotes infiltration of NKT, but not NK cells, into HCC tissues. Experiments were performed as in Supplementary information, Fig S8b, except that NK and NKT cells were determined using both flow cytometry (**a**; with CD3-PE, NK1.1-APC, and CD45-Alexa Fluor 488 antibodies) and immunohistochemistry staining (**b**; with NK1.1 antibody; representative images are shown on the upper, and the percentages of NK1.1-positive area in the tumor were calculated and are shown on the lower panel as means ± s.e.m., *n* represents the number of mice, and are labelled in each panel; and *P* values were calculated by two-sided Student's *t*-test). Representative density plots of flow cytometry are shown on the middle panel of **a**, and the statistical analysis data on the lower panel of **a** (means ± s.e.m., *n* = 18 samples from 6 mice, with *P* values calculated by two-sided Student's *t*-test (the left panel), or by two-sided Student's *t*-test with Welch's correction (the right panel)). See also the gating strategies on the upper panel of **a**, where the Alexa Fluor 488-positive and SSC-H-low (panels 1 and 2) cells were selected. Among these selected cells, those that were positive for PE and APC (Q2 of panel 3) were identified as NKT cells, while those negative for PE but positive for APC (Q1 of panel 3) were NK cells.

c, d Aldometanib does not promote infiltration of eosinophils and basophils. Experiments were performed as in Supplementary information, Fig S8b except that eosinophils (stained with CD170-Siglec-F-PE, CCR3-APC, and CD45-PerCP-Cy5.5 antibodies) and basophils (labelled with FcεRIα-Alexa Fluor 488, CD11b-APC, and CD45-PerCP-Cy5.5 antibodies) were determined. Representative density plots are shown on the middle panel, and the statistical analysis data on the lower panel (means ± s.e.m., *n* = 18 samples from 6 mice, with *P* values calculated by two-sided Student's *t*-test). See also the gating strategies on the upper panel, where the PerCP-Cy5.5- (panel 1), APC- and PE-positive (panel 2) cells were identified as eosinophils, while the PerCP-Cy5.5- (panel 1), Alexa Fluor 488- and APC-positive (panel 3) cells were basophils.

e Aldometanib enhances the sensitivity of HCC to immunotherapy. Experiments were performed as in Fig. 4o, and the tumor:body weight ratios were determined. Data are means ± s.e.m., *n* represents the number of mice, with *P* values calculated by two-way ANOVA, followed by Tukey.

Experiments in this figure were performed three times.
