# Supplementary material for: Glucose starvation mimetic aldometanib removes immune barriers permitting mice with hepatocellular carcinoma to live to normal ages
Source: Cell Res. 2025 Nov 25;35(12):934–53. doi: 10.1038/s41422-025-01195-4 (PMC12690099; doi:10.1038/s41422-025-01195-4)
Supplement: Supplementary file 13 — Supplementary information, Figure S13 [file 41422_2025_1195_MOESM13_ESM.pdf]

Supplementary information, Figure S13

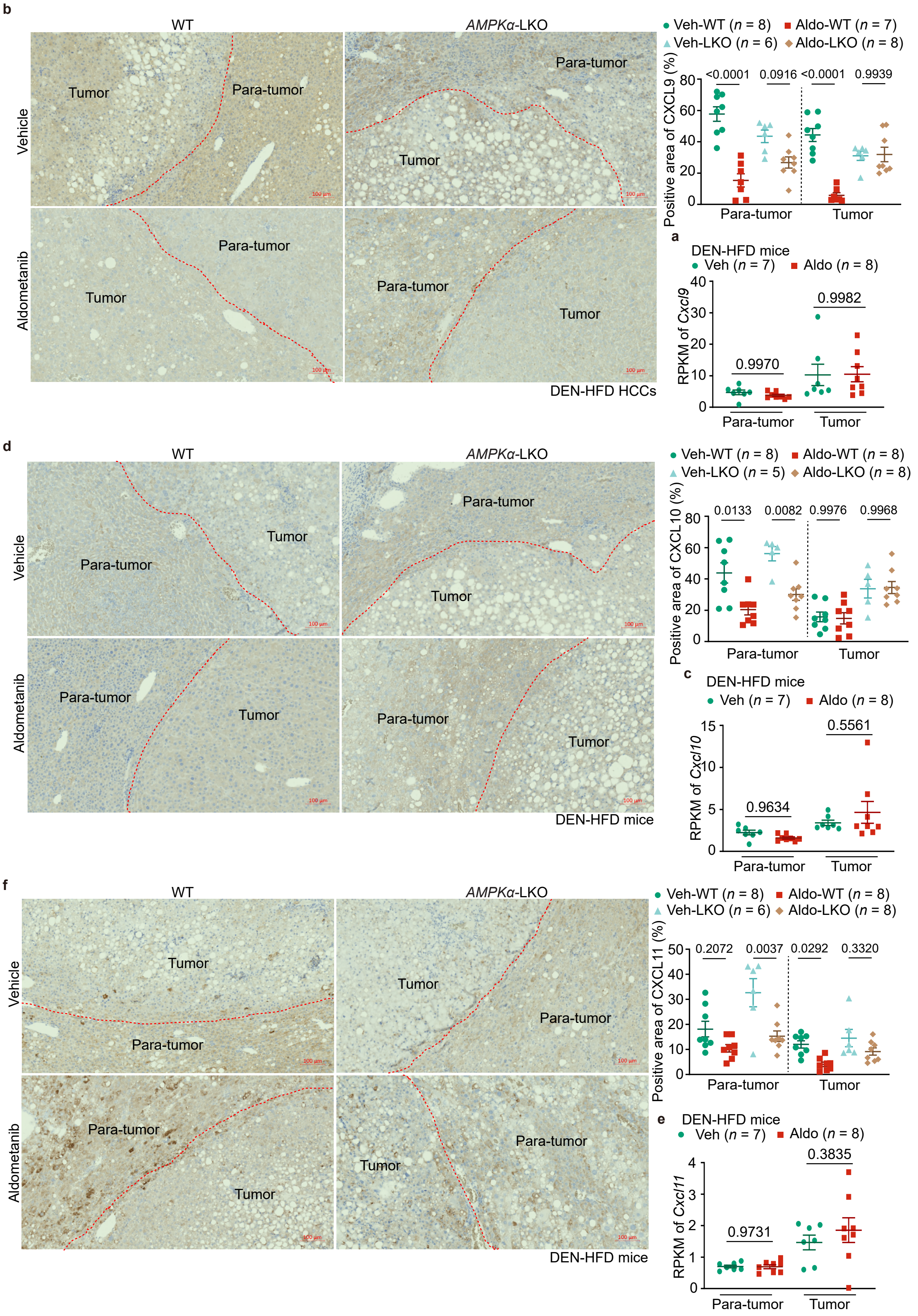

Fig. S13 Aldometanib does not promote secretion of chemokines in DEN-HFD HCCs.

a-f Mice induced to develop HCC using DEN and HFD to week 40 of age as in Supplementary information, Fig S8a, were treated with aldometanib as in Supplementary information, Fig S8a. The mRNA levels (**a**, **c**, **e**; by RNA sequencing, shown as means ± s.e.m., *n* represents the number of mice, and are labelled in each panel, with *P* values calculated by two-way ANOVA, followed by Tukey) and protein (**b**, **d**, **f**; by immunohistochemistry staining; representative images are shown on the left panels, and the percentages of CXCL9/10/11-positive area within the tumor area were calculated and are shown on the right panel as means ± s.e.m., *n* represents the number of mice, and are labelled in each panel; and *P* values were calculated by two-way ANOVA, followed by Tukey) levels of CXCL9, CXCL10, and CXCL11 in both tumor and para-tumor tissues were determined. The scale bars are 100 μm.

Experiments in this figure were performed three times.
