# Supplementary material for: Glucose starvation mimetic aldometanib removes immune barriers permitting mice with hepatocellular carcinoma to live to normal ages
Source: Cell Res. 2025 Nov 25;35(12):934–53. doi: 10.1038/s41422-025-01195-4 (PMC12690099; doi:10.1038/s41422-025-01195-4)
Supplement: Supplementary file 14 — Supplementary information, Figure S14 [file 41422_2025_1195_MOESM14_ESM.pdf]

Supplementary information, Figure S14

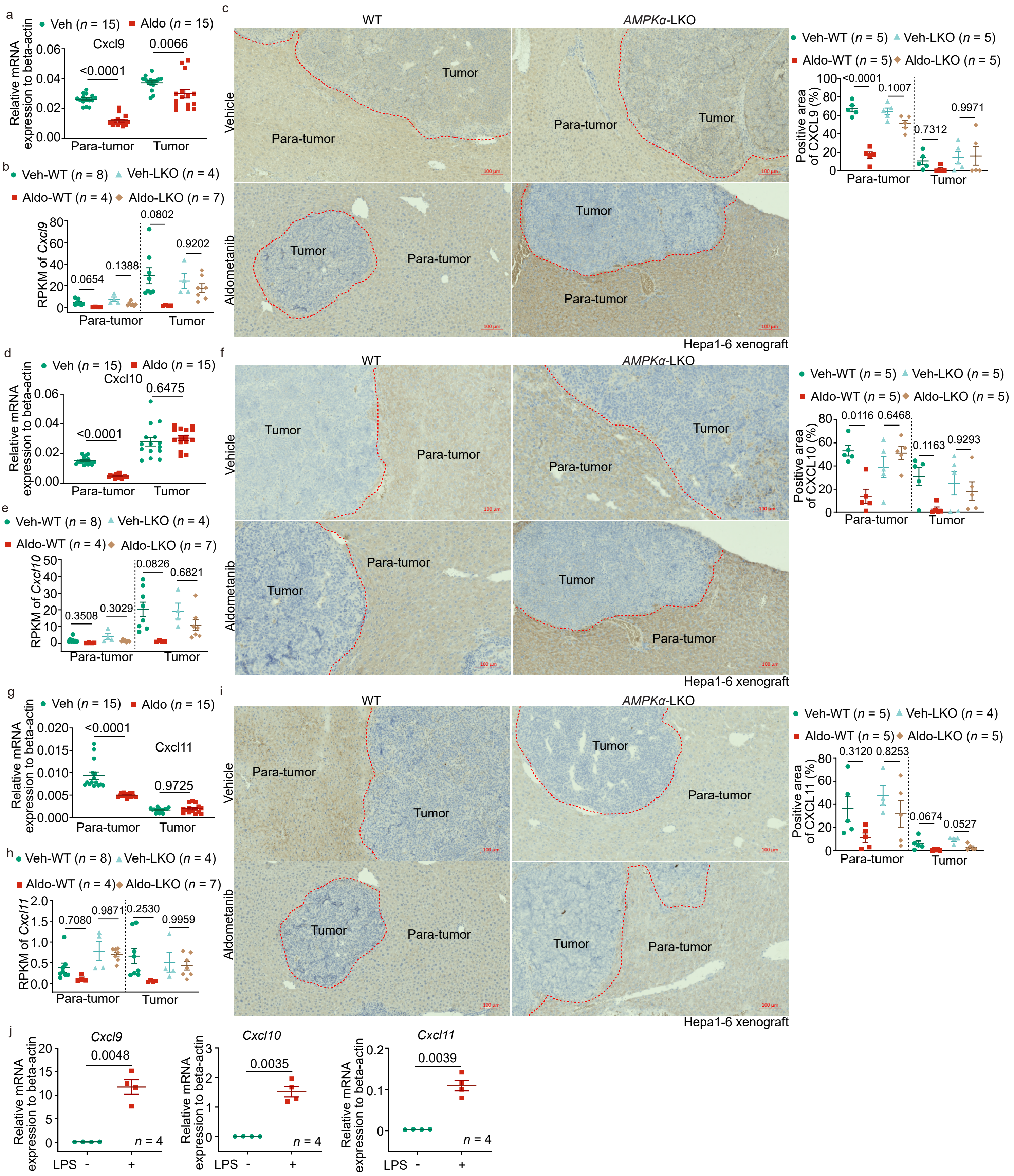

Fig. S14 Aldometanib does not promote the secretion of chemokines in Hepa1-6 orthotopic allografts.

a-i The hepa1-6-derived orthotopic allografts-bearing mice were treated with aldometanib as in Supplementary information, Fig S8b. The chemokine (*Cxcl9*, *Cxcl10*, and *Cxcl11*) mRNA levels, determined either by RT-PCR (**a**, **d**, **g**; shown as means ± s.e.m., n = 15 mice, with P values calculated by two-way ANOVA, followed by Tukey), or by RNA sequencing (**b**, **e**, **h**; shown as means ± s.e.m., n represents the number of mice, and are labelled in each panel, with P values calculated by two-way ANOVA, followed by Tukey), and the chemokine protein levels determined by immunohistochemistry staining (**c**, **f**, **i**) in both tumor and para-tumor tissues were shown (representative images are shown on the left panels, and the percentages of CXCL9/10/11-positive area within the tumor were calculated and are shown on the right panel as means ± s.e.m., n represents the number of mice, and are indicated in each panel; and P values were calculated by two-way ANOVA, followed by Tukey). The scale bars are 100 µm.

j LPS stimulates secretion of chemokines in the liver. Wildtype C57BL/6J mice, aged 8 weeks, were intraperitoneally injected with 10 mg/kg LPS (dissolved in PBS). At 6 h after the injection, the mice were euthanized, and liver tissues were collected, followed by determination of the mRNA levels of *Cxcl9*, *Cxcl10*, and *Cxcl11*. Data are shown as shown as means ± s.e.m., n = 4 mice, with P values calculated by two-sided Student's t-test with Welch's correction.

Experiments in this figure were performed three times.
