# Supplementary material for: Glucose starvation mimetic aldometanib removes immune barriers permitting mice with hepatocellular carcinoma to live to normal ages
Source: Cell Res. 2025 Nov 25;35(12):934–53. doi: 10.1038/s41422-025-01195-4 (PMC12690099; doi:10.1038/s41422-025-01195-4)
Supplement: Supplementary file 15 — Supplementary information, Figure S15 [file 41422_2025_1195_MOESM15_ESM.pdf]

Supplementary information, Figure S15

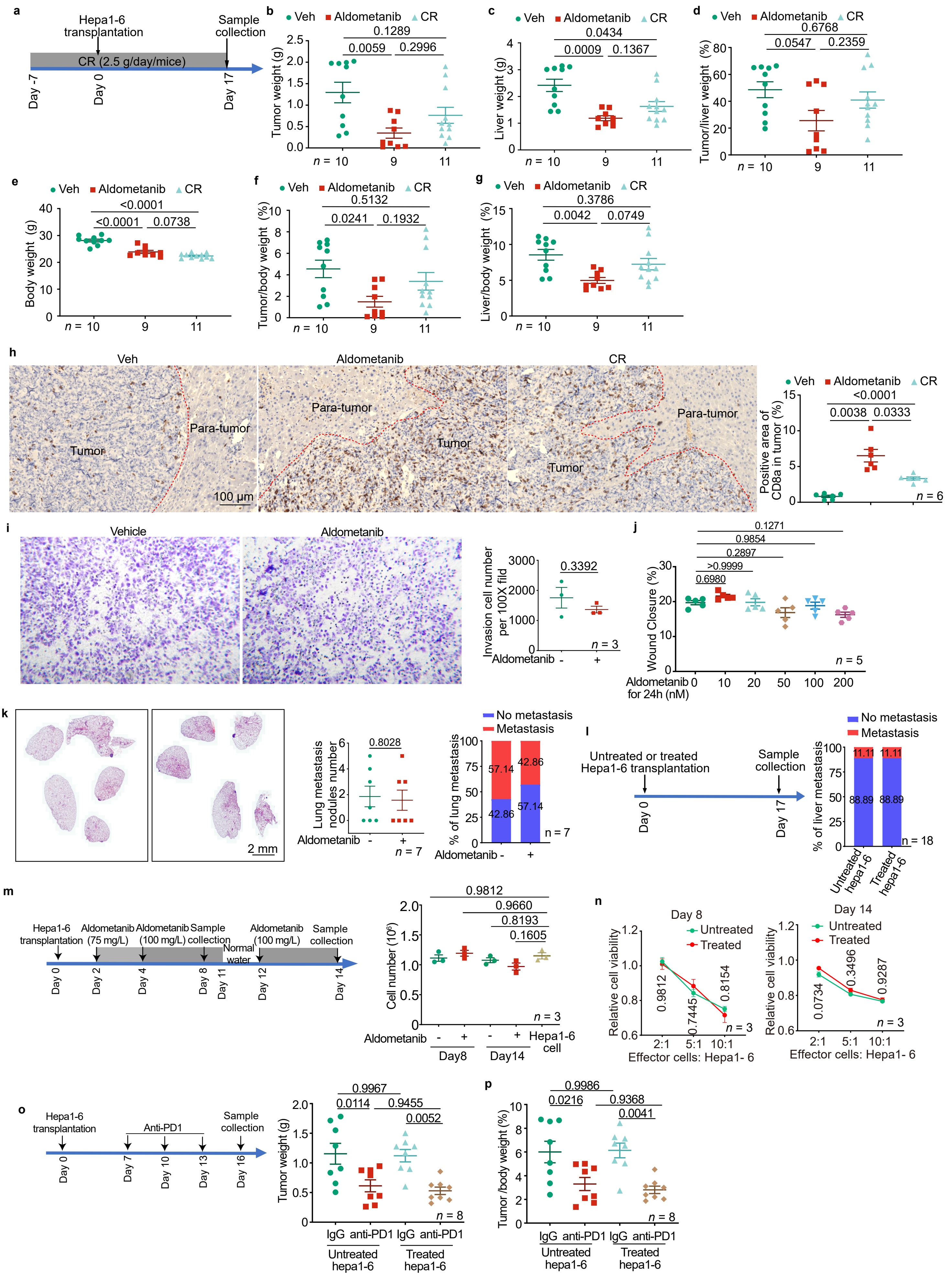

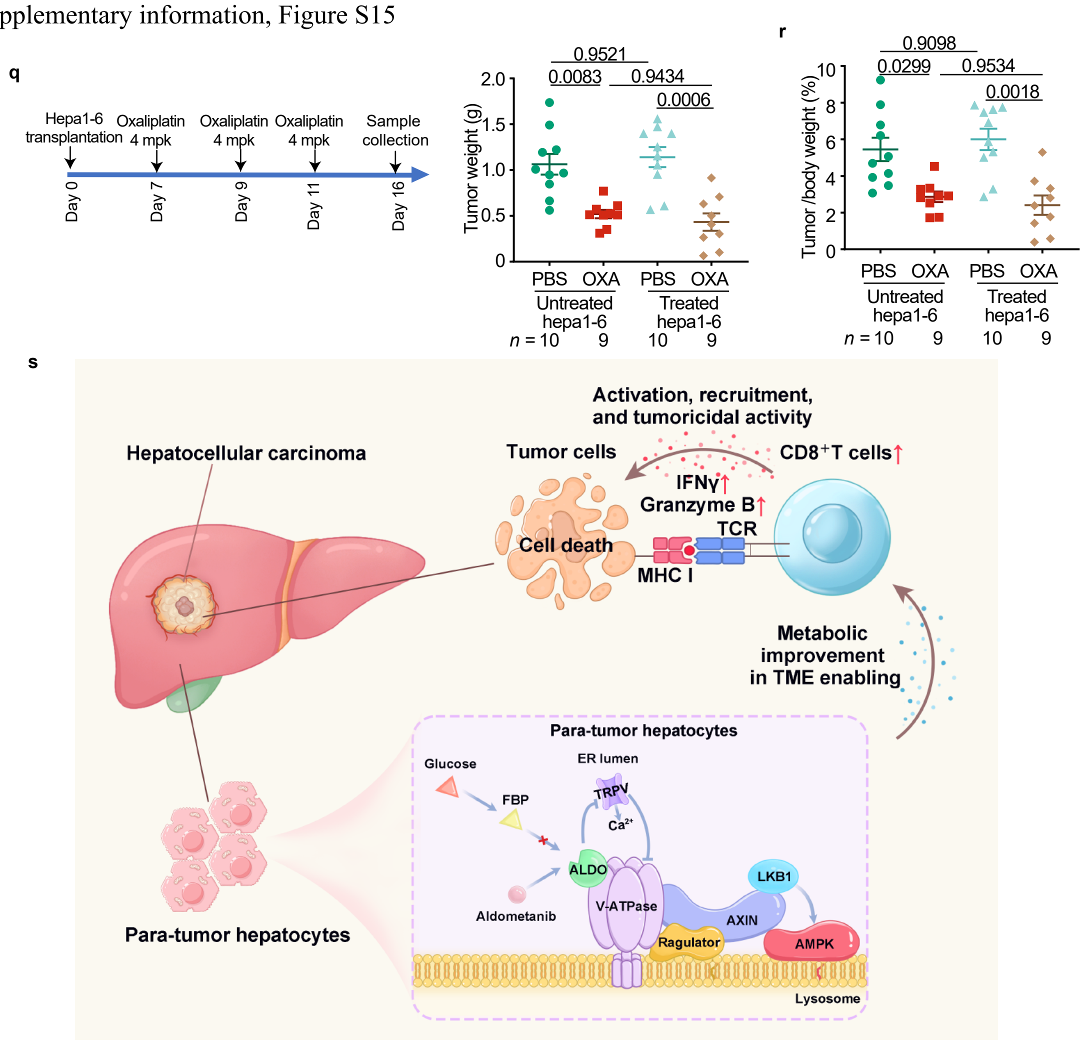

Fig. S15 Caloric restriction suppresses growth of HCC.

a-h Wildtype C57BL/6J mice, aged 8 weeks, were subjected to CR. Some 1 week after CR, Hepa1-6 cells were transplanted into the left liver lobes of mice, and the mice were further calorie-restricted for another 17 days (**a**). As a control, the aldometanib treatment group was set up as in Fig. 1f. The tumor weights (**b**), liver weights (**c**), tumor:liver weights (**d**), body weights (**e**), tumor:body weights (**f**), liver:body weights (**g**), and the infiltration of CD8$^+$ T (**h**), by immunohistochemistry staining for CD8a; representative images are shown on the left panels, and the percentages of CD8a-positive areas within the tumor region were calculated and are shown on the right panel as means ± s.e.m., *n* represents the number of mice, and are labelled in each panel; and *P* values were calculated by one-way ANOVA, followed by Tukey (**b**, **d**, **e**, **f**, **g**), or by Brown-Forsythe ANOVA test, followed by Dunnett's test (**c**, **h**). The scale bars are 100 µm.

i, j Aldometanib does not affect the invasiveness or migration of Hepa1-6 cells in vitro. Hepa1-6 cells were allowed to migrate through Matrigel in a Transwell insert for 24 h in the presence of aldometanib to assess their invasiveness (**i**; representative images are shown on the left panel, and the statistical analysis data are shown on the right panel), or subjected to a scratch assay to evaluate the migration in the presence of aldometanib for 24 h (**j**). Statistical analysis data in these panels are means ± s.e.m. *n* represents biological replicates (**i**, **j**), and are labelled in each panel, with *P* values calculated by two-sided Student's *t*-test, (**i**), or by one-way ANOVA, followed by Tukey (**j**).

k Aldometanib does not influence the lung metastasis ability of Hepa1-6 cells. Wildtype C57BL/6J mice were transplanted with Hepa1-6 cells into the left liver lobes to develop solid tumors, followed by treatment with aldometanib (as described in Fig. 1f). At day 17, mice were euthanized, followed by determination of lung metastasis nodules number by H&E staining. Representative images are shown on the left panels, and the statistical analysis data are shown on the right panel (means ± s.e.m., *n* represents the number of mice, with *P* values calculated by two-sided Student's *t*-test). The scale bars are 2 mm.

l Aldometanib does not increase intrahepatic metastasis ability of Hepa1-6 cells from allografts. Wildtype C57BL/6J mice were transplanted with Hepa1-6 cells into the left liver lobes to develop solid tumors, after which they received treatment with aldometanib (as described in Fig. 1f). On day 17, the mice were euthanized, and residual Hepa1-6 cells from the allografts were isolated. These cells were then transplanted again into the left liver lobes of new wildtype C57BL/6J mice. After 17 days, these mice were also euthanized, and intrahepatic metastasis was evaluated using H&E staining. Statistical analysis data are shown in **l**, and *n* represents the number of mice.

m Aldometanib does not increase the proliferation rate of Hepa1-6 cells from allografts. Wildtype C57BL/6 mice were transplanted with Hepa1-6 cells into the left liver lobes, and then treated with aldometanib (as in Fig. 1f). On days 8 and 14, the mice were euthanized, and residual Hepa1-6 cells from the allografts were isolated. The number of cells was determined before and after 24 h of culturing. Data are shown as means ± s.e.m., with *P* values calculated by two-way ANOVA, followed by Tukey's test.

n Aldometanib does not decrease the susceptibility of Hepa1-6 cells derived from allografts to cytotoxicity from CD8$^+$ T cells in vitro. Wild-type C57BL/6 mice were transplanted with Hepa1-6 cells into the left liver lobes and subsequently treated with aldometanib (as shown in Fig. 1f). On days 8 and 14, the mice were euthanized, and the residual Hepa1-6 cells from the allografts were isolated. These cells were then co-cultured with CD8$^+$ T cells, which had been pre-activated using anti-CD3 and anti-CD28 antibodies (as described in Fig. S11s), for 48 h. The viability of the remaining Hepa1-6 cells was assessed through crystal violet staining. Data are shown as means ± s.e.m., *n* = 3 biological replicates, with *P* values calculated by two-way ANOVA, followed by Sidak's test.

o-r Aldometanib does not decrease the sensitivity of Hepa1-6 cells derived from allografts to immunotherapy or chemotherapy. Wildtype C57BL/6 mice were transplanted Hepa1-6 cells into the left liver lobes to develop solid tumors, followed by treatment with aldometanib (as in Fig. 1f). On day 17, the mice were euthanized, and residual Hepa1-6 cells from the allografts were isolated. These cells were then transplanted again into the left liver lobes of new wildtype C57BL/6J mice. At day 7 post-transplantation, the mice received intraperitoneal injections of 50 µg of anti-PD-1 or 4 mg/kg of oxaliplatin every three days or every two days (also depicted in the left panels of **o** and **q**). On day 16 post-transplantation, the tumor weight (**o**), (**q**) and tumor:body weight ratios (**p**), (**r**) were determined. Data are shown as means ± s.e.m., *n* represents the number of mice, and are labelled in each panel, with *P* values calculated by two-way ANOVA, followed by Tukey.

s Schematic diagram showing how aldometanib inhibits HCC by activating lysosomal AMPK in the para-tumor tissues. Aldometanib binds aldolase to prevent the binding of FBP, which mimics a low-glucose state and leads to the activation of the lysosomally localized AMPK in the para-tumor hepatocytes. By activating AMPK, aldometanib "clears" the way for the mobilization of immune cells, particularly the CD8$^+$ T cells, to induce the cytotoxicity to the HCC tissues.

Experiments in this figure were performed three times.
