# Supplementary material for: Glucose starvation mimetic aldometanib removes immune barriers permitting mice with hepatocellular carcinoma to live to normal ages
Source: Cell Res. 2025 Nov 25;35(12):934–53. doi: 10.1038/s41422-025-01195-4 (PMC12690099; doi:10.1038/s41422-025-01195-4)
Supplement: Supplementary file 16 — Supplementary information, Table S1 [file 41422_2025_1195_MOESM16_ESM.pdf]

Supplementary Table 1 | Summary of healthspan analysis in mice[a,b]

Genotypes/ treatments	Mean life span (days)			Median life span (days)						P-value Vs Vehicle control within each genotype (Mantel-CoX)
		95% confidence interval			95% confidence interval					
	Estimated life span ± s.e.m.	Lower bound	Upper bound	Estimated life span ± s.e.m.	Lower bound	Upper bound	N[c]	N[d]	N[e]	
				Fig. 1a						
Vehicle	563.970 ± 17.343	529.978	597.962	578.000 ± 27.561	523.980	632.020	33	20	53	N/A
Aldometanib	835.788 ± 40.509	756.389	915.186	805.000 ± 60.290	686.831	923.169	33	18	51	<0.001

[a]Independent repeats of each healthspan experiment were performed. Data from representative experiments are shown.
[b]Health span data sets within each panel of this table were done in parallel and statistical analyses was done within the data set.
[c]Number of mice scored (death events).
[d]Number of mice censored.
[e]Total number of mice.
