# Supplementary material for: Glucose starvation mimetic aldometanib removes immune barriers permitting mice with hepatocellular carcinoma to live to normal ages
Source: Cell Res. 2025 Nov 25;35(12):934–53. doi: 10.1038/s41422-025-01195-4 (PMC12690099; doi:10.1038/s41422-025-01195-4)
Supplement: Supplementary file 17 — Supplementary information, Table S2 [file 41422_2025_1195_MOESM17_ESM.pdf]

Supplementary Table 2 | Summary of lifespan analysis in mice[a,b]

	Mean life span (days)			Median life span (days)						
		95% confidence interval			95% confidence interval					
Genotypes/ treatments	Estimated life span ± s.e.m.	Lower bound	Upper bound	Estimated life span ± s.e.m.	Lower bound	Upper bound	N[c]	N[d]	N[e]	P-value Vs Vehicle control within each genotype (Mantel-CoX)
**Fig. 1g**										
Vehicle	26.600 ± 1.280	24.091	29.109	26.000 ± 0.730	24.596	27.431	20	0	20	N/A
Aldometanib	32.706 ± 1.146	30.405	34.953	33.000 ± 1.029	30.983	35.017	17	0	17	0.006

[a]Independent repeats of each lifespan experiment were performed. Data from representative experiments are shown.
[b]Lifespan data sets within each panel of this table were done in parallel and statistical analyses was done within the data set.
[c]Number of mice scored (death events).
[d]Number of mice censored.
[e]Total number of mice.
