# Supplementary material for: Glucose starvation mimetic aldometanib removes immune barriers permitting mice with hepatocellular carcinoma to live to normal ages
Source: Cell Res. 2025 Nov 25;35(12):934–53. doi: 10.1038/s41422-025-01195-4 (PMC12690099; doi:10.1038/s41422-025-01195-4)
Supplement: Supplementary file 18 — Supplementary information, Table S3 [file 41422_2025_1195_MOESM18_ESM.pdf]

Supplementary Table 3 | Summary of lifespan analysis in mice[a,b]

Genotypes/ treatments	Mean life span (days)			Median life span (days)						P-value Vs Vehicle control within each genotype (Mantel-CoX)
		95% confidence interval			95% confidence interval					
	Estimated life span ± s.e.m.	Lower bound	Upper bound	Estimated life span ± s.e.m.	Lower bound	Upper bound	N[c]	N[d]	N[e]	
**Fig. S10h**										
Vehicle	31.700 ± 1.415	28.927	34.473	33.000 ± 0.775	31.482	34.518	10	0	10	N/A
Aldometanib + IgG	43.700 ± 1.866	40.043	47.357	43.000 ± 2.372	38.351	47.649	6	4	10	<0.001
Aldometanib + CD8Ab	33.900 ± 1.378	31.199	36.601	34.000 ± 1.054	31.934	36.066	10	0	10	0.101

Genotypes/ treatments	Mean life span (days)			Median life span (days)						P-value Vs Aldometanib + IgG within each genotype (Mantel-CoX)
		95% confidence interval			95% confidence interval					
	Estimated life span ± s.e.m.	Lower bound	Upper bound	Estimated life span ± s.e.m.	Lower bound	Upper bound	N[c]	N[d]	N[e]	
**Fig. S10h**										
Vehicle	31.700 ± 1.415	28.927	34.473	33.000 ± 0.775	31.482	34.518	10	0	10	<0.001
Aldometanib + IgG	43.700 ± 1.866	40.043	47.357	43.000 ± 2.372	38.351	47.649	6	4	10	N/A
Aldometanib + CD8Ab	33.900 ± 1.378	31.199	36.601	34.000 ± 1.054	31.934	36.066	10	0	10	<0.001

[a]Independent repeats of each lifespan experiment were performed. Data from representative experiments are shown.
[b]Lifespan data sets within each panel of this table were done in parallel and statistical analyses was done within the data set.
[c]Number of mice scored (death events).
[d]Number of mice censored.
[e]Total number of mice.
