# Supplementary material for: Glucose starvation mimetic aldometanib removes immune barriers permitting mice with hepatocellular carcinoma to live to normal ages
Source: Cell Res. 2025 Nov 25;35(12):934–53. doi: 10.1038/s41422-025-01195-4 (PMC12690099; doi:10.1038/s41422-025-01195-4)

Supplementary Fig. 1 | Uncropped gels for Fig. 2 to 3 and Extended Data Fig. 1 to 6. After electrophoretic transfer of proteins, the PVDF membranes were cut into strips containing groups of samples, followed by immunoblotting. Shown here are films that had been exposed and developed to the membrane strips. The Pierce™ Prestained Protein MW Marker (Cat. 26612, and 26616 ThermoFisher Scientific) was used as the protein markers.

Fig. 2a

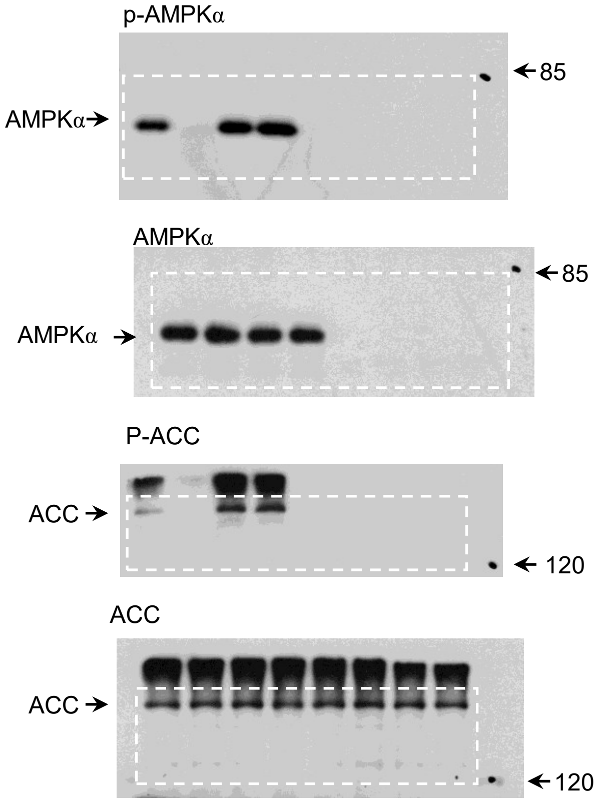

Fig. 2c

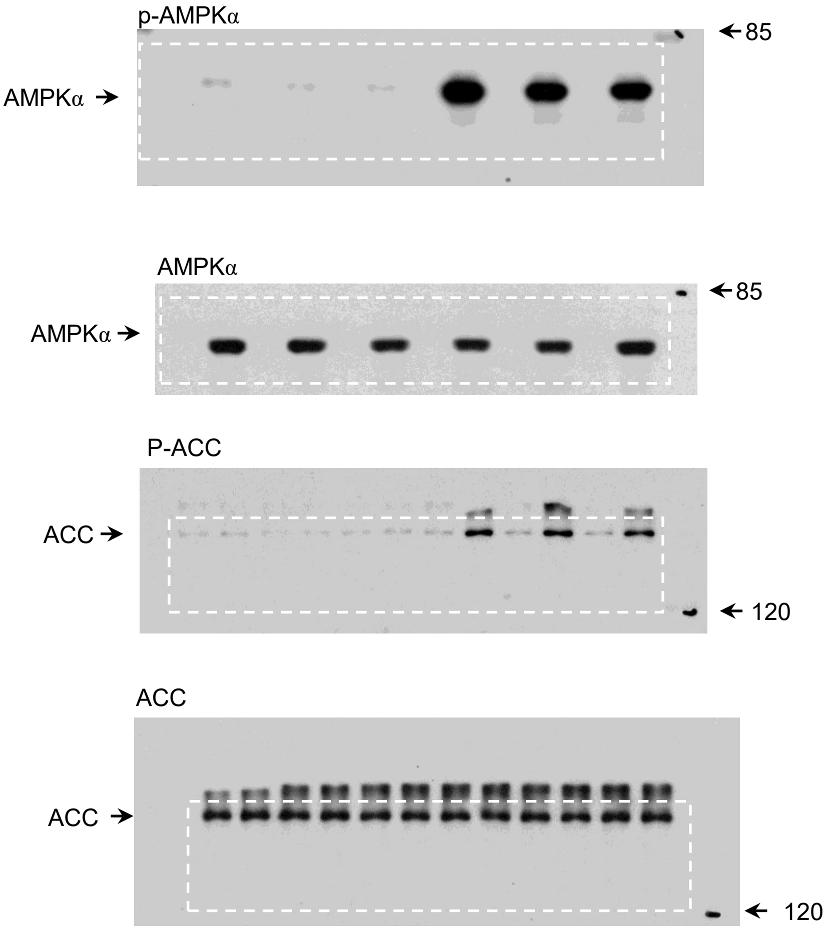

Fig. 2e

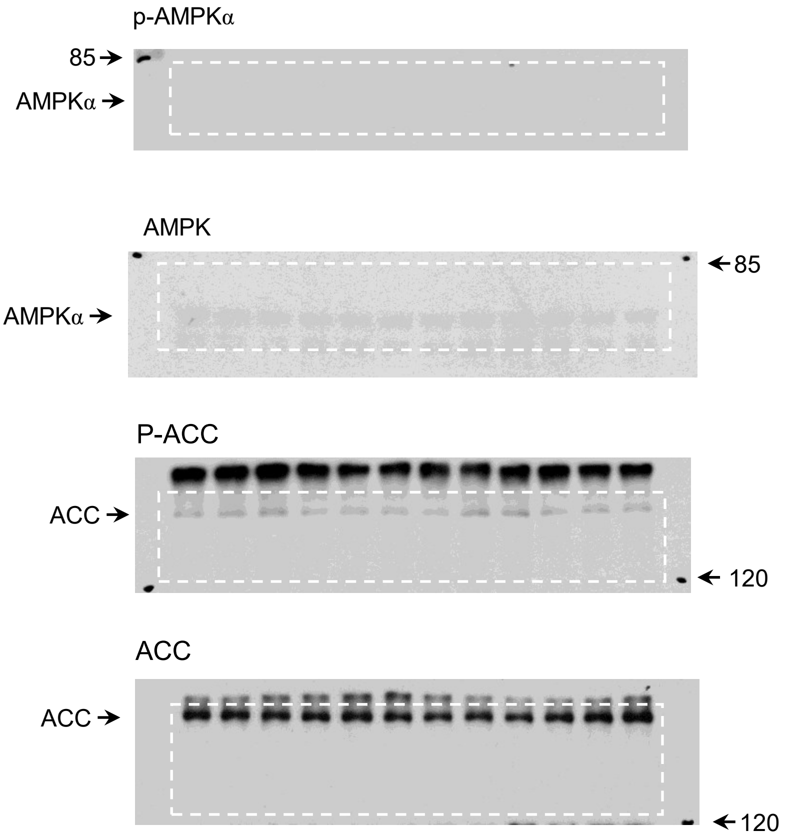
p-AMPKα
85
AMPKα
AMPK
85
AMPKα
P-ACC
ACC
120
ACC
ACC
120

Fig. 2g

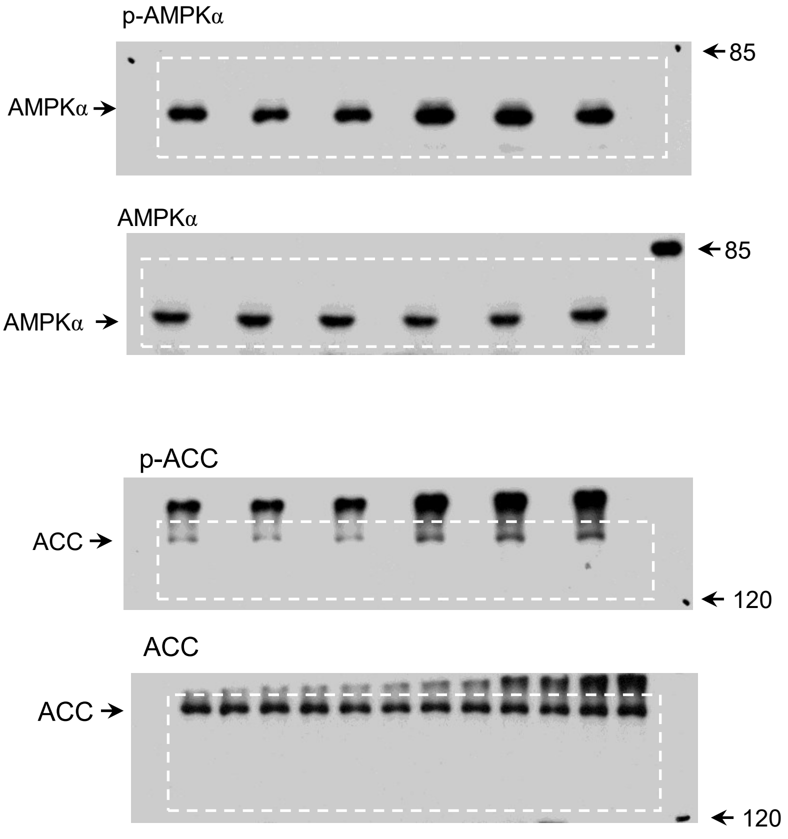
p-AMPKα
85
AMPKα
AMPKα
85
AMPKα
p-ACC
ACC
120
ACC
ACC
120

Fig. 3b

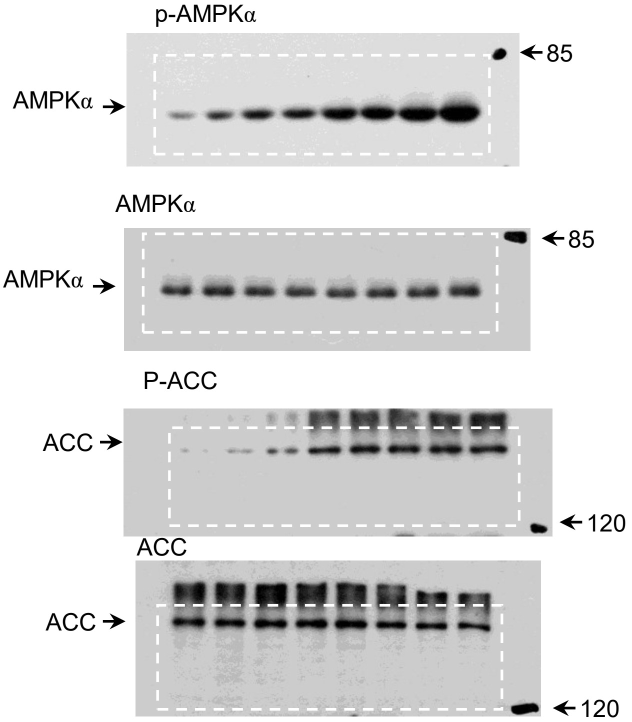

Fig. 3c

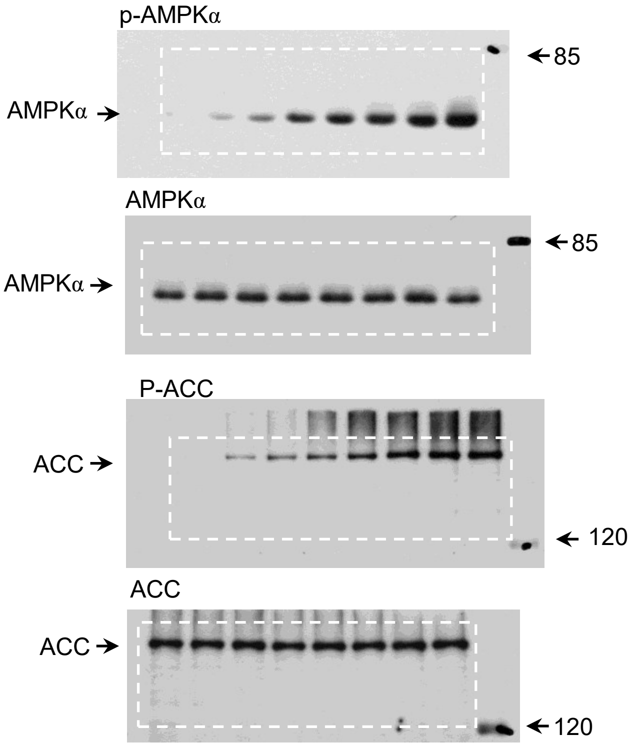

Fig. 3d

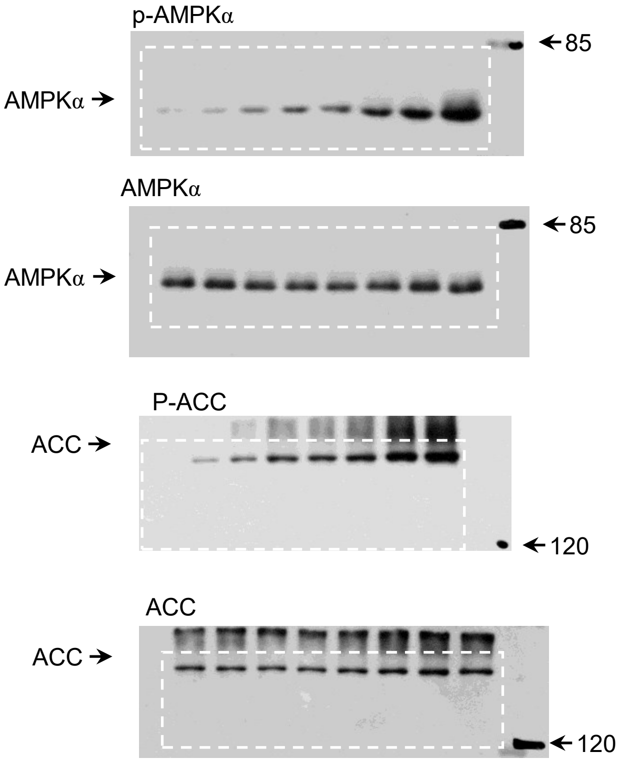

Fig. 3e

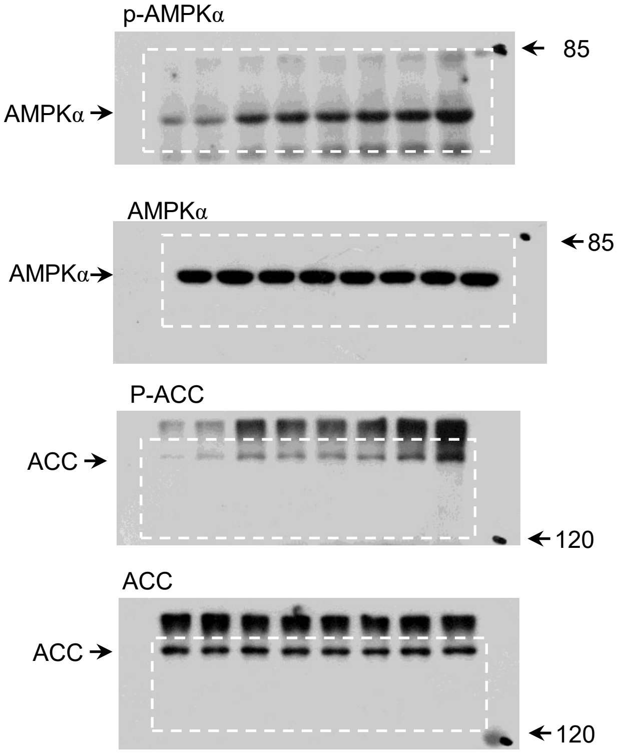

Fig. 3f

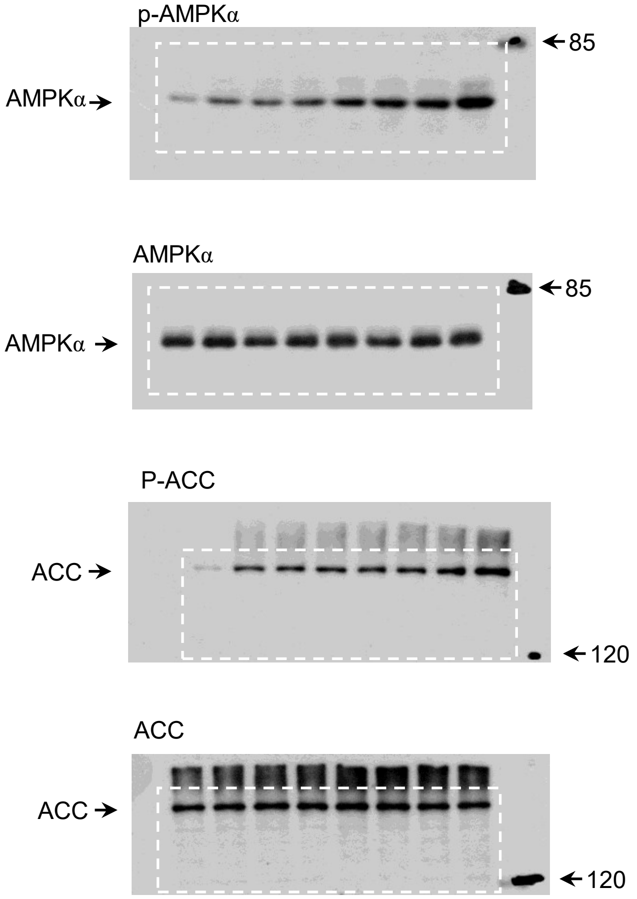

Fig. 3g

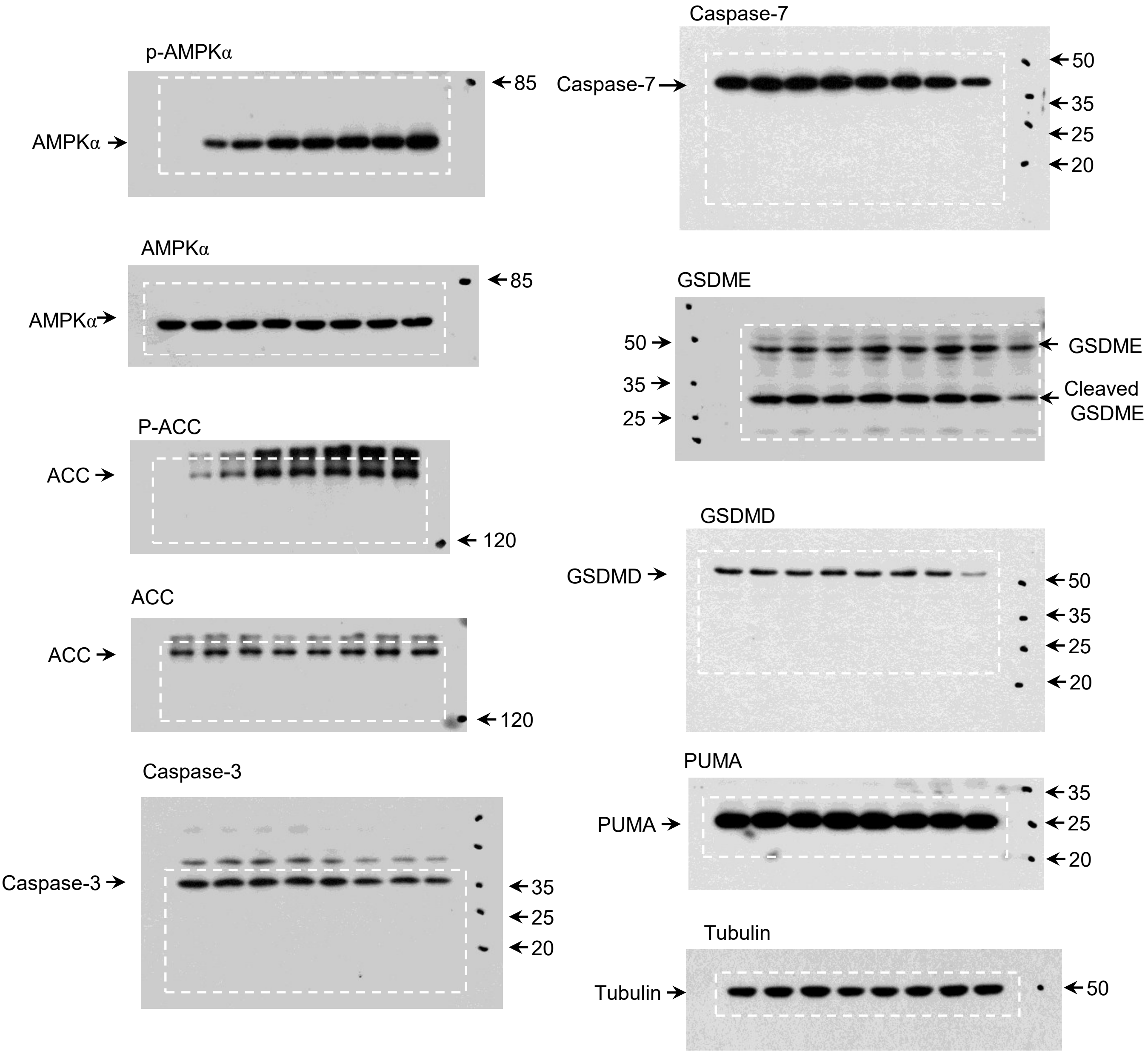
p-AMPKα
AMPKα
85
AMPKα
AMPKα
85
P-ACC
ACC
120
ACC
ACC
120
Caspase-3
Caspase-3
35
25
20
Caspase-7
Caspase-7
50
35
25
20
GSDME
50
35
25
GSDME
Cleaved GSDME
GSDMD
GSDMD
50
35
25
20
PUMA
PUMA
35
25
20
Tubulin
Tubulin
50

Fig. 3i

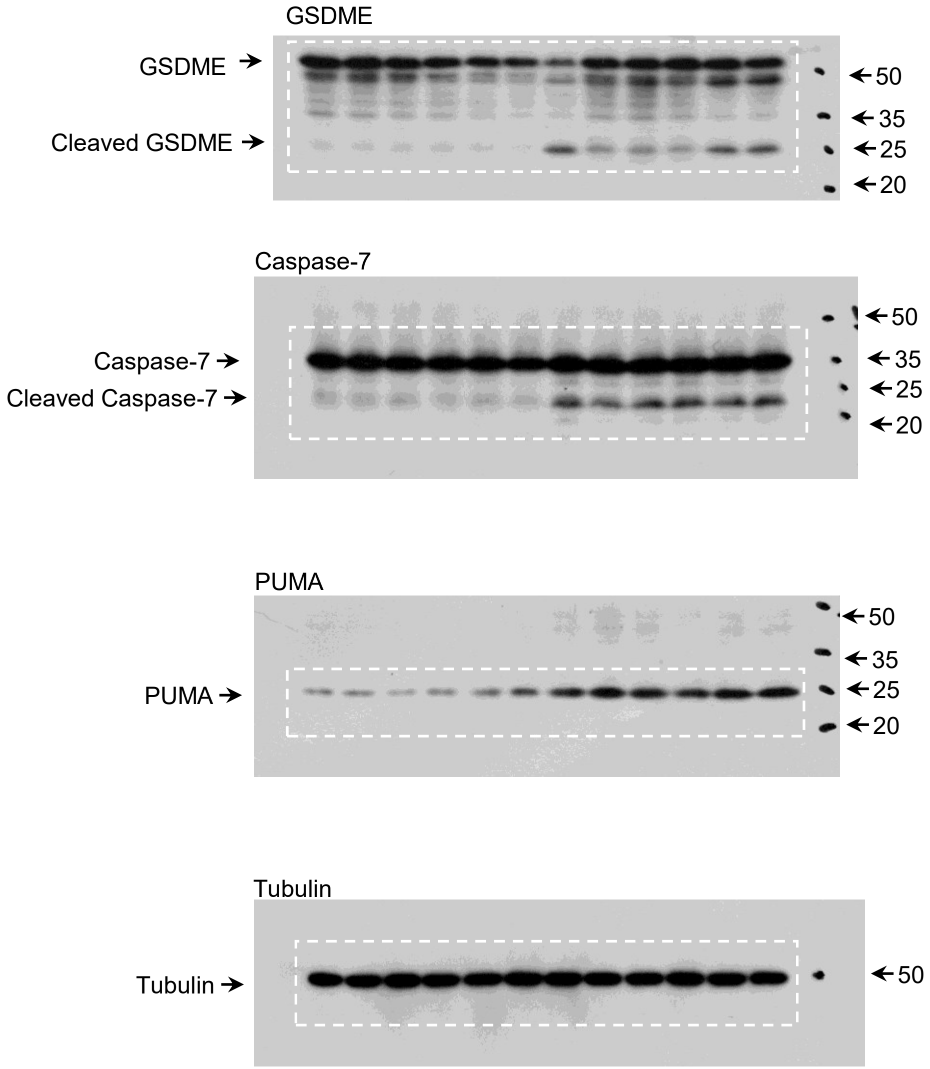
GSDME
GSDME
Cleaved GSDME
50
35
25
20
Caspase-7
Caspase-7
Cleaved Caspase-7
50
35
25
20
PUMA
PUMA
50
35
25
20
Tubulin
Tubulin
50

Fig. S1b
(representative images, repeat #1)

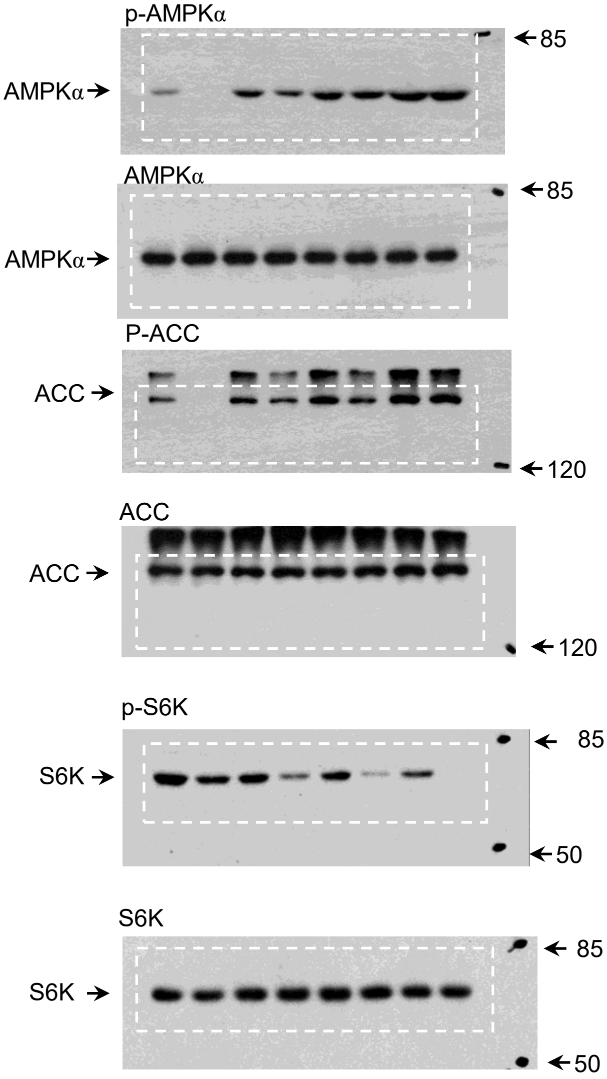

Fig. S1b (repeat #2)

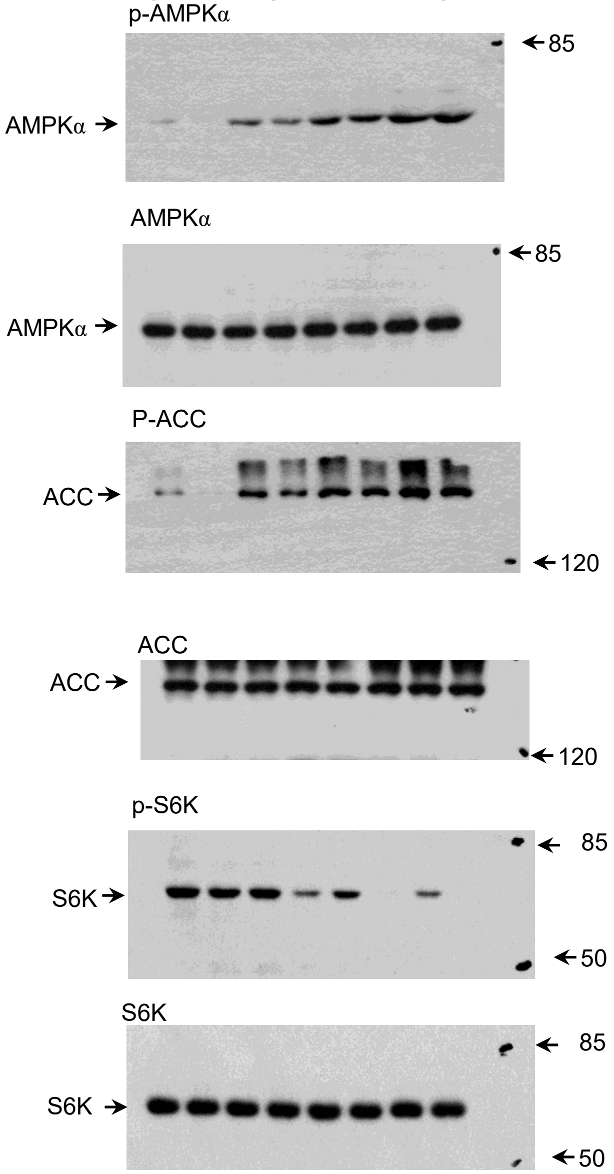

Fig. S1b (repeat #3)

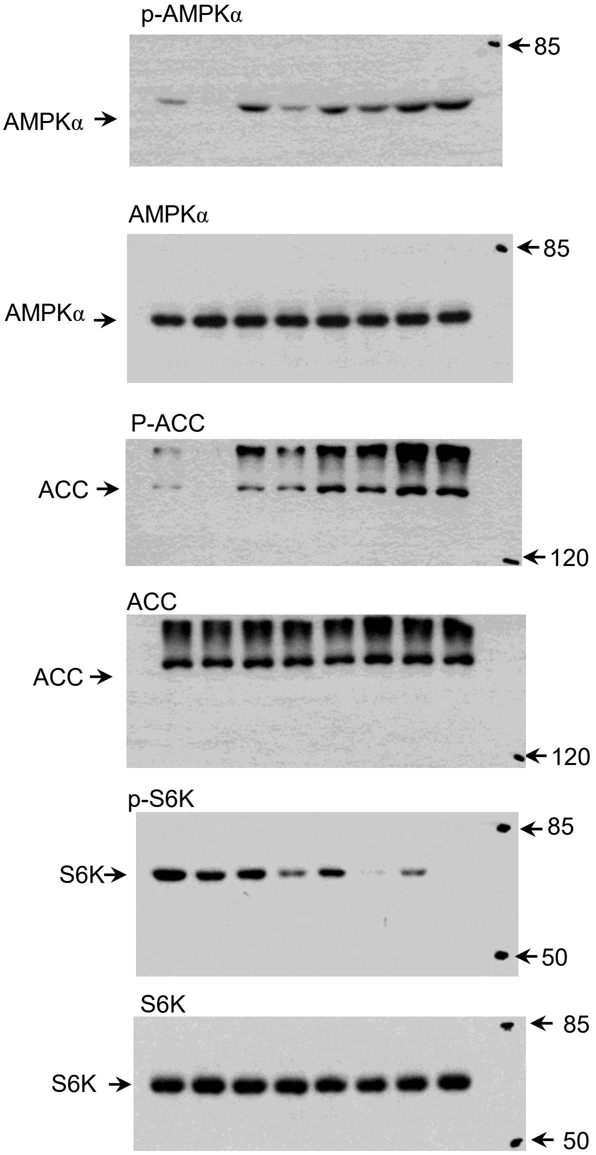

Fig. S1b (repeat #4)

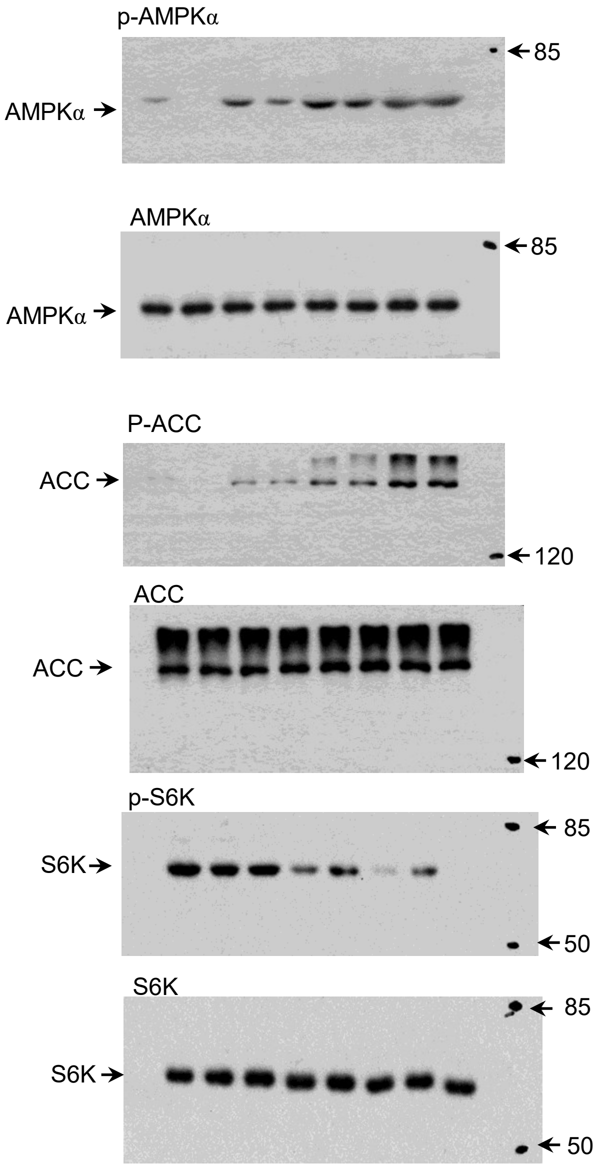

Fig. S1b (repeat #5)

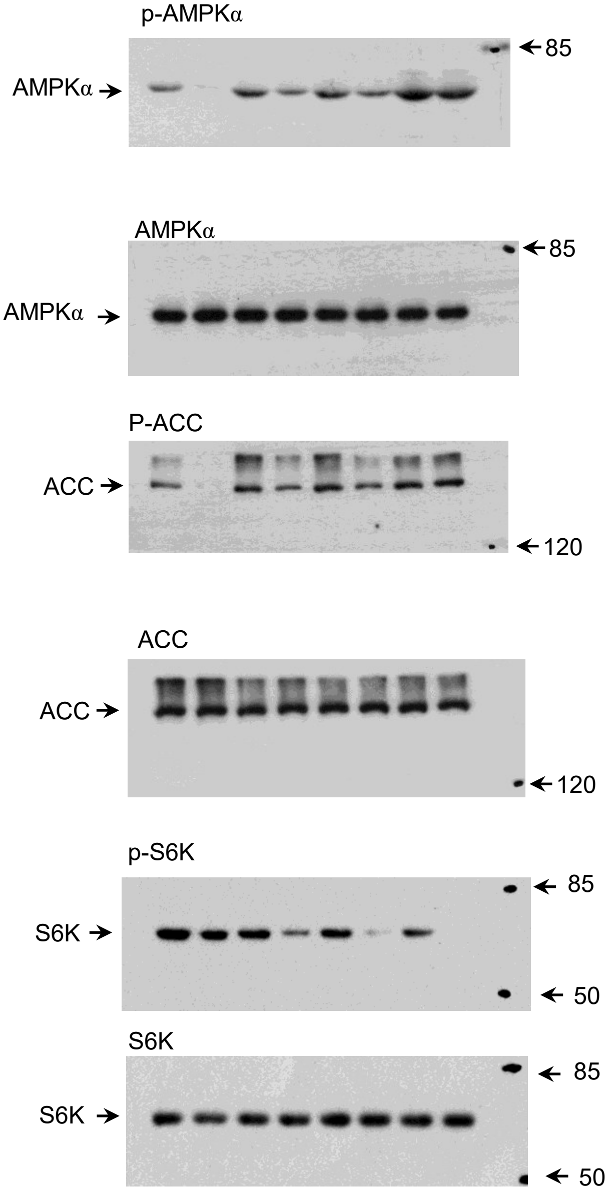

Fig. S4a

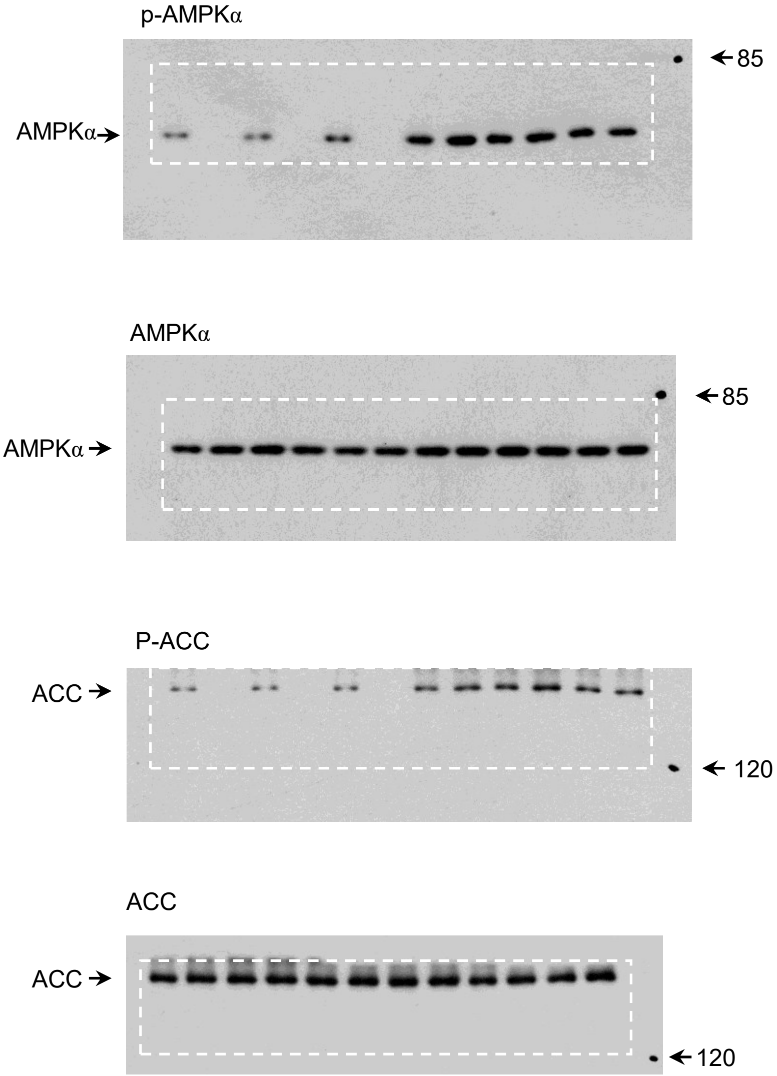

Fig. S5a

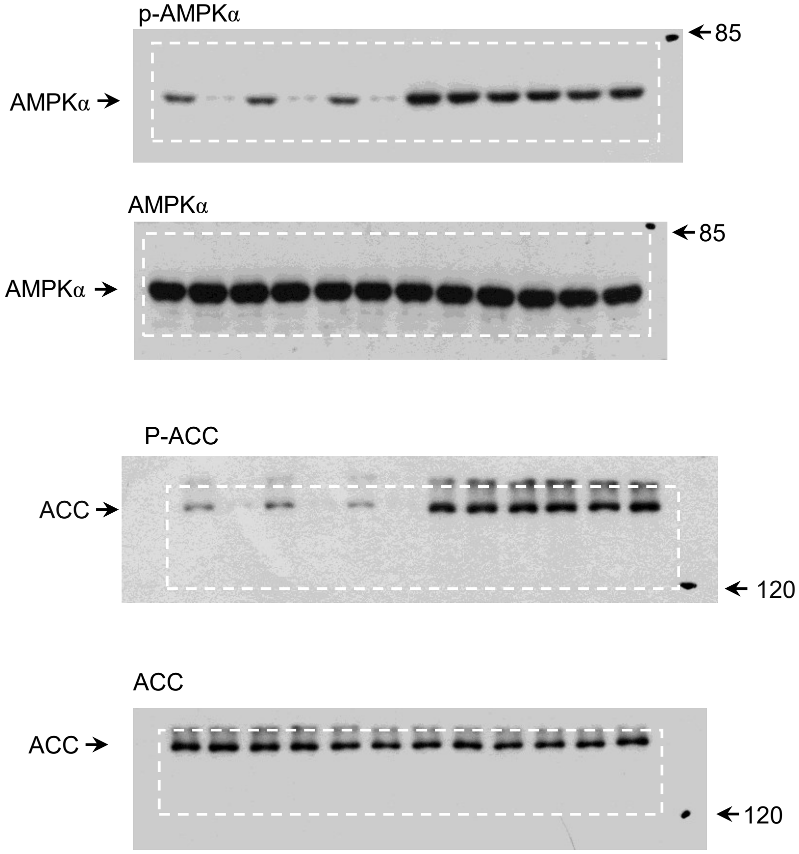

Fig. S5k

Fig. S5j

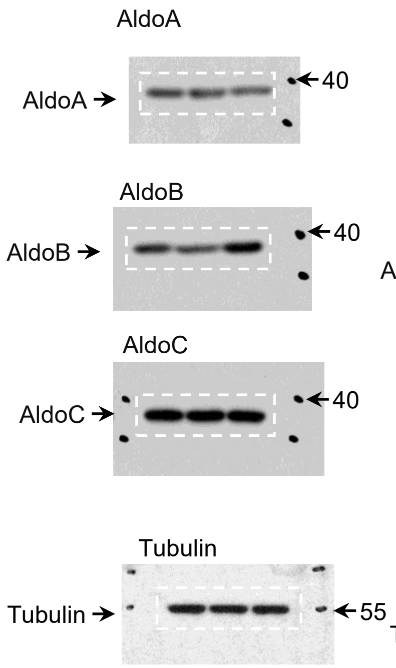

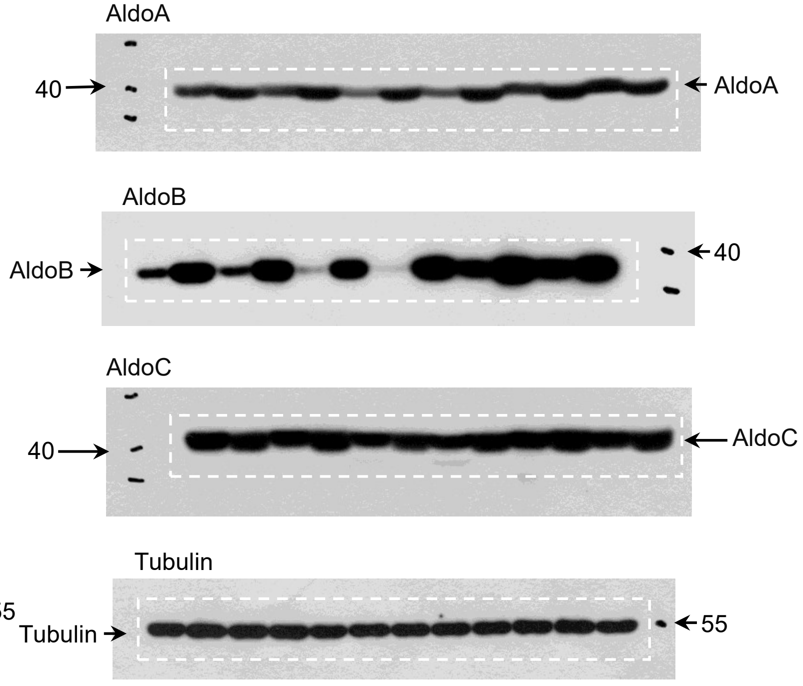

Fig. S5I

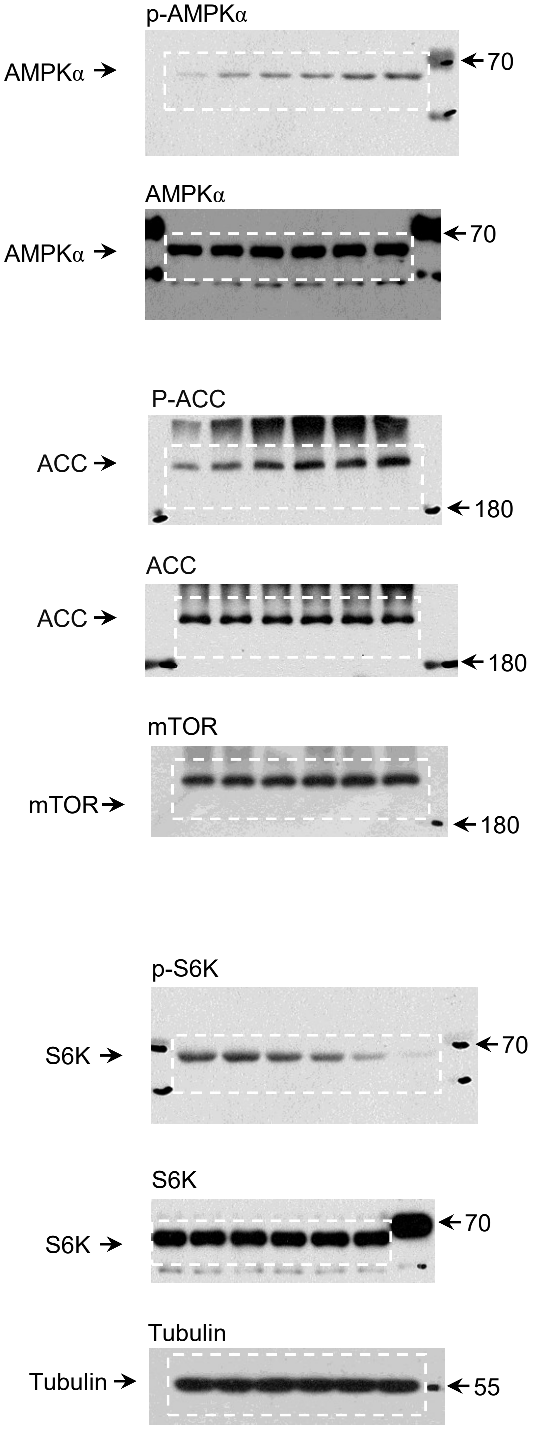

p-AMPKα
AMPKα →
← 70
AMPKα
AMPKα →
← 70
P-ACC
ACC →
← 180
ACC
ACC →
← 180
mTOR
mTOR→
← 180
p-S6K
S6K →
← 70
S6K
S6K →
← 70
Tubulin
Tubulin →
← 55

Fig. S5m

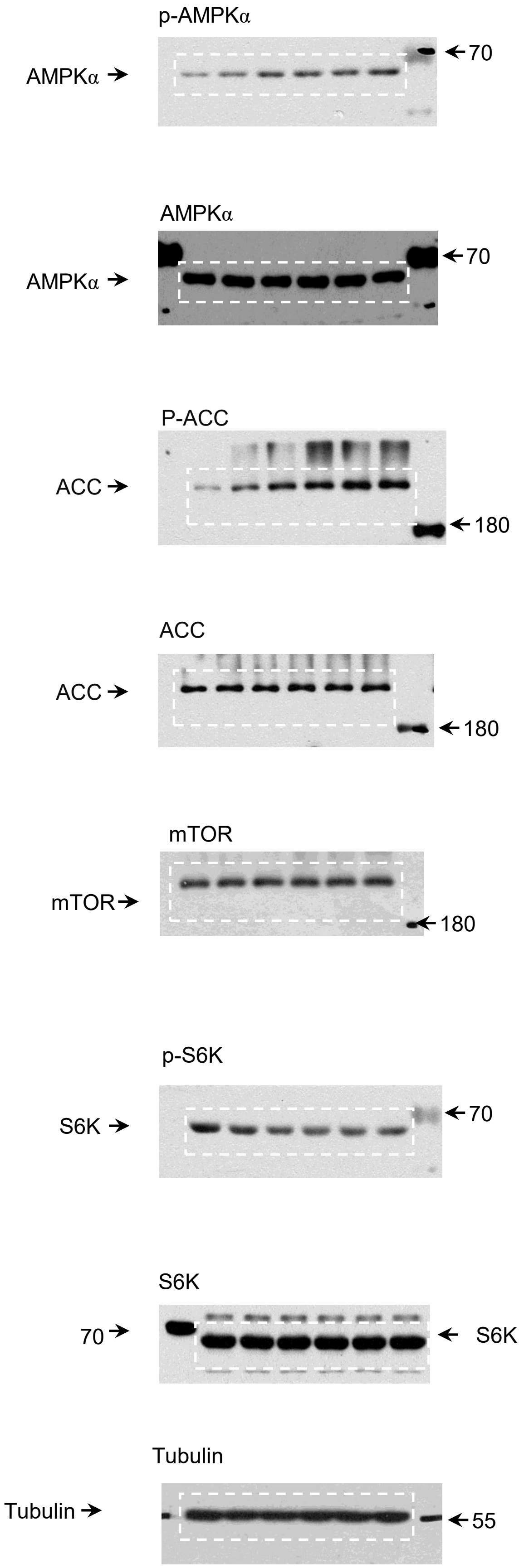
p-AMPKα
AMPKα
70
AMPKα
AMPKα
70
P-ACC
ACC
180
ACC
ACC
180
mTOR
mTOR
180
p-S6K
S6K
70
S6K
70
S6K
Tubulin
Tubulin
55

Fig. S5n

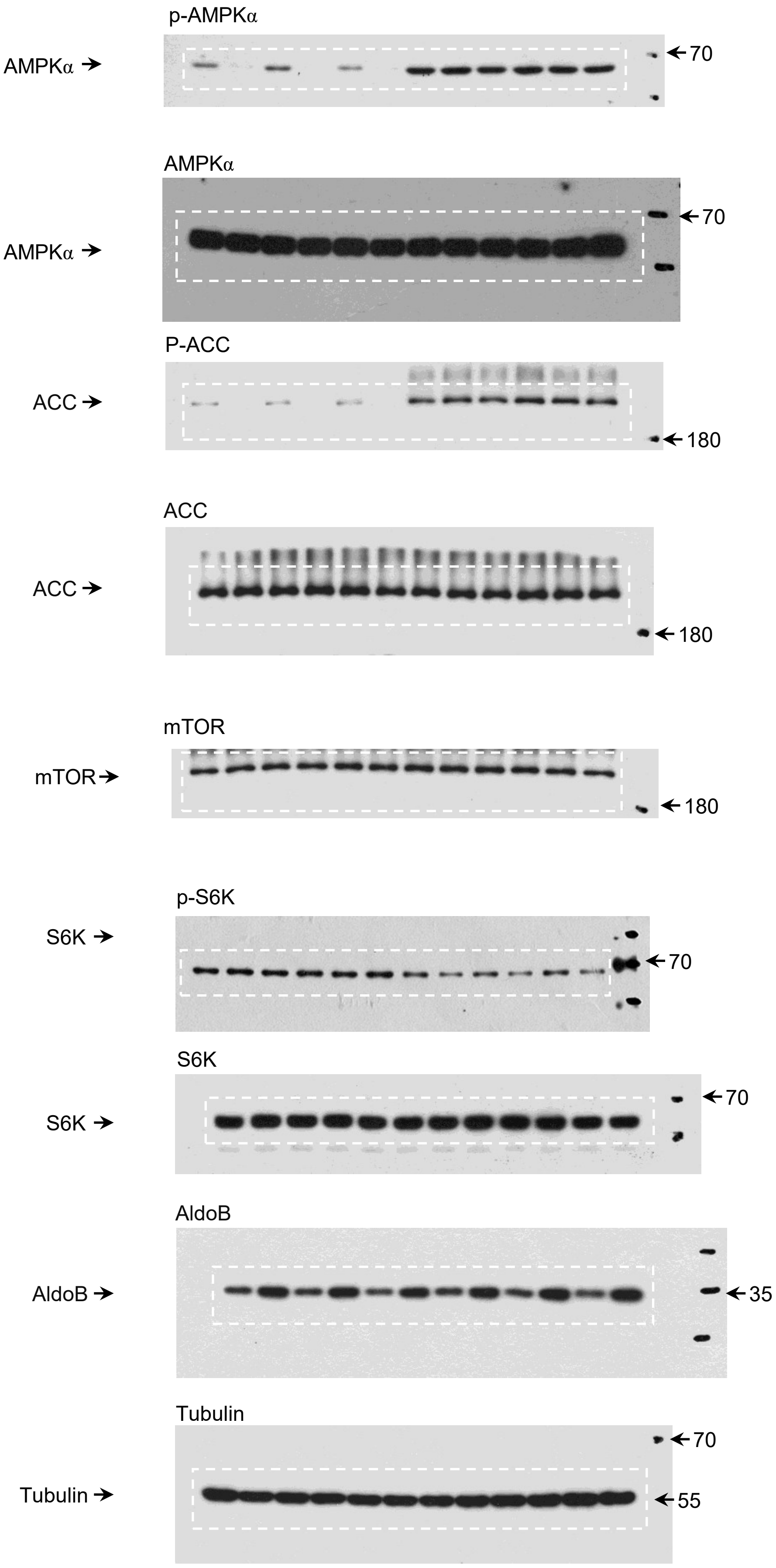
p-AMPKα
AMPKα
70
AMPKα
AMPKα
70
P-ACC
ACC
180
ACC
ACC
180
mTOR
mTOR
180
p-S6K
S6K
70
S6K
S6K
70
AldoB
AldoB
35
Tubulin
Tubulin
70
55

Fig. S6g

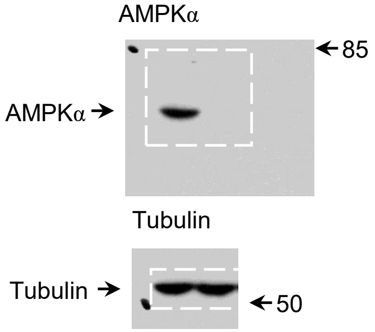

Determination of CD8a in Hepa1-6 allografts

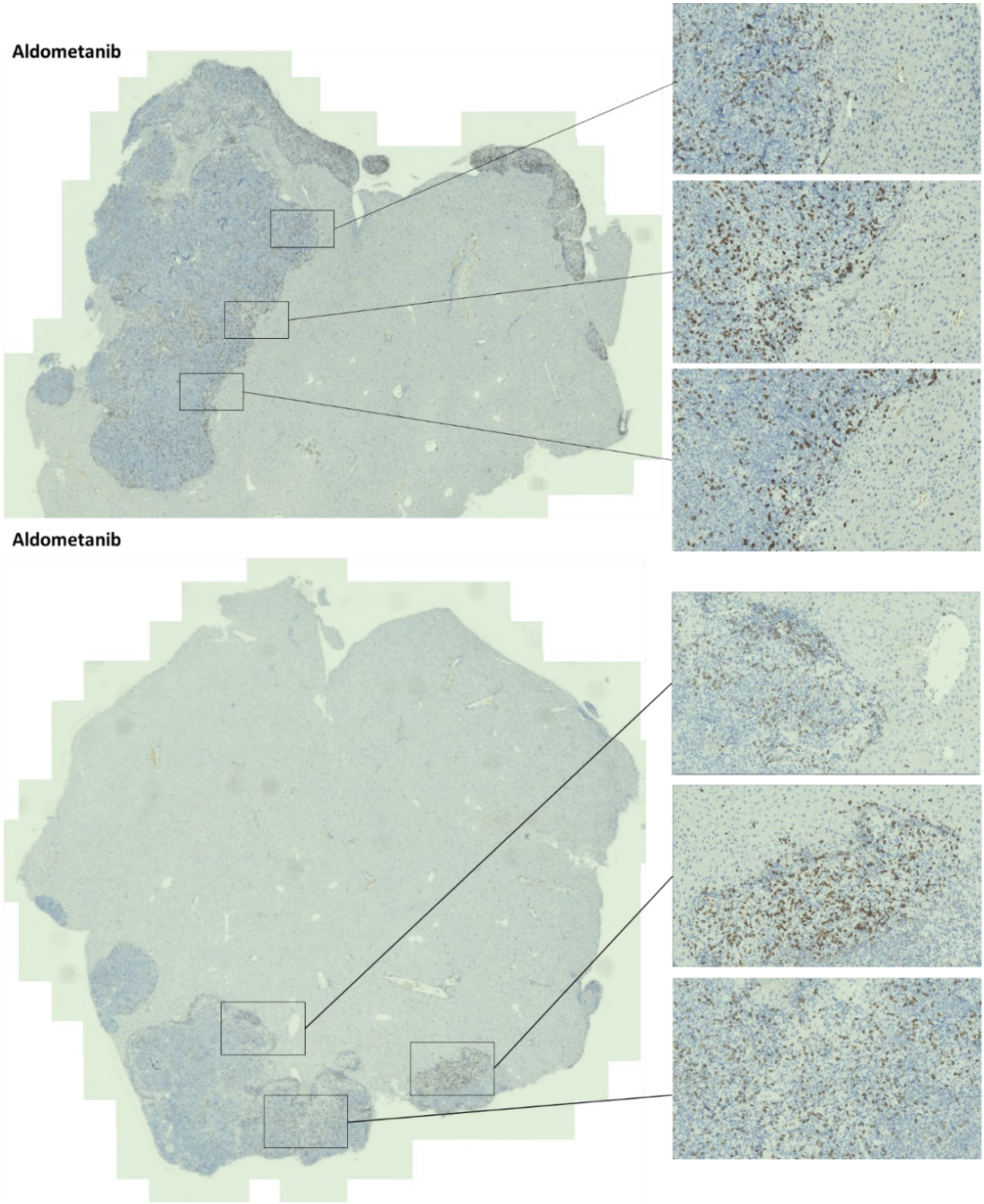

Determination of CD8a in Hepa1-6 allografts

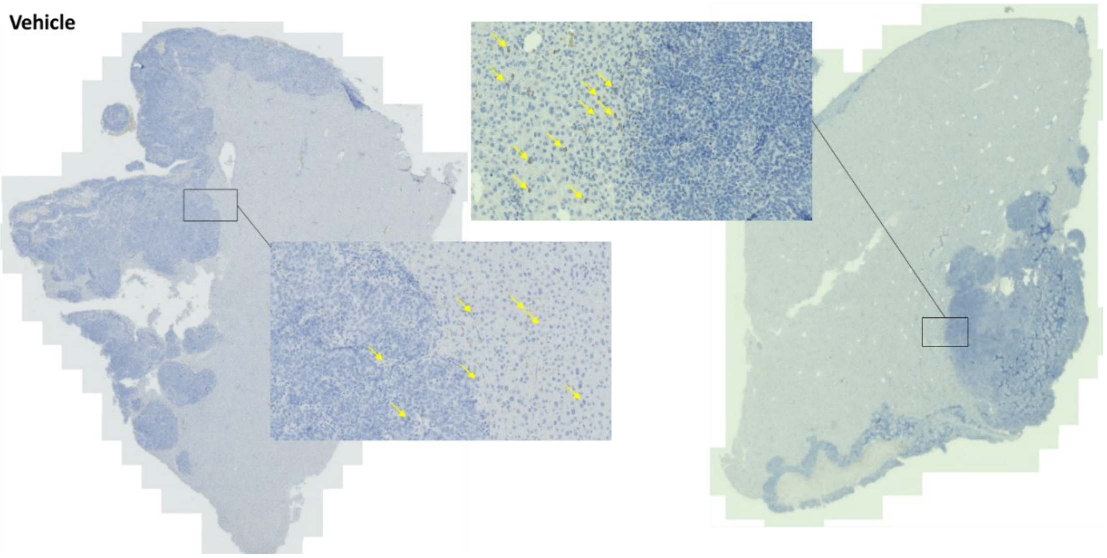

Supplement: Supplementary file 19 — Full scans [file 41422_2025_1195_MOESM19_ESM.pdf]
